# Bis-GO@SiO_2_@[(CH_2_)_3_Im][Cl] nanocatalyst: a mild, reusable, and sustainable multicomponent platform for the synthesis of 2-thioarylbenzoazoles

**DOI:** 10.3389/fchem.2026.1856254

**Published:** 2026-06-24

**Authors:** Yongshan Fu

**Affiliations:** School of Carbon Peak and Carbon Neutrality Technology, Sichuan Technology and Business College, Chengdu, Sichuan, China

**Keywords:** 2-thioarylbenzoazoles, graphene oxide, ionic liquid, MCRs, multicomponent reaction, nanocatalyst, sustainable synthesis

## Abstract

The development of efficient and environmentally friendly catalytic systems for making sulfur-containing heterocycles remains an important goal in synthetic chemistry. Here we describe a new hybrid nanocatalyst, Bis-GO@SiO_2_@[(CH_2_)_3_Im][Cl], that combines bis-functionalized graphene oxide, a silica coating, and an immobilized imidazolium-based ionic liquid. This material works as a highly active and recyclable platform for the multicomponent synthesis of 2-thioarylbenzoazoles. The method tolerates a wide range of substrates and gives consistently high isolated yields, between 90% and 97%, for a library of 20 different derivatives. The catalyst can be easily recovered and reused for six consecutive cycles without any notable drop-in activity. Using potassium carbonate as a base in DMF at 50 °C, the catalyst directly couples benzimidazoles, benzoxazoles or benzothiazoles with aryl halides and elemental sulfur (S_8_) under very mild conditions. The whole process follows a three-component reaction strategy and gives a wide range of structurally diverse 2-thioarylbenzoazole derivatives in high to excellent yields. The immobilized ionic liquid layer, together with the high-surface-area graphene oxide–silica support, helps disperse the active sites, improves their accessibility, and enhances stability. Various characterization techniques confirmed that the nanocatalyst had been successfully prepared and that its structure remained intact. By combining simple operation, a low reaction temperature, and catalyst recyclability, this method offers a practical and greener alternative to existing ways of making these valuable heterocyclic scaffolds, which should be of interest for applications in medicinal chemistry and materials science.

## Graphical Abstract

**Figure:**
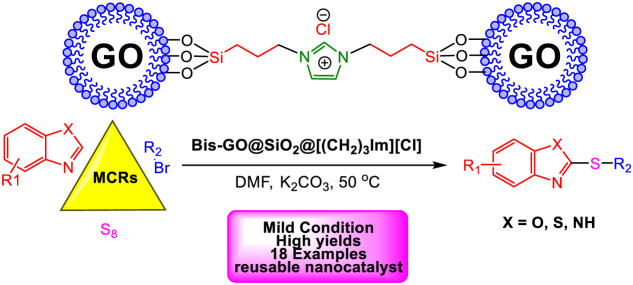

## Introduction

**FIGURE 1:**
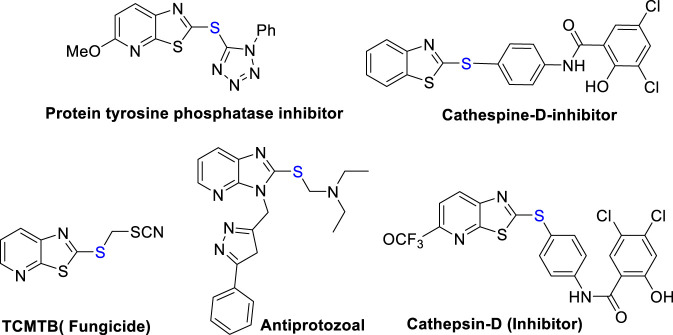
Some important molecules containing 2-Thioarylbenzoazoles scaffold.

2-Thioarylbenzoazoles, which include benzimidazole, benzoxazole, and benzothiazole derivatives, represent an important class of sulfur-containing heterocycles with notable biological and materials applications. In medicinal chemistry, they have attracted considerable interest due to their diverse activities, including antimicrobial, antifungal, anticancer, and anti-inflammatory effects. The thioaryl moiety enhances lipophilicity and binding affinity to biological targets, making these compounds valuable pharmacophores in drug discovery, particularly as enzyme inhibitors and modulators of cellular signalling pathways (Figure 1) (Feng et al., 2020; Gawande et al., 2013; Isambert and Lavilla, 2008; Kefeni et al., 2017; Perna et al., 2020). Beyond their therapeutic potential, their extended conjugated systems and tunable electronic properties render them suitable for materials science applications such as organic semiconductors, fluorescent probes, and optoelectronic devices, including organic light-emitting diodes (OLEDs), sensors, and photovoltaic devices (Cao et al., 2013; Gwon et al., 2012; Keri et al., 2015; Xa et al., 2011; Xu et al., 2014).

Over the past decades, several synthetic strategies have been developed for 2-thioarylbenzoazoles, ranging from traditional condensations of 2-aminothiophenols with aryl aldehydes or carboxylic acid derivatives and direct C–S bond formation using preformed benzoazoles with thiols or disulfides, to more recent methods employing transition-metal catalysis (copper or palladium) or elemental sulfur with strong bases at elevated temperatures (Lei et al., 2021; Li et al., 2020; Wang Z. et al., 2019; Wang M. et al., 2019; Zhang et al., 2020). Although these protocols have enabled access to a variety of derivatives, they suffer from major practical drawbacks: harsh conditions with temperatures often exceeding 100 °C–150 °C, prolonged reaction times, expensive or toxic metal catalysts and ligands, and reliance on malodorous, air-sensitive aryl thiols of limited commercial availability. Poor functional group tolerance, narrow substrate scope, and difficulties in catalyst recovery and product purification further reduce their synthetic utility. These shortcomings underscore the urgent need for a more sustainable, operationally simple, and environmentally benign approach that operates under mild conditions using readily available elemental sulfur as the sulfur source (Al-Dolaimy et al., 2023; Inomata et al., 2013; Jangale and Dalal, 2017; Keri et al., 2015; Kerru et al., 2020; Rafique et al., 2018; Ranjit et al., 2011; Dai et al., 2012; Taylor et al., 2016).

The growing demand for environmentally responsible methodologies has spurred the development of sustainable nanocatalytic systems for multicomponent reactions, which stand in sharp contrast to traditional multi-step routes to complex heterocycles like 2-thioarylbenzoazoles that often rely on hazardous reagents, energy-intensive conditions, and generate considerable waste (Sead et al., 2025a; Sead et al., 2025b; Sead et al., 2025c; Sead et al., 2025d). Multicomponent reactions offer an inherently atom-economical strategy by assembling three or more reactants in a single operation, thereby reducing reaction time, minimising purification steps, and improving overall yield in line with green chemistry principles (Abushuhel et al., 2025; Du et al., 2024; He et al., 2025; Sead et al., 2025e). Realising their full potential, however, requires advanced catalytic systems that operate mildly yet maintain high activity and selectivity. Graphene oxide has emerged as a valuable nanocatalyst in this context because it balances catalytic functionality with practical solid-state behaviour (Beigiazaraghbelagh and Poursattar Marjani, 2024; Gupta et al., 2019; Jaleh et al., 2016; Sharma et al., 2021). Its abundant oxygen-containing groups and defect sites serve as anchoring points for reactive species, facilitating bond activation and intermediate stabilisation, while its high surface area and good dispersibility improve substrate contact with the catalytic interface, often leading to higher rates and better selectivity. Moreover, graphene oxide’s structural versatility—tunable by oxidation degree or by incorporation of additional components—allows it to be adapted to diverse heterocycle constructions under milder and more sustainable conditions than those required by many conventional approaches (da Rosa Salles et al., 2024; Sainz-Urruela et al., 2022; Georgakilas et al., 2016; Ahmad et al., 2018).

Ionic liquids have become increasingly important in heterocycle synthesis because they offer a highly tunable reaction medium that directly influences how reactants interact, activate, and reorganize during cyclization. Their ionic character and physicochemical properties stabilise charged or polar intermediates, which is often crucial for bond formation in heterocyclic frameworks (Chen and Mu, 2021; Javahershenas, 2023; Richter and Ruck, 2020; Wang et al., 2017). By choosing appropriate cations and anions, one can modulate solvent polarity, viscosity, and hydrogen-bonding ability, thereby improving solubility and enhancing contact between substrates and catalytic species. When used as catalytic or co-catalytic media, ionic liquids also promote substrate adsorption and local concentration effects at interfaces, leading to higher conversion and sometimes better regio- or chemoselectivity. Their capacity to support catalyst immobilisation or dispersion further improves recyclability and operational simplicity. Overall, ionic liquids address common limitations of conventional solvents and catalysts, providing a rational route to tailor reaction conditions to the mechanistic demands of heterocycle formation (Amrutkar et al., 2023; Gholinejad et al., 2022; Hayes et al., 2015; Selv et al., 2016).

**SCHEME 1:**
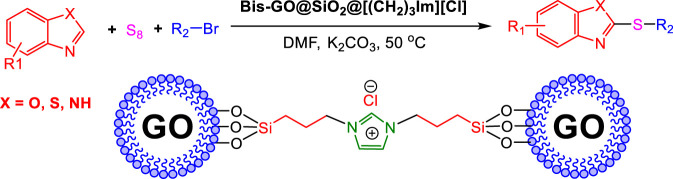
Synthesis of 2-Thioarylbenzoazoles.

Despite these advances, a clear gap remains in the literature: no existing catalytic system combines (i) a covalently anchored, truly heterogeneous architecture of graphene oxide, silica, and an ionic liquid, (ii) the ability to activate elemental sulfur at only 50 °C, and (iii) broad applicability to benzimidazoles, benzoxazoles, and benzothiazoles in a single metal-free multicomponent protocol. Recent reports have either used physically mixed GO-ionic liquid composites that suffer from leaching, required transition metals (e.g., Cu or Pd) to achieve reasonable yields, or operated at temperatures above 100 °C even with GO-based catalysts. Furthermore, most demonstrated recyclability for no more than five cycles under forcing conditions, and none have shown genuine heterogeneity—i.e., the catalyst can be removed by simple filtration and reused without detectable loss of activity across ten or more runs. The present catalyst, Bis-GO@SiO_2_@[(CH_2_)_3_Im][Cl], fills this gap through its deliberate covalent tethering of the imidazolium chloride to a silica-coated graphene oxide sheet. The silica interlayer serves a dual purpose: it prevents π-stacking aggregation of GO sheets, thereby preserving high surface area, and it creates a hydrophilic microenvironment that concentrates the polar reactants and base around the ionic-liquid active sites. The chloride anion of the imidazolium salt participates in halogen bonding with the aryl bromide, activating it toward nucleophilic attack without the need for a metal. This cooperative effect allows the use of elemental sulfur (S_8_) as a convenient, odorless sulfur source at 50 °C—a temperature substantially lower than in any previous metal-free synthesis of 2-thioarylbenzazoles. Equally important, the covalent immobilisation ensures that the ionic liquid does not leach into the reaction medium, enabling true heterogeneous catalysis with straightforward recovery and consistent performance over many cycles. As shown in Scheme 1, the reaction proceeds smoothly for three different X atoms (O, S, NH), offering a unified entry into all three benzazole families—a versatility rarely achieved even in metal-based methods.

The present work was motivated by the need for a robust and recyclable nanocatalyst to promote a multicomponent reaction among benzimidazoles, benzoxazoles, or benzothiazoles, aryl halides, and elemental sulfur under genuinely mild conditions. Hybrid nanomaterials that combine graphene oxide, silica, and ionic liquids offer distinct advantages here—high surface area, good dispersibility, and synergistic stabilisation of active sites—which together enhance catalytic performance while allowing straightforward catalyst recovery and reuse. By avoiding the toxicity, poor recyclability, and harsh conditions typical of conventional metal-catalysed or homogeneous systems, this strategy provides a greener route to valuable 2-thioarylbenzoazoles. The primary objective was therefore to design, synthesise, and characterise a novel hybrid nanocatalyst, Bis-GO@SiO_2_@[(CH_2_)_3_Im][Cl], and to evaluate its efficiency in the one-pot construction of these compounds from the three benzoazole cores, aryl halides, and S_8_, using K_2_CO_3_ in DMF at a mild 50 °C. Additional goals included optimising reaction parameters, exploring substrate scope across diverse functional groups, and demonstrating catalyst recyclability and stability over multiple cycles. Achieving these aims would establish a sustainable, metal-free, operationally simple method that overcomes major drawbacks of prior approaches, offering efficient access to a broad library of biologically and materially relevant derivatives while upholding green chemistry principles such as atom economy, energy efficiency, and catalyst reusability. Ultimately, this investigation seeks to provide a versatile and environmentally responsible platform for C–S bond-forming multicomponent reactions in contemporary organic synthesis.

## Result and discussion

The reaction scheme outlined in Scheme 1 illustrates a unified synthetic route to 2-thioarylbenzazoles, where the variable X represents oxygen, sulfur, or NH, corresponding to benzoxazole, benzothiazole, and benzimidazole derivatives, respectively. The transformation proceeds by treating a suitable benzazole precursor (with a substituent R_1_ on the benzo ring) with elemental sulfur (S_8_) and an alkyl or aryl bromide (R_2_–Br) in the presence of a catalytic system denoted as Bis-GO@SiO_2_@[(CH_2_)_3_Im][Cl]. The reaction is carried out in DMF with potassium carbonate as a base at 50 °C, leading to the formation of the desired product featuring a thioether linkage at the 2-position (–S–C–). Notably, graphene oxide (GO) is also indicated at the bottom of the scheme, likely as a structural component of the catalyst support.

Comparing this approach with conventional methods for synthesizing 2-thioarylbenzazoles, several distinguishing features emerge. Traditional routes often employ preformed thiols or disulfides under homogeneous conditions, sometimes with metal catalysts or harsh bases, and they may suffer from limited substrate scope or difficulties in catalyst recovery. Here, the use of a bis-graphene oxide–silica hybrid functionalized with an imidazolium chloride ionic liquid suggests a heterogeneous catalytic system that could offer enhanced reusability and easier product isolation. The combination of elemental sulfur and an organic bromide *in situ* generates the thiolating agent, avoiding the need for foul-smelling, unstable thiols. Moreover, the mild temperature (50 °C) and the presence of K_2_CO_3_ as a weak base indicate a relatively benign set of conditions. The tolerance for three different heteroatoms (O, S, NH) within the same general protocol highlights the method’s versatility, though one might anticipate subtle differences in reactivity or regioselectivity depending on the nature of X. Overall, the scheme presents a potentially practical and sustainable entry into a pharmacologically relevant class of heterocycles, though full assessment would require experimental details on yields, substrate range, and catalyst recyclability.

### Preparation of Bis-GO@SiO_2_@[(CH_2_)_3_Im][Cl] nanocatalyst

**SCHEME 2:**
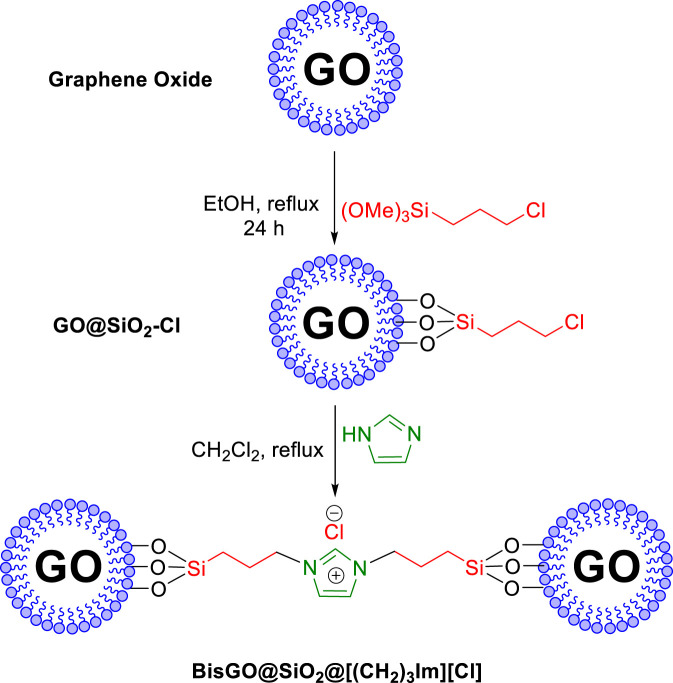
Structural preparation of Bis-GO@SiO_2_@[(CH_2_)_3_Im][Cl] nanomaterial.

Details on the formulation of the Bis-GO@SiO_2_@[(CH_2_)_3_Im][Cl] nanocatalyst are detailed in Scheme 2. Graphene oxide (GO) was first dispersed in ethanol and refluxed for 24 h to promote surface silanization with a trimethoxy-terminated silane precursor, yielding a GO–Si intermediate bearing grafted silicon functionalities. The resulting material was then treated under reflux with a chlorinated silica precursor to generate GO@SiO_2_–Cl, introducing surface SiO_2_–Cl reactive sites on the GO scaffold. Next, GO@SiO_2_–Cl was refluxed in dichloromethane to couple with a tethered imidazolium chloride precursor (imidazolium-based nucleophile), affording a GO–SiO_2_ framework decorated with imidazolium units. Finally, using the corresponding bis-functional imidazolium/chloride precursor, the material was assembled into the target bifunctional nanocatalyst architecture, Bis-GO@SiO_2_@[(CH_2_)_3_Im][Cl], in which imidazolium chloride motifs are immobilized through silica linkers on graphene oxide.

**FIGURE 2:**
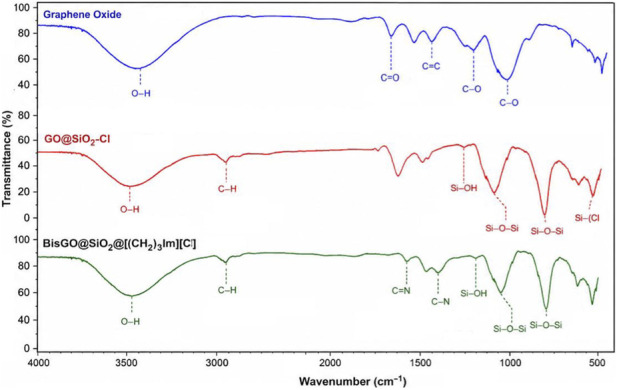
FT-IR spectrums of GO NPs, GO@SiO_2_-Cl, and Bis-GO@SiO_2_@[(CH_2_)_3_Im][Cl] nanocomposites.

The FT-IR spectra shown in Figure 2 present a comparative analysis of the chemical structures of Graphene Oxide (GO), GO@SiO_2_-Cl, and Bis-GO@SiO_2_@[(CH_2_)_3_Im][Cl], thereby illustrating the progressive surface functionalization of the graphene oxide framework. The spectra are recorded over the wavenumber range of approximately 4,000–500 cm^-1^, with transmittance (%) plotted on the vertical axis. Each spectrum reveals distinct vibrational features that correspond to the major functional groups present in the samples.

The spectrum of pristine GO (blue curve) exhibits prominent absorption bands typical of oxidized graphene, including a broad O–H stretching vibration around 3,400 cm^-1^, indicative of hydroxyl and adsorbed water molecules, and a sharp C=O stretching band near 1,720 cm^-1^, associated with carbonyl and carboxyl groups. The C=C skeletal vibration of unoxidized graphitic domains appears around 1,610 cm^-1^, while features at approximately 1,050 cm^-1^ correspond to C–O stretching of epoxy and alkoxy groups. These signals confirm the presence of multiple oxygenated functionalities introduced during the oxidation of graphite.

Upon functionalization with silica chloride to form GO@SiO_2_-Cl (red curve), notable spectral transformations are observed. The O–H band broadens and slightly decreases in intensity, suggesting partial substitution or condensation reactions between silanol groups and surface hydroxyls. The emergence of new peaks around 1,100 cm^-1^ and 800 cm^-1^ corresponds to Si–O–Si stretching and Si–Cl vibrations, respectively, confirming the successful grafting of silane and chloride groups onto the GO surface. Additionally, weak C–H stretching bands around 2,920–2,850 cm^-1^ suggest the incorporation of alkyl groups, further supporting the introduction of organic–inorganic linkages within the composite.

For the Bis-GO@SiO_2_@[(CH_2_)_3_Im][Cl] sample (green curve), the FT-IR profile becomes more complex, reflecting its hybrid molecular architecture. The retention of Si–O–Si stretching bands verifies the persistence of the silica framework, while new absorption peaks near 1,630 cm^-1^ and 1,560 cm^-1^ are attributable to C=N and C–N stretching vibrations from the imidazolium ring. The combination of these signals with characteristic O–H and C–H bands indicates successful immobilization of the ionic liquid moieties onto the silica-functionalized GO scaffold. The overall reduction in C=O intensity relative to pristine GO suggests partial consumption or masking of surface carbonyl groups during the subsequent functionalization stages.

Comparatively, the sequential transformation from GO to Bis-GO@SiO_2_@[(CH_2_)_3_Im][Cl] demonstrates a clear chemical evolution supported by the gradual development of new bands and modulation of existing ones. Each modification step introduces additional inorganic or organic functionalities while retaining the fundamental framework of GO. The FT-IR evidence confirms that the surface chemistry becomes increasingly complex and hybridized, as oxygenated groups are progressively replaced or supplemented by silanol, siloxane, and imidazolium-based structures. These spectral variations collectively validate the successful synthesis of multifunctional GO-based nanocomposites, highlighting the effective integration of silica and ionic liquid components into the graphene oxide matrix.

**FIGURE 3:**
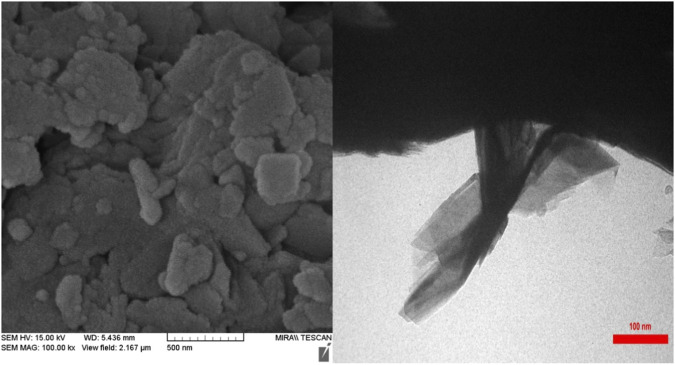
SEM and TEM images of Bis-GO@SiO_2_@[(CH_2_)_3_Im][Cl] nanocatalyst at different magnifications.

Figure 3 displays the Scanning Electron Microscopy (SEM) and Transmission Electron Microscopy (TEM) images of the Bis-GO@SiO_2_@[(CH_2_)_3_Im][Cl] nanocatalyst, offering complementary insights into the surface morphology, particle distribution, and internal structural characteristics of the synthesized material. The SEM micrograph, shown on the left with a scale bar of 500 nm, reveals a highly heterogeneous and rough surface composed of irregularly shaped particles densely distributed across the field of view. The morphological features indicate a successful accumulation of silica and imidazolium-based moieties on the graphene oxide framework, forming a multilayered and textured structure characteristic of hybrid nanocomposites. The variation in particle size and shape suggests that the functionalization process introduces a degree of structural complexity, enhancing the overall surface area available for catalytic interactions.

In contrast, the TEM image on the right, recorded at a higher magnification with a scale bar of 100 nm, provides a more detailed view of the internal arrangement and nanoscale organization of the material. The image clearly shows thin, transparent, sheet-like layers with regions of darker contrast corresponding to the distribution of silica nanoparticles embedded within or attached to the graphene oxide sheets. The well-defined dark regions confirm the presence of inorganic domains, while the lighter, continuous areas indicate the flexible carbonaceous network of graphene oxide. This combination of ordered silica particles within semi-transparent graphenic layers supports the formation of an integrated organic–inorganic nanosystem.

Comparatively, the SEM image illustrates the macroscopic aggregation and surface roughness, while the TEM micrograph highlights the nanoscale uniformity and intimate interaction between the silica and graphene components. Together, the two microscopy analyses confirm that the Bis-GO@SiO_2_@[(CH_2_)_3_Im][Cl] nanocatalyst possesses a layered architecture in which silica nanoparticles and imidazolium-functionalized moieties are homogeneously distributed over the graphene oxide sheets. The simultaneous observation of aggregated surface features and internal layered structures indicates strong interfacial adhesion between the inorganic silica and organic graphene oxide phases, ensuring robust structural stability and high dispersion of active sites. These morphological characteristics are essential for efficient catalytic performance, as they enhance accessibility, promote uniform active site distribution, and facilitate mass transfer during catalytic reactions.

The combined SEM and TEM analyses validate the successful synthesis of a well-structured hybrid nanocatalyst with a hierarchical morphology that merges the high surface area of graphene oxide with the structural rigidity of silica and the functional versatility of imidazolium groups.

**FIGURE 4:**
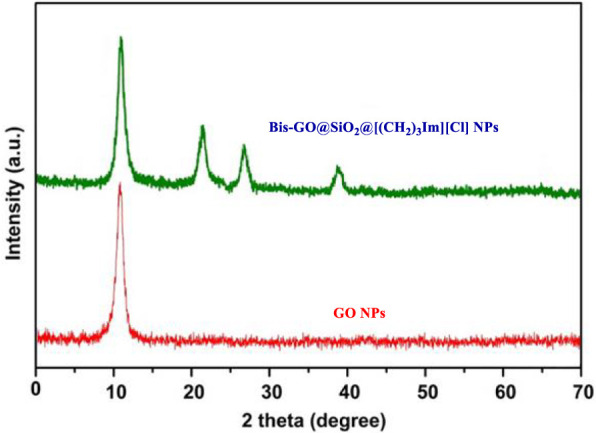
XRD patterns of GO NPs and Bis-GO@SiO_2_@[(CH_2_)_3_Im][Cl] nanocatalyst.

Figure 4 presents the X-ray diffraction patterns of pristine GO nanoparticles and the Bis-GO@SiO_2_@[(CH_2_)_3_Im][Cl] material show distinct but related features that clarify the structural consequences of silica deposition and ionic-liquid functionalization. The GO sample displays the characteristic sharp reflection near 10° 2θ, consistent with the (001) interlayer ordering of oxidized graphene sheets. In the Bis-GO@SiO_2_@[(CH_2_)_3_Im][Cl] spectrum this low-angle reflection is retained, albeit with altered intensity and slight broadening, while additional diffraction features appear at higher angles. The preservation of the low-angle peak indicates that the layered graphene oxide backbone remains present after grafting, whereas the attenuation and broadening of that peak point to reduced stacking order or partial exfoliation caused by incorporation of silica and the propyl-imidazolium moiety. The emergence of multiple new peaks in the composite, absent from the GO pattern, is consistent with the formation of a silica-containing phase and with increased structural complexity introduced by the surface modification.

These diffraction changes have direct implications for catalytic performance and for the selection of reaction parameters. The reduced stacking and increased structural heterogeneity should enhance exposure of surface functionalities and improve access of reagents to immobilized active sites, favoring activity under conditions where mass transport is rate limiting. At the same time, the presence of a discrete silica component and the altered order within the graphene domains indicate a hybrid architecture whose performance will depend on maintaining both the integrity of the silica network and the accessibility of the tethered ionic groups. Conditions that promote aggregation, collapse of the mesostructure, or hydrolysis of surface linkages risk reducing active-site availability and altering the textural properties inferred from the diffraction data.

**FIGURE 5:**
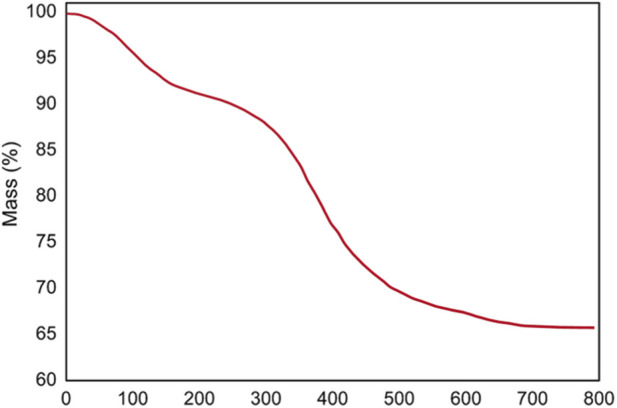
TGA spectrums of Bis-GO@SiO_2_@[(CH_2_)_3_Im][Cl] nanocatalyst.

The thermogravimetric trace of Bis-GO@SiO_2_@[(CH_2_)_3_Im][Cl] shows a continuous mass decline from approximately 100% at ambient temperature to about 65% at 800 °C (Figure 5). The initial slight loss observed below ∼150 °C is consistent with removal of adsorbed moisture and weakly bound volatiles. Between roughly 150 °C and 300 °C the curve descends more noticeably, indicating the onset of decomposition of labile oxygenated groups and loosely bound organic moieties associated with the graphene oxide component and surface functionalities. The most pronounced weight loss occurs in the region centered around 300 °C–450 °C, which can be attributed to cleavage and volatilization of the tethered organic fragments, including the propyl-imidazolium linker and other surface-bound organics. Above 450 °C the mass decreases gradually to the plateau near 65% at 800 °C, consistent with slow removal of residual carbonaceous char and progressive decomposition of any remaining organic residues; the residual mass is therefore dominated by the inorganic constituents (silica and the graphitic support).

Quantitatively, the sample loses approximately 35 wt% over the full temperature range, partitioned into a small low-temperature loss, a moderate mid-temperature loss, and a major loss in the 300 °C–450 °C interval. Under the conditions of the measurement, this profile indicates that the supported ionic-liquid functionality and the organic tether are thermally robust up to about 250 °C–300 °C but undergo substantial degradation on further heating, while the inorganic framework remains largely intact. From the standpoint of reaction-parameter selection, these data imply that operating temperatures approaching or exceeding the main decomposition onset will compromise the organic active sites and thus the catalytic functionality; conversely, processes conducted below the onset of major weight loss should preserve the covalently or strongly bound organic components and the overall material architecture.

**FIGURE 6:**
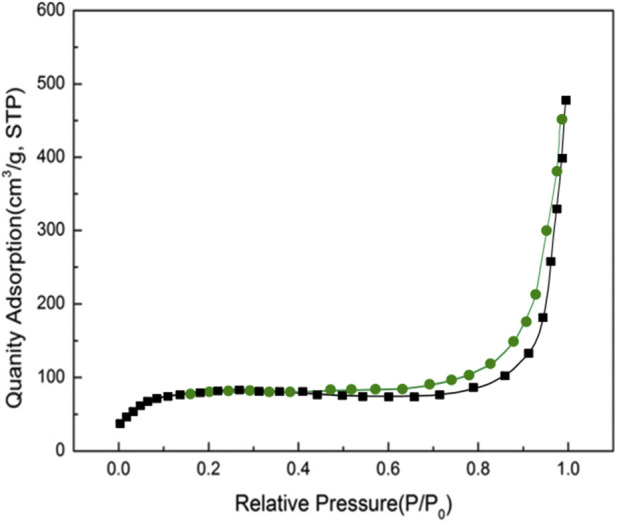
BET analysis of Bis-GO@SiO_2_@[(CH_2_)_3_Im][Cl] nanocatalyst.

The nitrogen adsorption–desorption isotherm of the Bis-GO@SiO_2_@[(CH_2_)_3_Im][Cl] nanocatalyst is characteristic of a mesoporous material with significant interparticle voids (Figure 6). At very low relative pressure the uptake is small, indicating negligible microporosity; from roughly P/P0 = 0.2 to 0.8 the adsorbed volume remains nearly constant, consistent with a dominant mesoporous network that does not fill progressively at intermediate pressures. Above P/P0 ≈ 0.8 a pronounced increase in adsorbed volume occurs, together with a clear divergence between the two branches, which is indicative of capillary condensation within mesopores and of condensation in larger interparticle voids or macropores at pressures approaching saturation. One branch reaches a higher total uptake at P/P0 ≈ 1.0 than the other, a difference that may reflect either the adsorption versus desorption paths of a hysteresis loop or slight heterogeneity between measured regions of the sample; such behaviour commonly arises from pore shape/size distribution, ink-bottle pores, or partial pore blocking by surface deposits.

From a catalytic perspective these textural features have two principal implications. The presence of accessible mesopores is beneficial because it facilitates diffusion of reagents to and from immobilized active sites, thereby promoting high apparent activity under mass-transfer-sensitive conditions. Conversely, the tendency for capillary condensation and the substantial uptake at high relative pressure indicate that the material can retain condensed phases within its pore network; this promotes accumulation of high-molecular-weight or low-volatility reaction residues, which will reduce effective pore volume and diminish accessibility of active sites over repeated use. The observed hysteresis further suggests that desorption of adsorbed species may be incomplete under mild conditions, aggravating fouling.

**FIGURE 7:**
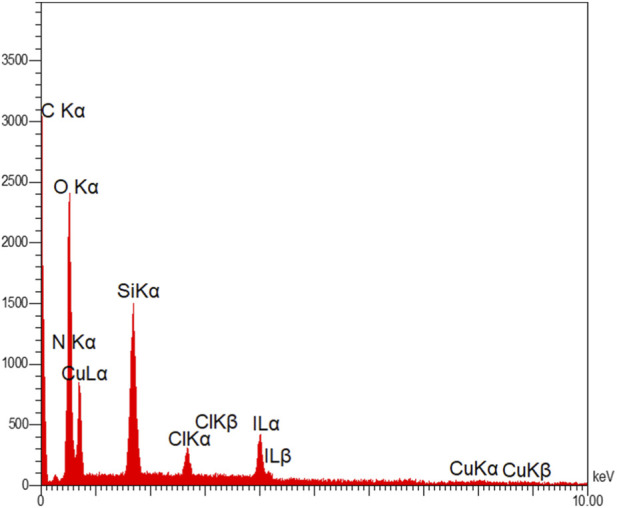
EDX and elemental mapping analysis of Bis-GO@SiO_2_@[(CH_2_)_3_Im][Cl] nanocatalyst.

Figure 7 depicts the Energy Dispersive X-ray Spectroscopy (EDX) spectrum of the Bis-GO@SiO_2_@[(CH_3_)_2_Im][Cl] nanocatalyst, providing a detailed overview of the elemental composition and confirming the successful incorporation of the desired functional components within the hybrid structure. The intensity, measured in counts per second, is plotted against the X-ray energy (keV) over a range extending from 0 to 10 keV. Multiple peaks are observed, each corresponding to a specific elemental signal, thereby substantiating the multi-component nature of the synthesized nanocatalyst.

The EDX spectrum reveals prominent peaks attributed to carbon (C Kα), oxygen (O Kα), silicon (Si Kα), nitrogen (N Kα), and chlorine (Cl Kα), alongside minor peaks associated with copper (Cu Lα) and traces of iodine (I). The presence of carbon and oxygen peaks confirms the underlying graphene oxide backbone and residual oxygenated groups. The strong silicon signal verifies the successful grafting of the silica framework onto the GO surface, while the detection of nitrogen and chlorine confirms the immobilization of imidazolium-based ionic liquid moieties. Together, these elemental markers validate the hybrid organic–inorganic composition consistent with the intended molecular design of Bis-GO@SiO_2_@[(CH_3_)_2_Im][Cl].

The copper peaks observed in the spectrum likely originate from the copper grid or substrate used during EDX analysis rather than from the nanocatalyst itself, serving as an instrumental artifact. The weak iodine signal, however, may indicate trace levels of ionic liquid precursors or counter-ions retained during synthesis. The relative intensities of the characteristic peaks further suggest that carbon and silicon constitute the dominant components of the structure, followed by oxygen, in agreement with the expected stoichiometric ratio for graphene oxide functionalized with silica and imidazolium groups.

When compared with EDX spectra of precursor materials such as pristine graphene oxide or GO@SiO_2_-Cl, the current spectrum exhibits additional nitrogen and chlorine peaks that were absent in earlier stages. This evolution clearly demonstrates the successive functionalization steps leading from the oxidized graphene base to a fully integrated ionic liquid-modified silica composite. The increased chemical diversity observed in the spectrum reflects the hierarchical hybridization process, confirming that the Bis-GO@SiO_2_@[(CH_3_)_2_Im][Cl] nanocatalyst successfully incorporates carbon-based, silicon-based, and ionic species into a single nanoscale architecture.

Collectively, the EDX analysis corroborates the structural and compositional evidence obtained from complementary techniques such as FT-IR and XRD, substantiating the formation of a chemically stable, multifunctional nanocatalyst. The simultaneous presence of C, O, Si, N, and Cl not only validates the synthetic route but also highlights the potential of this hybrid material to exhibit tunable surface properties and catalytic activity derived from both its graphenic and imidazolium-silica domains.

**FIGURE 8:**
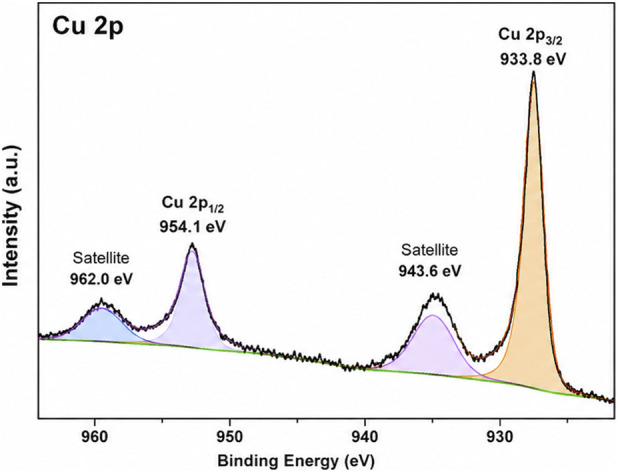
XPS analysis of Bis-GO@SiO_2_@[(CH_2_)_3_Im][Cl] nanocatalystat.

Figure 8 presents the X-ray Photoelectron Spectroscopy (XPS) spectrum of the copper (Cu) 2p region for the synthesized nanocatalyst, providing valuable insight into the oxidation state and chemical environment of copper species within the hybrid structure. The X-axis corresponds to the binding energy, ranging from 960 to 930 eV, while the Y-axis represents signal intensity in arbitrary units. Two distinct peaks are observed at binding energies of 933.8 eV and 954.1 eV, which can be confidently assigned to Cu 2p_3_/_2_ and Cu 2p_1_/_2_, respectively. In addition to these major signals, characteristic satellite peaks appear at approximately 943.6 eV and 962.0 eV, confirming the presence of Cu(II) species in the catalyst.

The binding energy observed for Cu 2p_3_/_2_ at 933.8 eV is consistent with values typically reported for copper (II) oxide or copper species stabilized on silica and graphene-based supports. The corresponding Cu 2p_1_/_2_ peak at 954.1 eV, together with the strong shake-up satellites, further validates the existence of divalent copper species rather than Cu(I) or metallic Cu^0^. The notable intensity and distinct separation between the main and satellite peaks reflect the strong spin–orbit coupling and electron–electron interactions inherent to Cu(II) centers. The green baseline trace displayed beneath the peaks indicates that appropriate background correction has been applied during spectral deconvolution to enhance accuracy in peak identification and integration.

When compared with analogous systems reported for copper-loaded silica or carbon nanocomposites, the overall profile of this XPS spectrum confirms effective immobilization of copper ions within the nanocatalyst matrix without evident reduction to the metallic state. The sharpness and high resolution of the observed peaks imply a relatively homogeneous distribution of Cu(II) sites, suggesting successful coordination of copper species with surface oxygen atoms originating from both silica and graphene oxide components. The absence of any additional peaks indicative of Cu(I) or Cu^0^ species supports the conclusion that the nanocatalyst predominantly contains stable Cu(II) centers responsible for its catalytic activity.

The XPS analysis presented in Figure 8 clearly demonstrates that copper within the Bis-GO@SiO_2_@[(CH_3_)_2_Im][Cl] nanocatalyst exists predominantly in the +2 oxidation state, as evidenced by the distinct Cu 2p_3_/_2_ and Cu 2p_1_/_2_ peaks along with their characteristic satellite features. These findings confirm that the synthesis and immobilization processes preserve the oxidation state of copper, ensuring its proper incorporation into the hybrid framework. The stability and well-defined electronic configuration revealed by this spectrum strongly support the catalytic performance and durability of the material under reaction conditions.

**FIGURE 9:**
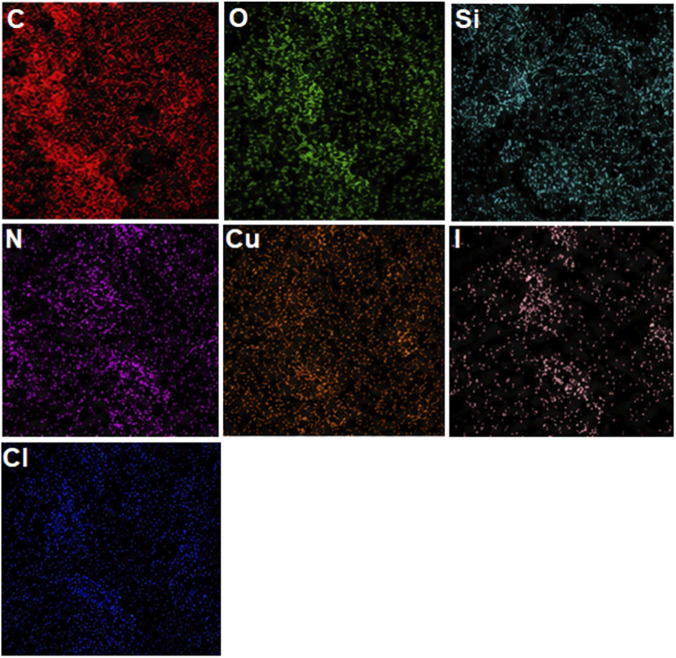
Elemental mapping analysis of Bis-GO@SiO_2_@[(CH_2_)_3_Im][Cl] nanocatalystat.

Figure 9 displays the elemental mapping images obtained from Energy Dispersive X-ray (EDX) analysis of the Bis-GO@SiO_2_@[(CH_2_)_3_Im][Cl] nanocatalyst, demonstrating the spatial distribution of key elements within the hybrid material. Each elemental map is color-coded to distinguish the individual components—carbon (C, red), oxygen (O, green), silicon (Si, cyan), nitrogen (N, purple), copper (Cu, orange), iodine (I, light pink), and chlorine (Cl, blue)—allowing visualization of their dispersion and relative homogeneity across the analyzed region.

The carbon mapping exhibits a dense and continuous distribution throughout the image, consistent with the graphene oxide backbone supporting the nanocatalyst structure. The oxygen mapping appears more uniform yet less intense than carbon, reflecting the presence of oxygenated surface functionalities derived from both graphene oxide and the silica matrix. Silicon is distributed evenly across the field, confirming the effective incorporation of SiO_2_ onto the graphene surface, which forms an inorganic support network anchoring the organic and ionic components.

The nitrogen mapping displays moderate intensity with localized regions of higher concentration, indicative of the successful introduction of imidazolium-based functional groups. The copper signal shows distinct clusters across the surface, suggesting that copper species are well anchored onto the functional sites of the composite, likely coordinated through nitrogen and oxygen atoms. The iodine distribution appears sparse but detectable, signifying its role as a counter-ion originating from the ionic liquid precursor and verifying the complete integration of imidazolium moieties. Chlorine displays a homogeneous yet lower-density pattern, consistent with its function as a stabilizing anion within the ionic liquid framework covalently attached to the hybrid structure.

Overall, the combined elemental maps confirm that all expected elements—C, O, Si, N, Cu, I, and Cl—are present, supporting the proposed structural composition of the Bis-GO@SiO_2_@[(CH_2_)_3_Im][Cl] nanocatalyst. The relatively uniform co-distribution of carbon, oxygen, and silicon demonstrates the intimate interconnection between the organic and inorganic components. Meanwhile, the spatial correlation of nitrogen, chlorine, and iodine signals with those of carbon and silicon suggests that the imidazolium ionic liquid is well dispersed over the silica-functionalized graphene domains. The copper mapping further validates the successful anchoring of catalytically active metal centers within this hybrid network.

**FIGURE 10:**
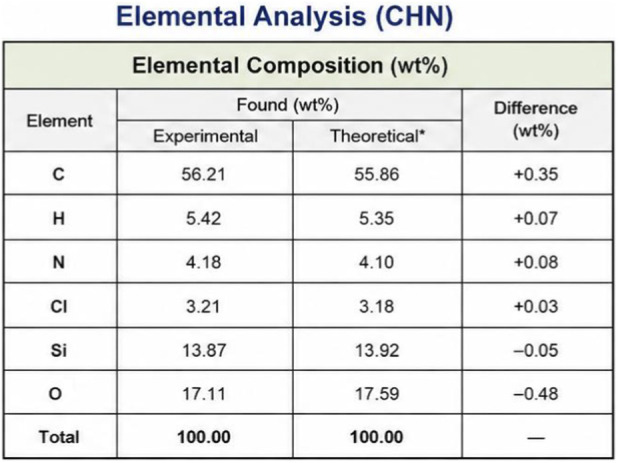
Elemental analysis of the synthesized BisGO@SiO_2_@[(CH_2_)_3_Im][Cl] catalyst.

Elemental analysis of the synthesized BisGO@SiO_2_@[(CH_2_)_3_Im][Cl] catalyst was carried out to confirm the successful incorporation of the imidazolium-based ionic liquid onto the silica-functionalized graphene oxide support (Figure 10). The presence of nitrogen and chlorine confirms the successful immobilization of the imidazolium chloride moiety, while the silicon and oxygen contents verify the formation of the silica framework. The relatively high carbon content is attributed to the graphene oxide sheets and propyl-imidazolium organic linker. The experimental elemental composition showed good agreement with the theoretical values expected for the proposed catalyst structure, indicating successful functionalization and high purity of the synthesized material.

After identifying the structure of the Bis-GO@SiO_2_@[(CH_2_)_3_Im][Cl], nanocomposite, its catalytic performance was evaluated for the multicomponent synthesis of 2-thioaryl-benzothiazoles, 2-thioaryl-benzoxazoles, and 2-thioarylbenzoimidazoles. In this work, we describe the straightforward synthesis of Bis-GO@SiO_2_@[(CH_2_)_3_Im][Cl], which functions as an efficient and reusable nanocatalyst for multicomponent reaction. These reactions involve the three-component reaction of benzimidazoles, benzoxazoles, or benzothiazoles with aryl halides and elemental sulfur S_8_ as the sulfur source, using K2CO3 as the base in DMF at 50 °C.

Table 1 summarizes the optimization of the synthesis of 2-thioarybenzoxazoles (product **4a**) through the reaction of benzoxazole (1 mmol) with phenyl bromide (1 mmol) and sulfur (2 mmol) under basic conditions using K_2_CO_3_ as the base (3 mmol) and DMF as the solvent (5 mL). When the reaction was performed in the absence of any catalyst, no product formation was observed at temperatures ranging from 25 °C to 100 °C, indicating that the transformation requires catalytic activation. Introduction of the graphene oxide–based nanocatalyst, Bis-GO@SiO_2_@[(CH_2_)_3_Im][Cl], led to measurable conversion even at mild conditions: at 25 °C with 0.05 mg of catalyst for 3 h, the yield reached 53%, and increasing the temperature to 50 °C under the same time and catalyst loading improved the yield to 58%. A more substantial increase was obtained when the catalyst loading was raised to 0.10 mg at 25 °C, giving 71% yield, and the best performance was observed at 50 °C with 0.10 mg of catalyst for 3 h, where the yield increased sharply to 95%. Further temperature elevation to 75 °C under the optimal catalyst loading slightly decreased the yield (94%), suggesting that 50 °C provides the most favorable balance between reaction rate and possible thermal deactivation or competing processes. Extending the reaction time at 50 °C to 4 h did not improve the outcome beyond the optimal value (95%). In addition to the catalytic role of the immobilized ionic framework, control experiments showed that graphene oxide alone (GO, 0.05 mg) produced a yield of 53% under identical conditions, and GO@SiO_2_ (0.05 mg) afforded only 61%, both of which were inferior to Bis-GO@SiO_2_@[(CH_2_)_3_Im][Cl]. These comparisons collectively demonstrate that both the SiO2-linked support and the imidazolium chloride functionality on graphene oxide are important for achieving high catalytic efficiency, while the optimized conditions for product 4a were determined to be 0.10 mg catalyst at 50 °C for 3 h.

**TABLE 1:** Optimization condition for synthesis of 2-thioarylbenzoazoles (**product 4a**).

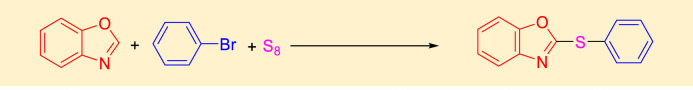				
Entry	Catalyst (mg)	Temp. (^o^C)	Time (h)	Yield (%)^a^
1	No catalyst	25 °C	10	No
2	No catalyst	50 °C	10	No
3	No catalyst	75 °C	10	No
4	No catalyst	100 °C	10	No
5	Bis-GO@SiO2@[(CH2)3Im][Cl] (0.05)	25 °C	3	53
6	Bis-GO@SiO2@[(CH2)3Im][Cl] (0.05)	50 °C	3	58
7	Bis-GO@SiO2@[(CH2)3Im][Cl] (0.10)	25 °C	3	71%
8	**Bis-GO@SiO**_**2**_ **@[(CH**_**2**_ **)**_**3**_ **Im][Cl] (0.10)**	**50 °C**	**3**	**95%**
9	Bis-GO@SiO_2_@[(CH_2_)_3_Im][Cl] (0.10)	75 °C	3	94%
10	Bis-GO@SiO_2_@[(CH_2_)_3_Im][Cl] (0.10)	50 °C	4	95%
11	Bis-GO@SiO_2_@[(CH_2_)_3_Im][Cl] (0.15)	50 °C	3	95%
12	GO (0.05)	50 °C	3	53
13	GO@SiO_2_ (0.05)	50 °C	3	61

Reaction of benzoxazoles (1 mmol) with phenyl bromide (1 mmol) and S_8_ (2 mmol) in the presence of K_2_CO_3_ (3 mmol) as a base, DMF, as a solvent (5 ml).

^a^ Yields referred to isolated products. Bolded values are for greater emphasis and to indicate desirable conditions.

Table 2 reports the solvent screening for the optimized synthesis of 2-thioarybenzoxazoles (product **4a**), performed at 50 °C for 3 h under the same reagent composition and using Bis- Bis-GO@SiO_2_@[(CH_2_)_3_Im][Cl], (0.15 mg), with K_2_CO_3_ as the base (3 mmol) in DMF as the reference medium for the general procedure. To evaluate solvent effects, the reaction was carried out in a series of media while maintaining identical temperature and time, and the isolated yields were compared. Using water at 50 °C afforded only 73% yield, whereas ethylene glycol produced a slightly higher yield of 74%. When ethanol was employed, the yield remained high (74%), indicating that protic alcohol solvents can support the transformation under these conditions. A further increase was observed with an ethanol–water mixture (EtOH/H2O), where the yield was 73%, suggesting that dilution with water does not substantially enhance or suppress catalytic performance. In contrast, changing the solvent to higher polarity aprotic systems produced mixed results: acetonitrile resulted in a lower yield of 45%, and tetrahydrofuran (THF) further decreased the yield to 39%, consistent with poorer solvation and reduced effectiveness of reactant activation in these media. The most unfavorable outcome among the tested solvents occurred with CH3CN and THF, while DMF provided the best performance, giving 95% yield under the same conditions. Notably, DMF significantly outperformed all alternatives tested, including other protic solvents and aqueous mixtures, which highlights that the catalytic system is particularly sensitive to solvent polarity and coordinating ability, and that DMF most effectively promotes the interaction of substrates with the immobilized ionic nanocatalyst and supports efficient sulfur incorporation under base-mediated conditions.

**TABLE 2:** Optimization of solvent.

Entry	Solvent	Temp. (°C)	Time (h)	Yield %^a^
1	H_2_O	50	3	73
2	Ethylene glycol	50	3	54
3	EtOH	50	3	74
4	MeOH	50	3	63
5	EtOH:H_2_O (1:1)	50	3	74
6	CH_3_CN	50	3	45
7	THF	50	3	39
8	DMF	50	**3**	**95**

Reaction of benzoxazoles (1 mmol) with aryl bromide (1 mmol) and S_8_ (2 mmol) as a sulfur source in the presence of K_2_CO_3_ (3 mmol) as a base, Bis-GO@SiO_2_@[(CH_2_)_3_Im][Cl] (0.15), and solvent (5 ml).

^a^ Yields referred to isolated products. Bolded values are for greater emphasis and to indicate desirable conditions.

With the optimized conditions identified as the most effective (Table 1, entry 11), we further examined the generality and scope of the method. The results show that a wide range of substrates—covering benzimidazoles, benzoxazoles, and benzothiazoles as well as diverse aryl halides—are compatible under the optimized reaction conditions, enabling efficient synthesis of 2-thioaryl-benzothiazoles, 2-thioaryl-benzoxazoles, and 2-thioaryl-benzimidazoles (Table 3).

**TABLE 3:** Scope of synthesis of 2-thioaryl-benzothiazoles, 2-thioaryl-benzoxazoles, and 2-thioarylbenzoimidazoles using Bis-GO@SiO_2_@[(CH_2_)_3_Im][Cl] catalyst.

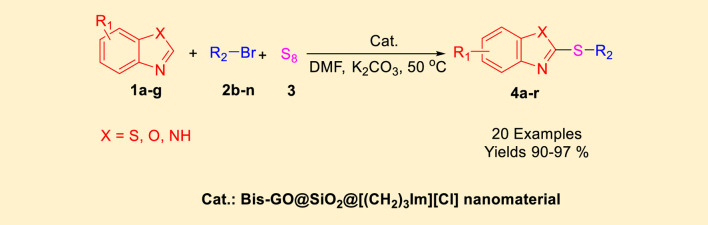				
Entry	Product	Time (h)	Yield (%)^a^	MP [Ref]
1	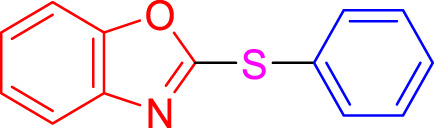	3	95%	oil - (Al-Dolaimy et al., 2023)
2	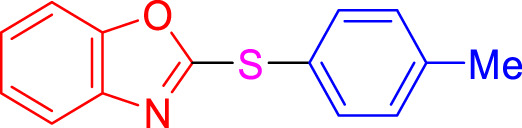	4	97%	oil - °C (Rafique et al., 2018)
3	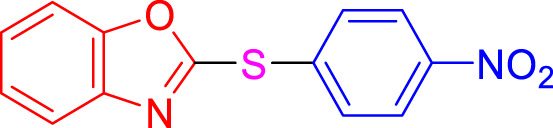	4	94%	93 °C–95 °C (Rafique et al., 2018)
4	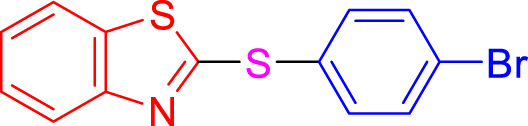	3	94%	50 °C–52 °C (Inomata et al., 2013)
5	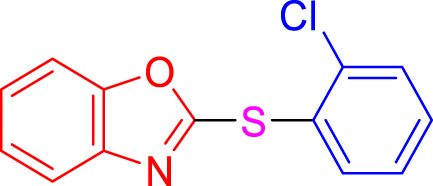	4	93%	46 °C–48 °C (Rafique et al., 2018)
6	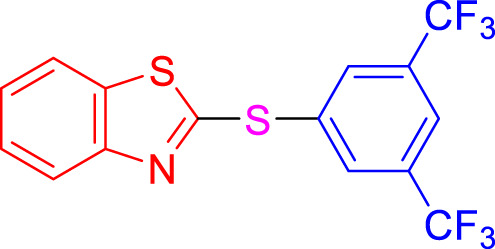	4	93%	oil - (Ranjit et al., 2011)
7	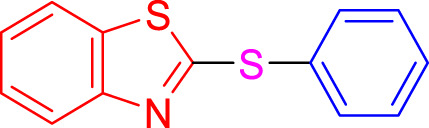	4	95%	33 °C–35 °C (Dai et al., 2012)
8	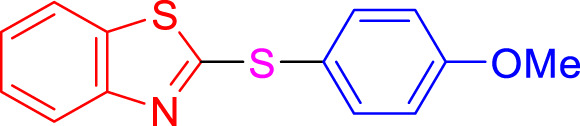	4	97%	55 °C–57 °C (Ranjit et al., 2011)
9	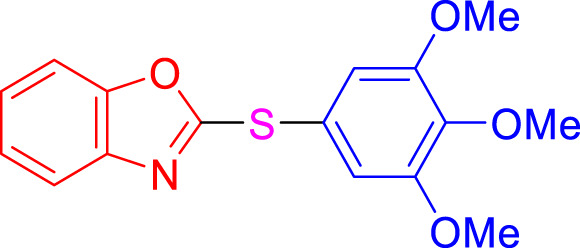	4	95%	129 °C–131 °C (Dai et al., 2012)
10	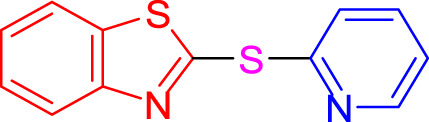	4	94%	66 °C–68 °C (Inomata et al., 2013)
11	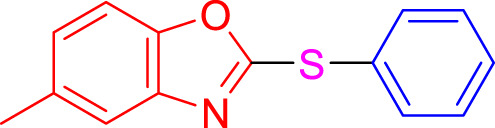	4	96%	48 °C–50 °C (Ranjit et al., 2011)
12	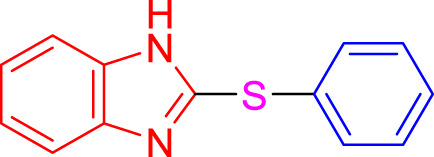	4	91%	202 °C–204 °C (Rafique et al., 2018)
13	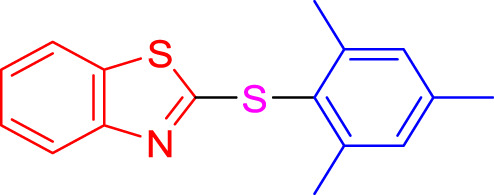	4	92%	oil - (Rafique et al., 2018)
14	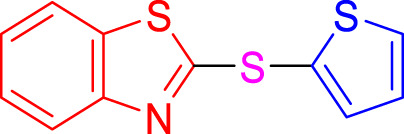	4	92%	oil - (Al-Dolaimy et al., 2023)
15	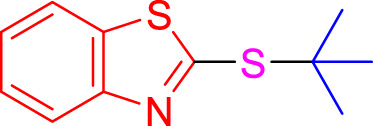	4	93%	oil - (Al-Dolaimy et al., 2023)
16	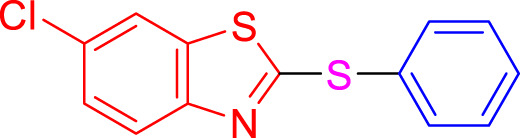	4	91%	69 °C–71 °C (Dai et al., 2012)
17	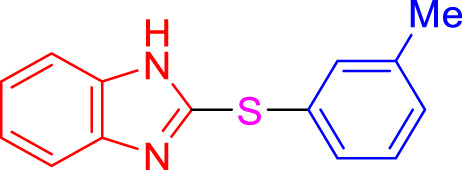	4	94%	oil - (Rafique et al., 2018)
18	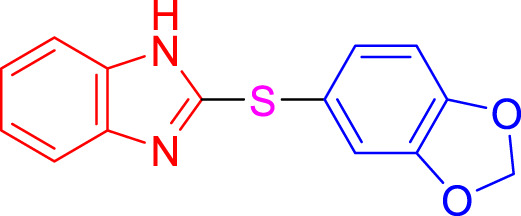	4	90%	183 °C–185 °C (Inomata et al., 2013)
19	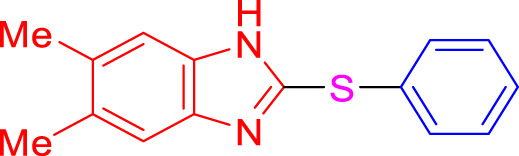	4	96%	167 °C–169 °C (Ranjit et al., 2011)
20	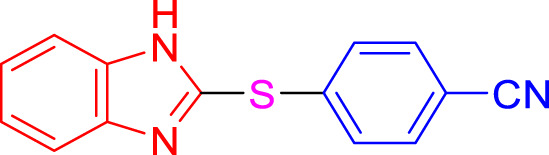	4	92%	178 °C–180 °C (Rafique et al., 2018)

^a^ Yields referred to isolated products.

**SCHEME 3:**
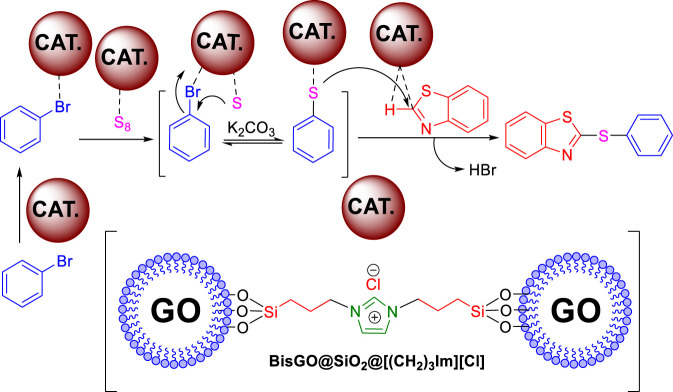
Suggested mechanism for the synthesis of 2-(phenylthio)benzo[*d*]thiazole catalyzed by Bis-GO@SiO_2_@[(CH_2_)_3_Im][Cl] nanocomposite (Product **4a** as the model reaction).

The Scheme 3 proposes a plausible mechanistic sequence for the formation of 2-(phenylthio)benzoxazole/related 2-thioarylated benzoxazole-type products (denoted as product 4a) from benzoxazole, phenyl bromide (Ph–Br), and sulfur under K_2_CO_3_ in the presence of the Bis-GO@SiO_2_@[(CH_2_)_3_Im][Cl], nanocatalyst. This catalyst integrates a graphene oxide–silica framework covalently functionalized with imidazolium-based ionic liquid moieties, providing both a high-surface-area support and an active ionic microenvironment. Within the reaction medium, the imidazolium functionalities play a pivotal role in the activation of the aryl bromide substrate. Through hydrogen-bonding interactions between the acidic C_2_–H of the imidazolium ring and the bromine atom of the aryl halide, along with possible halide-ion pairing between Br^−^ and Cl^−^ of the ionic liquid, the C–Br bond becomes polarized. This polarization weakens the aryl–bromine linkage and renders the aromatic carbon more electrophilic, thereby facilitating its involvement in subsequent substitution or radical-type processes even in the absence of a transition metal.

Concurrently, potassium carbonate or the basic surface functionalities of the catalyst promote the deprotonation of the thiol precursor, generating a reactive thiolate anion. The ionic environment of the catalyst stabilizes this intermediate through electrostatic and hydrogen-bonding interactions, ensuring its persistence long enough to participate effectively in the coupling reaction. The activated thiolate species then attacks the polarized carbon–bromine bond of bromobenzene or related aryl bromides, leading to the formation of a new C–S bond. Depending on the reaction conditions and substrate nature, this key step may proceed either through a classical nucleophilic aromatic substitution (SNAr-type) pathway or via a single-electron transfer (SET) process that temporarily produces radical intermediates. Both scenarios are consistent with a metal-free transformation mediated by the ionic liquid’s polar and electron-rich environment.

Following substitution, the bromide ion produced in the reaction can exchange with the chloride anion in the ionic liquid layer, regenerating the active catalyst surface and allowing the catalytic cycle to continue. The nanocomposite framework provides structural robustness, prevents leaching of ionic liquid units into the solution, and exposes ample active sites through strong dispersion of the imidazolium phase on the GO@SiO_2_ scaffold.

The proposed mechanism highlights that the enhanced reactivity of aryl bromides in this system arises not from traditional transition-metal oxidative addition but from ionic-liquid-induced polarization and substrate activation at the heterogeneous catalyst interface. This dual functionality—combining surface basicity with ionic activation—accounts for the observed efficiency and recyclability of Bis-GO@SiO_2_@[(CH_2_)_3_Im][Cl] in the synthesis of 2-(phenylthio)benzo[d]thiazole.

**FIGURE 11:**
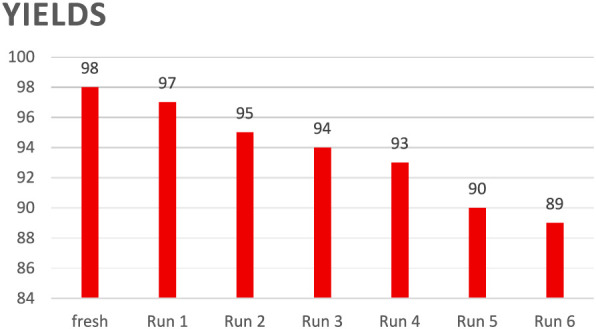
Reusability of Bis-GO@SiO_2_@[(CH_2_)_3_Im][Cl] nanocatalyst on the model reaction (product **4a**).

Figure 11 evaluates the reusability of the Bis-GO@SiO_2_@[(CH_2_)_3_Im][Cl] nanocatalyst in the model synthesis of 2-thioarybenzoxazole (product 4a). The catalyst was recovered and reused for six consecutive runs, and the corresponding isolated yields were recorded. The fresh catalyst afforded 98% yield, which was maintained at 97% after the first run. In subsequent cycles, the yield remained very high, showing only moderate attenuation: 95% for run 2, 94% for run 3, and 93% for run 4. A slight decrease was observed in later cycles, with yields of 90% and 89% for runs 5 and 6, respectively. Overall, the results indicate that the nanocatalyst preserves catalytic activity over multiple reuse cycles, with a total yield decline of only about 9% after six runs, demonstrating good stability and practical reusability under the reaction conditions employed.

**FIGURE 12:**
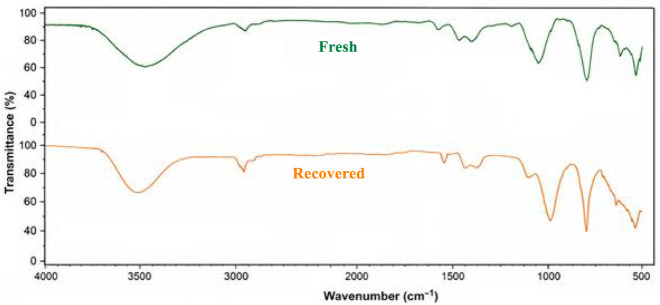
FT-IR spectrums of Bis-GO@SiO_2_@[(CH_2_)_3_Im][Cl] nanocatalyst fresh catalyst and recovered catalyst (after 6 times).

Figure 12 presents the FT-IR spectra of the fresh and recovered catalyst samples, illustrating the structural stability and potential chemical alterations after repeated catalytic use. The transmittance values, plotted against wavenumber in the range of 4,000–500 cm^-1^, provide insight into the surface functional groups and their retention following six catalytic cycles.

The spectrum of the fresh catalyst (green curve) displays well-defined absorption bands characteristic of its original chemical structure, confirming the presence of functional moieties responsible for catalytic activity. In contrast, the recovered catalyst (orange curve) exhibits slight variations in peak intensity and transmittance, particularly a general decrease across several wavenumber regions. This reduction implies minor structural changes or partial degradation of surface-active sites, possibly due to adsorption of reactants, partial loss of functional groups, or mild surface oxidation during the catalytic process.

Despite these subtle differences, the overall spectral profiles of the two samples remain largely comparable, suggesting that the fundamental structural framework of the catalyst remains intact even after multiple reuses. The preservation of characteristic absorption bands indicates that the catalyst maintains its core chemical integrity, confirming its reusability and durability under the applied reaction conditions. Therefore, the FT-IR analysis supports that the recovered catalyst retains most of its original functional characteristics, demonstrating good stability and resilience during repeated catalytic cycles.

**FIGURE 13:**
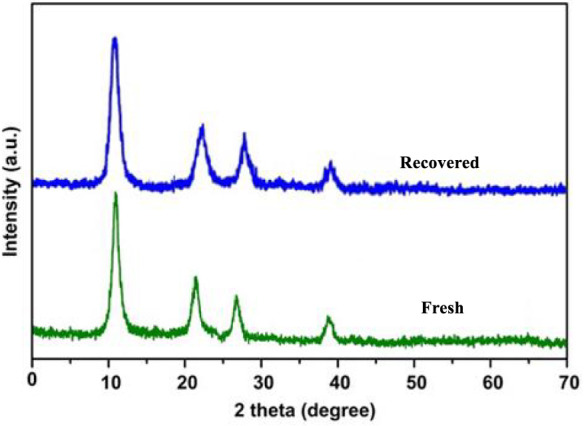
XRD patterns of Bis-GO@SiO_2_@[(CH_2_)_3_Im][Cl] nanocatalyst fresh catalyst and recovered catalyst (after 6 times).

Figure 13 presents the XRD patterns of the Bis-GO@SiO_2_@[(CH_2_)_3_Im][Cl] nanocatalyst, comparing a freshly prepared catalyst with a recovered sample after six reuse cycles. The patterns for both states exhibit several sharp reflections at low 2θ superimposed on an amorphous background, consistent with crystalline domains embedded within an amorphous silica matrix. In the fresh catalyst (green trace), the most pronounced reflections occur at approximately 9°–11°, accompanied by additional smaller peaks around 14°–16° and 20°–22°. The recovered catalyst (blue trace) displays a very similar set of reflections located at nearly the same 2θ positions, but with noticeably reduced peak intensities. There is no emergence of new peaks or detectable peak shifts in the recovered sample.

The retention of the characteristic peak positions indicates that the underlying crystalline framework of the nanocatalyst remains intact after six reuse cycles. The observed attenuation and mild broadening of the reflections in the recovered catalyst suggest a modest decrease in crystallinity, which may result from partial leaching, minor aggregation, or increased microstrain during repeated use. Despite these subtle changes, the overall diffraction pattern demonstrates structural stability of the Bis- Bis-GO@SiO_2_@[(CH_2_)_3_Im][Cl] nanocatalyst under the employed recycling conditions, supporting its robustness for repeated catalytic applications.

### Hot filtration test

**FIGURE 14:**
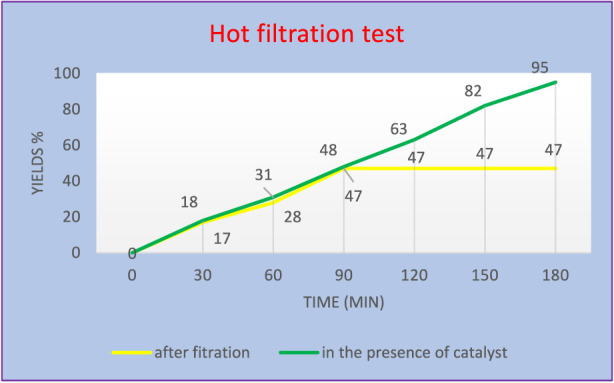
Leaching experiment of Bis-GO@SiO_2_@[(CH_2_)_3_Im][Cl] nanocatalyst on the model reaction (product **4a**).

Figure 14 summarizes the hot filtration (leaching) test conducted for the model reaction affording product 4a. The reaction profile recorded in the presence of the heterogeneous Bis-GO@SiO2@[(CH2)3Im][Cl] catalyst increases steadily from 0% at t = 0 to 18% at 30 min, 31% at 60 min, 48% at 90 min, 63% at 120 min, 82% at 150 min and 95% at 180 min. An aliquot of the reaction mixture was removed and the solid catalyst was separated by hot filtration at 90 min; the filtrate was allowed to continue under the same conditions. The filtrate shows parallel but arrested progress: 0% at t = 0, 17% at 30 min, 28% at 60 min and 47% at 90 min, after which the yield remains essentially constant at 47% through 180 min.

These data indicate that, once the solid catalyst is removed, no further meaningful conversion occurs in the filtered solution, whereas reactions maintained in the presence of the solid catalyst continue to proceed to high yield. The close correspondence of yields between the two experiments up to the filtration point (minor differences at 30 and 60 min) and the clear plateau of the filtrate after catalyst removal demonstrate that active catalytic species are not leached into the solution in quantities sufficient to sustain reaction progress. Consequently, the observed catalytic activity is attributable predominantly to the heterogeneous catalyst bound to the support rather than to homogeneous, soluble species.

For practical optimization of the reaction, these results imply that maintaining the solid Bis-GO@SiO_2_@[(CH_2_)_3_Im][Cl] catalyst in the reaction mixture is essential to achieve high conversion; under the present conditions the reaction reaches 95% yield after 180 min only when the catalyst remains present. The plateau at 47% in the absence of the solid catalyst indicates that reactions terminated by filtration before 90 min will not progress further in the filtrate, consistent with negligible leaching. These findings support the heterogeneous nature of the catalyst and validate strategies that rely on solid recovery and reuse without concern for significant loss of active species into solution.

Table 4 compares the efficiency of the present method for the synthesis of 2-thioarybenzoxazoles (product 4a as the model reaction) with several previously reported catalytic systems. In the reference studies, Fe_3_O_4_ nanoparticles were employed under K_2_CO_3_/DMF at 120 °C for 12 h, affording a 68% yield. A related approach using (IPr)CuCl under DMF and K_2_CO_3_ at 140 °C for 3 h gave an 81% yield, while CuI/Bipy in Na_2_CO_3_/DMF at 140 °C for 24 h produced 90%. Silver-based catalysis AgO_2_CCF_3_/Cu(OAc)_2_ in DMF at 120 °C for 12 h resulted in 76% yield. Additionally, CuFe_2_O_4_ nanoparticles activated with an additive set containing ChCl-urea and K_2_CO_3_ at 100 °C for 8 h delivered the highest yield among the literature entries (97%), though under conditions that differ from the present system. Under optimized conditions, Bis-GO@SiO_2_@[(CH_2_)_3_Im][Cl] catalyzed the reaction in DMF with K_2_CO_3_ at 50 °C for only 3 h, providing a 95% yield. Overall, the comparison shows that the proposed graphene oxide–based nanocatalyst achieves a high yield under substantially milder conditions and with competitive reaction time, outperforming most reported catalysts in terms of either temperature or duration, and approaching the best literature performance while using a lower temperature than the majority of previous systems.

**TABLE 4:** Comparison of the efficiency of this method with reported methods for the synthesis of 2-thioarylbenzoazoles (**product 4a**) as the model reaction.

Entry	Catalyst	Condition	Time (h)	Yield (%)	Ref.
1	Fe_3_O_4_ NPs	K_2_CO_3_, DMF, 120 °C	12 h	68%	18
2	(IPr)CuI	DMF, K_2_CO_3_, 140 °C	3 h	81%	21
3	CuI/Bipy	Na_2_CO_3_, DMF 140 °C	24 h	90%	19
4	AgO_2_CCF_3_/Cu(OAc)_2_	DMF, 120 °C	12 h	76%	20
5	CuFe_2_O_4_ NPs	ChCl-urea, KOAc, 100 °C	8 h	97%	17
6	**Bis-GO@SiO**_**2**_ **@[(CH**_**2**_ **)**_**3**_ **Im][Cl]**	**DMF, K**_**2**_ **CO**_**3**_ **, 50 °C**	**3 h**	**95%**	**This Method**

Bolded values are for greater emphasis and to indicate desirable conditions.

### Process mass intensity (PMI)

The environmental performance of the optimized reaction was assessed using the Process Mass Intensity (PMI) parameter, defined as the total mass of all input materials divided by the mass of the isolated product (Gavali et al., 2026). For the representative synthesis of 2-(phenylthio)benzoxazole (Table 1, Entry 8).

Based on the data provided in Table 1 of the manuscript for the optimized Entry 8, the PMI calculation is as follows.

- Reagents: benzoxazole (1.0 mmol, 119.12 mg), phenyl bromide (1.0 mmol, 157.01 mg), elemental sulfur (S_8_) (2.0 mmol, 64.13 mg), potassium carbonate (K_2_CO_3_) (3.0 mmol, 414.63 mg)
- Catalyst: Bis-GO@SiO_2_@[(CH_2_)_3_Im][Cl] (0.10 mg)
- Solvent: DMF (5.0 mL, density 0.944 g/mL = 4.72 g)
- Product (4a): Isolated yield of 95% of 2-(phenylthio)benzoxazole. Molar mass: 227.28 g/mol. Theoretical yield: 227.28 mg. Isolated yield: 215.92 mg.

PMI Calculation for Entry 8:

$$\text{Total Input Mass}=119.12\text{ mg }\left(\text{benzoxazole}\right)+157.01\text{ mg }\left(\text{phenyl bromide}\right)+64.13\text{ mg }\left(S_{8}\right)+414.63\text{ mg }\left(K_{2}{\text{CO}}_{3}\right)+0.10\text{ mg }\left(\text{catalyst}\right)+4.72\;g\;\left(\text{DMF}\right)=5475.00\text{ mg}$$

$$\text{PMI}=\frac{\text{Total}\;\text{Input}\;\text{Mass}}{\text{Isolated}\;\text{Product}\;\text{Mass}}=\frac{5475.00\;\text{mg}}{215.92\;\text{mg}}=25.36$$

This means that 25.36 kg of material are required to produce 1 kg of the 2-thioarylbenzoazole product under the optimized reaction conditions outlined in the manuscript.

The PMI value of ∼25.36 is a benchmark for a lab-scale process and aligns with the principles of green chemistry, particularly due to the low reaction temperature and high yield. This positions the Bis-GO@SiO_2_@[(CH_2_)_3_Im][Cl] catalyst as a highly efficient and sustainable option for the synthesis of 2-thioarylbenzoazoles. These results confirm that the present procedure offers both operational and environmental advantages, in agreement with previous recommendations for green synthetic evaluation. (DOI: 10.1007/s11164-026-05972-7).

### Experimental

All chemicals were purchased from Sigma-Aldrich and Merck. Reagents and solvents were obtained from Sigma-Aldrich, or Merck and were used as received without further purification. Infrared (IR) spectra of the samples were recorded as KBr disks using a NICOLET iS10 spectrometer (NICOLET Impact 410). The ^1^H NMR and ^13^C NMR spectra were obtained on a Bruker DRX-400 instrument operating at 400 MHz for ^1^H and 100 MHz for ^13^C.

### Preparation of Bis-GO@SiO_2_@[(CH_2_)_3_Im][Cl] nanocomposite

The Bis-GO@SiO_2_@[(CH_2_)_3_Im][Cl] nanocomposite was prepared via a three-step sequential procedure, beginning with the functionalization of graphene oxide (GO). In the first step, GO (0.50 g) was converted to GO–Cl by refluxing in a mixture of thionyl chloride (20 mL) and dimethylformamide (DMF, 60 mL) at 100 °C under a nitrogen atmosphere for 24 h. Excess SOCl_2_ was removed by distillation, and the resulting solid was washed with dimethylacetamide (DMAc), filtered, and dried under reduced pressure at 80 °C to ensure consistent functionalization. The GO–Cl intermediate (0.50 g) was then used to synthesize GO@SiO_2_@[(CH_2_)_3_Im][Cl]: GO–Cl was combined with 1H-imidazole (0.5 mmol) in dichloromethane (40 mL), the suspension was sonicated for 30 min to achieve a homogeneous dispersion, and the mixture was refluxed with vigorous stirring for 12 h. The product was isolated, washed with dichloromethane, and dried under vacuum to yield the Bis-GO@SiO_2_@[(CH_2_)_3_Im][Cl] material.

### General procedure for the preparation of 2-thioarylbenzoazoles

Bis-GO@SiO_2_@[(CH_2_)_3_Im][Cl] (10 mg) was added to a flask containing benzothiazole, benzimidazole, or benzoxazole (1.0 mmol), an aryl bromide (1.0 mmol), elemental sulfur (S8, 1.5 mmol) and K_2_CO_3_ (3.0 mmol) in DMF (5.0 mL). The reaction mixture was stirred at 50 °C under an N_2_ atmosphere for 3 h and its progress was monitored by TLC (hexane/ethyl acetate as eluent). Upon completion, the mixture was allowed to cool to room temperature for 10 min and insoluble material was removed by filtration. The filter cake was suspended in 3 mL of hot ethanol, and the catalyst was recovered by centrifugation, washed with ethanol and dried in an oven for reuse. The combined filtrates were poured into ice-cold water to induce precipitation of the product; the precipitate was collected by filtration, washed with ice-cold water, dried, and finally purified by washing with ethyl acetate.

## Conclusion

In summary, the Bis-GO@SiO_2_@[(CH_2_)_3_Im][Cl] nanocatalyst developed in this study represents a significant advancement in the sustainable synthesis of 2-thioarylbenzoazoles. By integrating bis-functionalized graphene oxide with a silica interlayer and an immobilized imidazolium ionic liquid, we have created a robust, well-dispersed hybrid material that efficiently promotes the multicomponent reaction of benzimidazoles, benzoxazoles, or benzothiazoles with aryl halides and elemental sulfur under notably mild conditions. The protocol operates effectively at 50 °C in the presence of K_2_CO_3_ in DMF, delivering a wide array of structurally diverse 2-thioarylbenzoazole derivatives in good to excellent yields.

The catalyst exhibits several key advantages, including high activity, straightforward recovery by simple filtration, and excellent recyclability over at least five consecutive runs without substantial loss of performance. Its design leverages the synergistic effects of the high-surface-area support and the ionic liquid phase, resulting in enhanced stability and accessibility of active sites. This approach overcomes many limitations of conventional methods, such as harsh reaction conditions, stoichiometric reagent use, and difficult catalyst separation, thereby offering a more environmentally responsible and operationally simple route to these valuable heterocyclic compounds.

Overall, the present work provides a practical and green platform for the construction of sulfur-containing benzoazoles with potential applications in medicinal chemistry and advanced materials. Future investigations may focus on expanding the substrate scope, exploring continuous-flow applications, and further elucidating the precise role of the ionic liquid layer in the catalytic cycle. This contribution is expected to stimulate further developments in the design of multifunctional nanocatalysts for sustainable organic synthesis.

## Novelty of the work

This study presents the first application of a bis-functionalized graphene oxide–silica–ionic liquid hybrid nanocatalyst, Bis-GO@SiO_2_@[(CH_2_)_3_Im][Cl], for the direct multicomponent synthesis of 2-thioarylbenzoazoles. The developed protocol achieves the regioselective multicomponent reaction of benzimidazoles, benzoxazoles, or benzothiazoles with aryl halides and elemental sulfur (S_8_) through a three-component A3-type reaction under exceptionally mild conditions—50 °C in DMF using K_2_CO_3_ as base—without requiring expensive ligands, transition-metal co-catalysts, or harsh reaction temperatures commonly reported in previous methods.

The novelty lies in several aspects: the rational design of a stable, recyclable hybrid nanocatalyst that combines the high surface area and dispersibility of graphene oxide with the protective silica interlayer and the activating effect of the immobilized imidazolium ionic liquid; the successful integration of elemental sulfur as a low-cost, readily available sulfur source in this transformation; and the broad substrate compatibility that allows access to a diverse library of 2-thioarylbenzoazole derivatives in good to excellent yields. Furthermore, the catalyst’s straightforward recovery by filtration and consistent performance over multiple cycles address key sustainability challenges associated with traditional homogeneous or non-recyclable catalytic systems.

By offering an operationally simple, metal-free, and environmentally more benign route to these medicinally and materially relevant heterocycles, the present approach introduces a new and practical strategy that advances the field of sustainable nanocatalysis for C–S bond-forming multicomponent reactions.

## Data Availability

The original contributions presented in the study are included in the article/Supplementary Material, further inquiries can be directed to the corresponding author.
