# Supplementary material for: Bis-GO@SiO2@[(CH2)3Im][Cl] nanocatalyst: a mild, reusable, and sustainable multicomponent platform for the synthesis of 2-thioarylbenzoazoles
Source: Front Chem. 2026 Jun 24;14:1856254. doi: 10.3389/fchem.2026.1856254 (PMC13341079; doi:10.3389/fchem.2026.1856254)
Supplement: Supplementary file 1 [file DataSheet1.pdf]

Bis-GO@SiO_2@[$(CH_2)_3$Im][Cl] Nanocatalyst: A Mild, Reusable, and Sustainable Multicomponent Platform for the Synthesis of 2-Thioarylbenzoazoles

Yongshan Fu, [1]

1,

School of Carbon Peak and Carbon Neutrality Technology, Sichuan Technology & Business College, Chengdu, Sichuan, 611830, China.

fuyongshan7@gmail.com

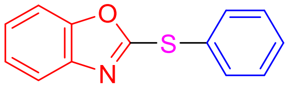

2-(phenylthio)benzo[d]oxazole

(oil)

^{1}H NMR (400 MHz, DMSO) δ 7.66 (d, *J* = 7.7 Hz, 2H), 7.52-7.47 (m, 3H), 7.41 (d, *J* = 8.0 Hz, 2H), 7.34 (t, *J* = 7.6 Hz, 2H); ^{13}C NMR (100 MHz, DMSO) δ 152.3, 150.9, 141.3, 136.4, 129.8, 129.6, 125.7, 124.3, 123.1, 119.5, 110.8 ppm.

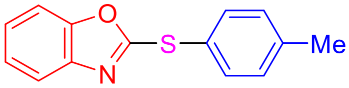

2-(p-tolylthio)benzo[d]oxazole

(oil)

^{1}H NMR (400 MHz, DMSO) δ 7.67 (d, *J* = 7.4 Hz, 2H), 7.49 (d, *J* = 8.0 Hz, 2H), 7.39-7.34 (m, 2H), 7.27 (d, *J* = 8.0 Hz, 2H), 2.34 (s, 3H); ^{13}C NMR (100 MHz, DMSO) δ 152.9, 151.7, 141.3, 138.2, 133.4, 129.0, 124.6, 123.2, 110.7, 21.0 ppm.

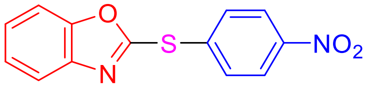

2-((4-nitrophenyl)thio)benzo[d]oxazole

(M.P: 93-95ºC)

^{1}H NMR (400 MHz, DMSO) δ 7.98 (d, *J* = 8.6 Hz, 2H), 7.66 (d, *J* = 7.7 Hz, 4H), 7.36 (t, *J* = 7.6 Hz, 2H); ^{13}C NMR (100 MHz, DMSO) δ 152.7, 151.4, 144.1, 142.3, 141.2, 129.0, 125.6, 124.1, 123.2, 119.6, 110.4 ppm.

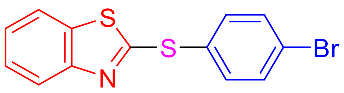

2-((4-bromophenyl)thio)benzo[d]thiazole

(M.P: 50-52 °C)

^{1}H NMR (400 MHz, DMSO) δ 8.22 (d, *J* = 8.0 Hz, 1H), 8.09 (d, *J* = 7.7 Hz, 1H), 7.75 (d, *J* = 7.4 Hz, 2H), 7.56-7.47 (m, 4H); ^{13}C NMR (100 MHz, DMSO) δ 164.3, 153.2, 135.1, 132.4, 128.0, 125.2, 124.7, 121.8, 119.4 ppm.

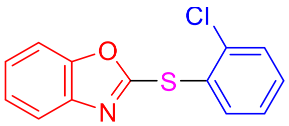

2-((2-chlorophenyl)thio)benzo[d]oxazole

(M.P: 46-48 °C)

^{1}H NMR (400 MHz, DMSO) δ 7.81 (d, *J* = 7.7 Hz, 1H), 7.66 (d, *J* = 7.6 Hz, 2H), 7.39-7.32 (m, 2H), 7.31 (d, *J* = 7.4 Hz, 1H), 7.14 (t, *J* = 8.0 Hz, 1H), 7.07 (t, *J* = 7.8 Hz, 1H); ^{13}C NMR (100 MHz, DMSO) δ 153.1, 152.7, 141.3, 133.5, 131.0, 130.1, 127.8, 126.3, 124.2, 123.0, 119.7, 110.8 ppm.

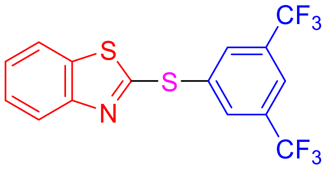

2-((3,5-bis(trifluoromethyl)phenyl)thio)benzo[d]thiazole

(oil)

^{1}H NMR (400 MHz, DMSO) δ 8.21 (d, *J* = 7.8 Hz, 1H), 8.09 (d, *J* = 8.0 Hz, 1H), 7.68 (s, 1H), 7.57-7.47 (m, 2H), 7.40 (s, 2H); ^{13}C NMR (100 MHz, DMSO) δ 164.3, 153.1, 137.0, 135.4, 131.2, 127.0, 126.5, 125.1, 124.3, 121.0, 119.2 ppm.

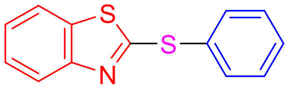

2-(phenylthio)benzo[d]thiazole

(M.P: 33-35 °C)

^{1}H NMR (400 MHz, DMSO) δ 8.19 (d, *J* = 7.6 Hz, 1H), 8.08 (d, *J* = 8.0 Hz, 1H), 7.57-7.50 (m, 2H), 7.47 (t, *J* = 7.9 Hz, 3H), 7.37 (d, *J* = 8.0 Hz, 2H); ^{13}C NMR (100 MHz, DMSO) δ 164.3, 153.1, 136.7, 135.4, 129.1, 125.6, 124.3, 121.8 ppm.

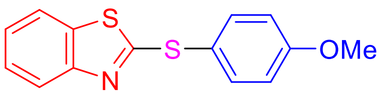

2-((4-methoxyphenyl)thio)benzo[d]thiazole

(M.P: 55-57 ºC)

^{1}H NMR (400 MHz, DMSO) δ 8.19 (d, *J* = 6.9 Hz, 2H), 8.07 (d, *J* = 7.8 Hz, 1H), 7.57-7.49 (m, 2H), 7.35 (d, *J* = 7.7 Hz, 2H), 7.21 (d, *J* = 8.6 Hz, 2H), 3.82 (s, 3H); ^{13}C NMR (100 MHz, DMSO) δ 164.1, 157.6, 153.1, 135.4, 129.8, 126.5, 125.1, 124.0, 121.3, 114.6, 55.4 ppm.

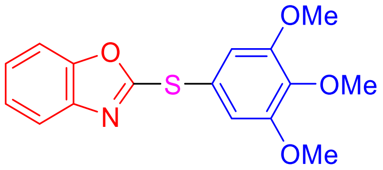

2-((3,4,5-trimethoxyphenyl)thio)benzo[d]oxazole

(M.P: 129-131 ºC)

^{1}H NMR (400 MHz, DMSO) δ 7.68 (d, *J* = 9.0 Hz, 2H), 7.37 (t, *J* = 7.6 Hz, 2H), 6.21 (s, 2H), 3.86 (s, 6H), 3.69 (s, 3H); ^{13}C NMR (100 MHz, DMSO) δ 153.2, 152.9, 151.3, 141.6, 136.7, 130.2, 125.3, 124.1, 119.8, 110.5, 107.4, 60.7, 56.1 ppm.

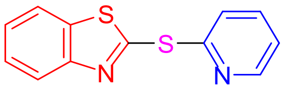

2-(pyridin-2-ylthio)benzo[d]thiazole

(M.P: 66-68 ºC)

^{1}H NMR (400 MHz, DMSO) δ 8.42 (d, *J* = 8.2 Hz, 1H), 8.19 (d, *J* = 7.62 Hz, 1H), 8.06 (d, *J* = 7.76 Hz, 1H), 7.73 (t, *J* = 8.0 Hz, 1H), 7.55-7.51 (m, 2H), 7.31-7.26 (m, 2H); ^{13}C NMR (100 MHz, DMSO) δ 164.5, 153.1, 152.4, 149.1, 137.5, 135.6, 125.7, 124.3, 122.6, 120.9 ppm.

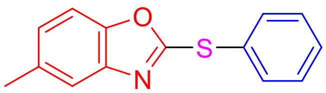

5-methyl-2-(phenylthio)benzo[d]oxazole

(M.P: 48-50 ºC)

^{1}H NMR (400 MHz, DMSO) δ 7.71 (d, *J* = 7.4 Hz, 1H), 7.63 (s, 1H), 7.49-7.42 (m, 3H), 7.36 (d, *J* = 7.7 Hz, 2H), 7.15 (d, *J* = 8.0 Hz, 1H), 2.47 (s, 3H); ^{13}C NMR (100 MHz, DMSO) δ 152.3, 148.0, 141.4, 136.2, 133.1, 130.8, 129.1, 125.4, 119.0, 109.6, 21.3 ppm.

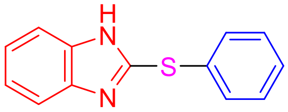

2-(phenylthio)-1H-benzo[d]imidazole

(M.P: 202-204 ºC)

^{1}H NMR (400 MHz, DMSO) δ 13.44 (s, 1H), 7.66 (d, *J* = 6.9 Hz, 2H), 7.52-7.47 (m,3H), 7.39 (d, *J* = 7.7 Hz, 2H), 7.12 (t, *J* = 7.8 Hz, 2H); ^{13}C NMR (100 MHz, DMSO) δ 141.2, 138.7, 132.0, 130.8, 129.1, 126.3, 123.4, 115.2 ppm.

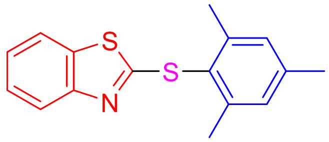

2-(mesitylthio)benzo[d]thiazole

(oil)

^{1}H NMR (400 MHz, DMSO) δ 8.18 (d, *J* = 7.9 Hz, 1H), 8.06 (d, *J* = 7.7 Hz, 1H), 7.56-7.50 (m,2H), 6.82 (s, 2H), 2.37 (s, 6H), 2.19 (s, 3H); ^{13}C NMR (100 MHz, DMSO) δ 164.3, 153.1, 141.5, 135.2, 129.7, 128.0, 125.4, 124.3, 121.1, 21.9, 21.5 ppm.

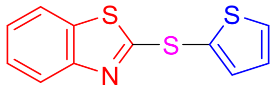

2-(thiophen-2-ylthio)benzo[d]thiazole

(oil)

^{1}H NMR (400 MHz, DMSO) δ 8.20 (d, *J* = 8.4 Hz, 1H), 8.07 (d, *J* = 7.7 Hz, 1H), 7.58-7.52 (m,2H), 7.48 (d, *J* = 6.9 Hz, 1H), 7.12 (d, *J* = 7.8 Hz, 1H), 6.73 (t, *J* = 8.0 Hz, 1H); ^{13}C NMR (100 MHz, DMSO) δ 164.7, 153.1, 135.2, 127.0, 126.5, 125.1, 124.6, 123.0, 121.8 ppm.

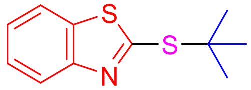

2-(tert-butylthio)benzo[d]thiazole

(oil)

^{1}H NMR (400 MHz, DMSO) δ 8.00 (d, *J* = 7.6 Hz, 1H), 7.83 (d, *J* = 8.2 Hz, 1H), 7.56-7.48 (m, 2H), 1.37 (s, 9H); ^{13}C NMR (100 MHz, DMSO) δ 164.2, 153.1, 135.6, 125.7, 124.3, 121.4, 49.8, 30.5 ppm.

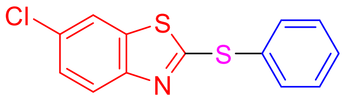

6-chloro-2-(phenylthio)benzo[d]thiazole

(M.P: 69-71 ºC)

^{1}H NMR (400 MHz, DMSO) δ 8.12 (s, 1H), 7.62 (d, J = 8.0 Hz, 1H), 7.55 (d, J = 7.6 Hz, 1H), 7.47-7.42 (m, 3H), 7.37 (d, J = 7.8 Hz, 2H); ^{13}C NMR (100 MHz, DMSO) δ 164.2, 151.6, 136.1, 130.8, 129.0, 126.2, 123.4, 121.1 ppm.

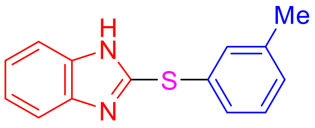

2-(m-tolylthio)-1H-benzo[d]imidazole

(oil)

^{1}H NMR (400 MHz, DMSO) δ 13.44 (s, 1H), 7.66 (d, J = 9.0 Hz, 1H), 7.27 (d, J = 7.67 Hz, 1H), 7.21-7.12 (m, 4H), 6.85 (d, J = 8.0 Hz, 1H), 2.38 (s, 3H); ^{13}C NMR (100 MHz, DMSO) δ 141.2, 140.8, 138.7, 136.2, 130.1, 129.3, 126.7, 125.0, 123.8, 115.4, 21.9 ppm.

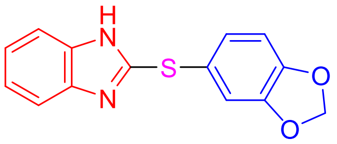

2-(benzo[d][1,3]dioxol-5-ylthio)-1H-benzo[d]imidazole

(M.P: 183-185 ^{o}C)

^{1}H NMR (400 MHz, DMSO) δ 13.43 (s, 1H), 7.65 (d, J = 8.0Hz, 2H), 7.22-7.12 (m, 2H), 7.06 (d, J = 7.6 Hz, 1H), 6.83 (d, J = 7.7 Hz, 1H), 6.53 (s, 1H), 6.02 (s, 1H); ^{13}C NMR (100 MHz, DMSO) δ 149.0, 145.6, 141.2, 138.1, 130.2, 123.7, 118.6, 115.4, 111.3, 101.8 ppm.

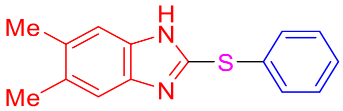

5,6-dimethyl-2-(phenylthio)-1H-benzo[d]imidazole

(M.P: 167-169 ^{o}C)

^{1}H NMR (400 MHz, DMSO) δ 13.23 (s, 1H), 7.48-7.41 (m ,3H), 7.38 (d, J = 8.0 Hz, 2H), 7.35 (s, 2H), 2.46 (s, 6H); ^{13}C NMR (100 MHz, DMSO) δ 141.2, 136.0, 132.6, 131.4, 130.2, 129.1, 125.7, 115.0, 18.6 ppm.

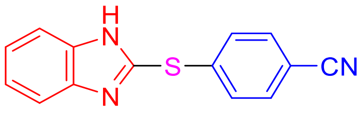

4-((1H-benzo[d]imidazol-2-yl)thio)benzonitrile

(M.P: 178-180 ^{o}C)

^{1}H NMR (400 MHz, DMSO) δ 13.45 (s, 1H), 7.66 (d, *J* = 7.9 Hz, 2H), 7.53 (d, *J* = 7.6 Hz, 2H), 7.43 (d, *J* = 8.0 Hz, 2H), 7.15-7.10 (m, 2H); ^{13}C NMR (100 MHz, DMSO) δ 141.3, 138.0, 137.1, 132.4, 130.7, 123.5, 118.4, 115.2 ppm.

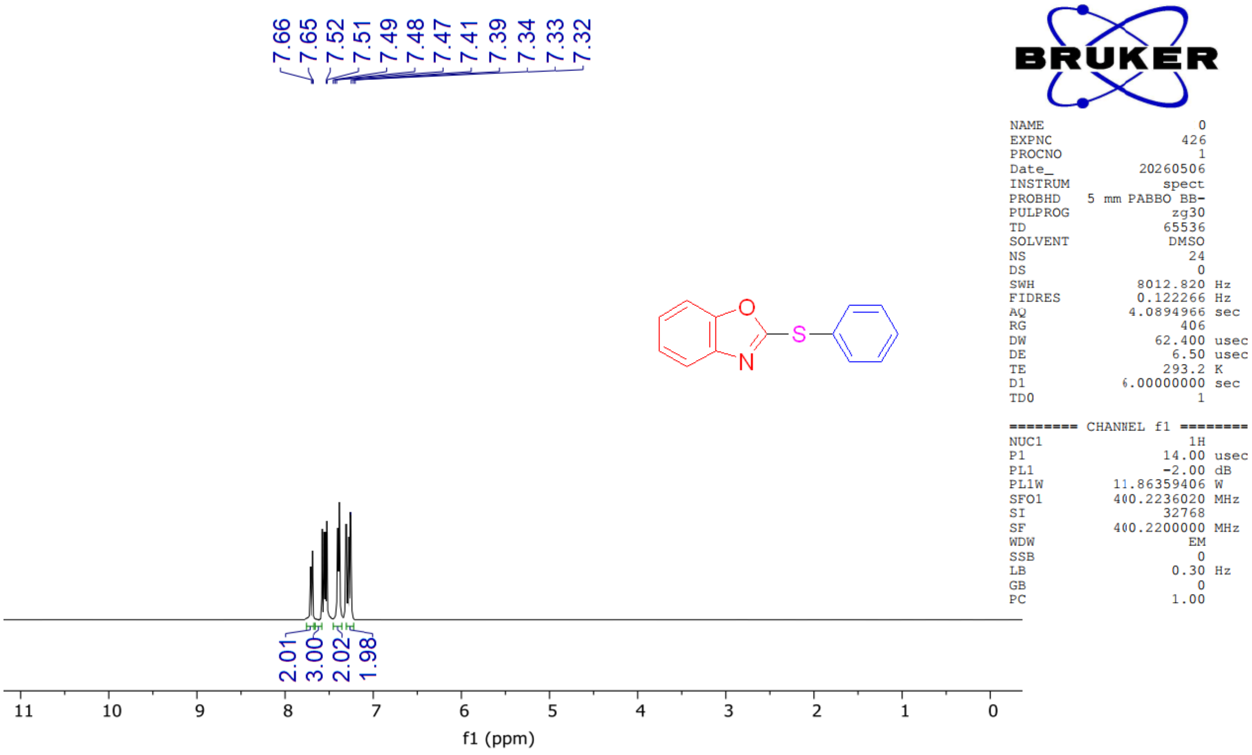

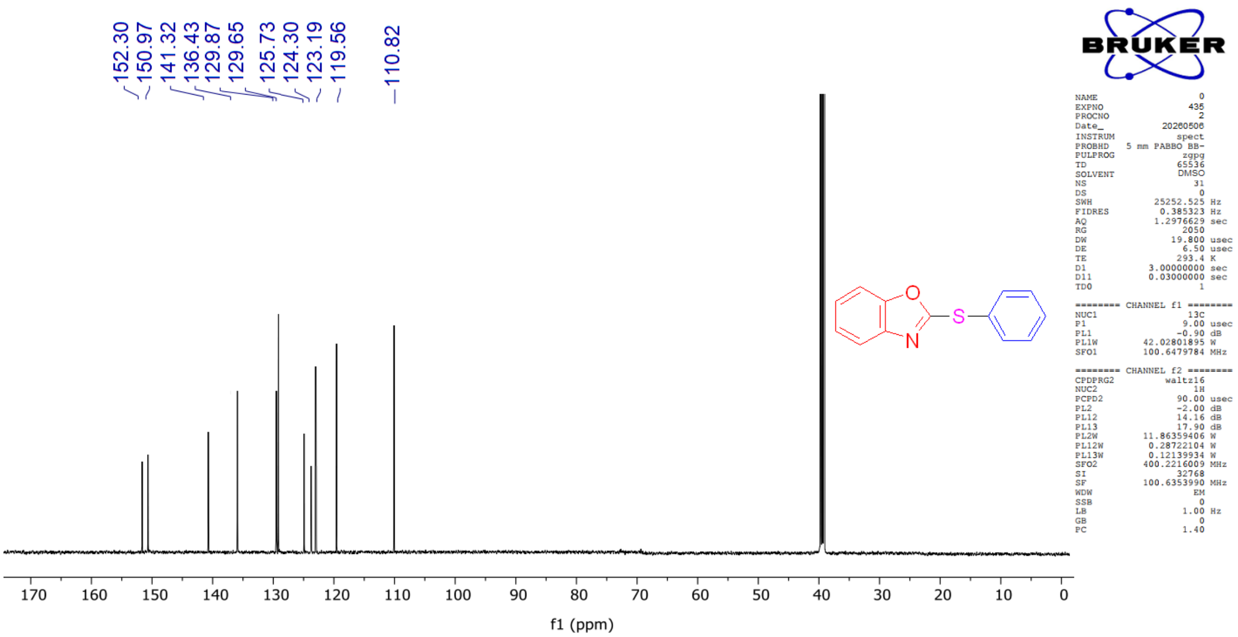
152.30
150.97
141.32
136.43
129.87
129.65
125.73
124.30
123.19
119.56
110.82
BRUKER
NAME 0
EXPNO 435
PROCNO 2
Date_ 20260506
INSTRUM spect
PROBHD 5 mm PABBO BB-
PULPROG zgpg
TD 65536
SOLVENT DMSO
NS 31
DS 0
SWH 25252.525 Hz
FIDRES 0.385323 Hz
AQ 1.2976629 sec
RG 2050
DW 19.800 usec
DE 6.50 usec
TE 293.4 K
D1 3.00000000 sec
D11 0.03000000 sec
TD0 1
======== CHANNEL f1 ========
NUC1 13C
P1 9.00 usec
PL1 -0.90 dB
PL1W 42.02801895 W
SFO1 100.6479784 MHz
======== CHANNEL f2 ========
CPDPRG2 waltz16
NUC2 1H
PCPD2 90.00 usec
PL2 -2.00 dB
PL12 14.16 dB
PL13 17.90 dB
PL2W 11.86359406 W
PL12W 0.28722104 W
PL13W 0.12139934 W
SFO2 400.2216009 MHz
SI 32768
SF 100.6353990 MHz
WDW EM
SSB 0
LB 1.00 Hz
GB 0
PC 1.40
O
N
S
170 160 150 140 130 120 110 100 90 80 70 60 50 40 30 20 10 0
f1 (ppm)

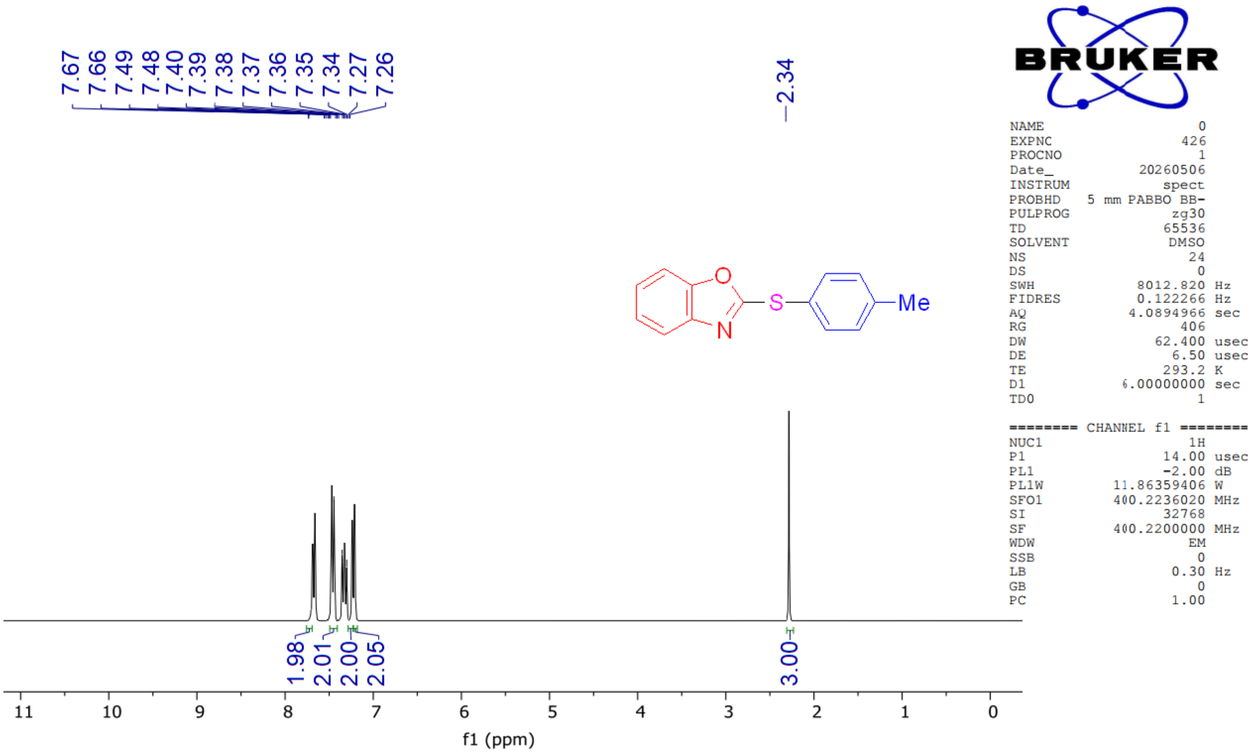
7.67
7.66
7.49
7.48
7.40
7.39
7.38
7.37
7.36
7.35
7.34
7.27
7.26
2.34
BRUKER
NAME 0
EXPNO 426
PROCNO 1
Date_ 20260506
INSTRUM spect
PROBHD 5 mm PABBO BB-
PULPROG zg30
TD 65536
SOLVENT DMSO
NS 24
DS 0
SWH 8012.820 Hz
FIDRES 0.122266 Hz
AQ 4.0894966 sec
RG 406
DW 62.400 usec
DE 6.50 usec
TE 293.2 K
D1 6.00000000 sec
TD0 1
======== CHANNEL f1 ========
NUC1 1H
P1 14.00 usec
PL1 -2.00 dB
PL1W 11.86359406 W
SFO1 400.2236020 MHz
SI 32768
SF 400.2200000 MHz
WDW EM
SSB 0
LB 0.30 Hz
GB 0
PC 1.00
O
N
S
Me
1.98
2.01
2.00
2.05
3.00
11 10 9 8 7 6 5 4 3 2 1 0
f1 (ppm)

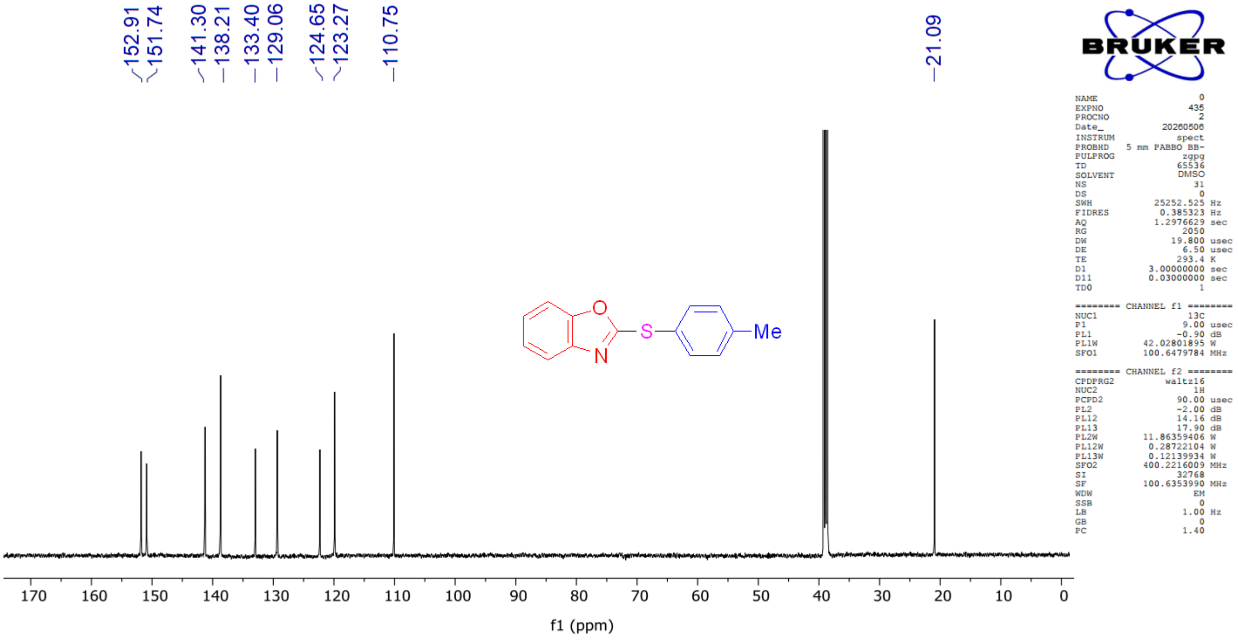
152.91
151.74
141.30
138.21
133.40
129.06
124.65
123.27
110.75
21.09
S
Me
O
N
BRUKER
NAME 0
EXPNO 435
PROCNO 2
Date_ 20260506
INSTRUM spect
PROBHD 5 mm PABBO BB-
PULPROG zgpg
TD 65536
SOLVENT DMSO
NS 31
DS 0
SWH 25252.525 Hz
FIDRES 0.385323 Hz
AQ 1.2976629 sec
RG 2050
DW 19.800 usec
DE 6.50 usec
TE 293.4 K
D1 3.00000000 sec
D11 0.03000000 sec
TD0 1
======= CHANNEL f1 =======
NUC1 13C
P1 9.00 usec
PL1 -0.90 dB
PL1W 42.02801895 W
SFO1 100.6479784 MHz
======= CHANNEL f2 =======
CPDPRG2 waltz16
NUC2 1H
PCPD2 90.00 usec
PL2 -2.00 dB
PL12 14.16 dB
PL13 17.90 dB
PL2W 11.86359406 W
PL12W 0.28722104 W
PL13W 0.12139934 W
SFO2 400.2216009 MHz
SI 32768
SF 100.6353990 MHz
WDW EM
SSB 0
LB 1.00 Hz
GB 0
PC 1.40
170 160 150 140 130 120 110 100 90 80 70 60 50 40 30 20 10 0
f1 (ppm)

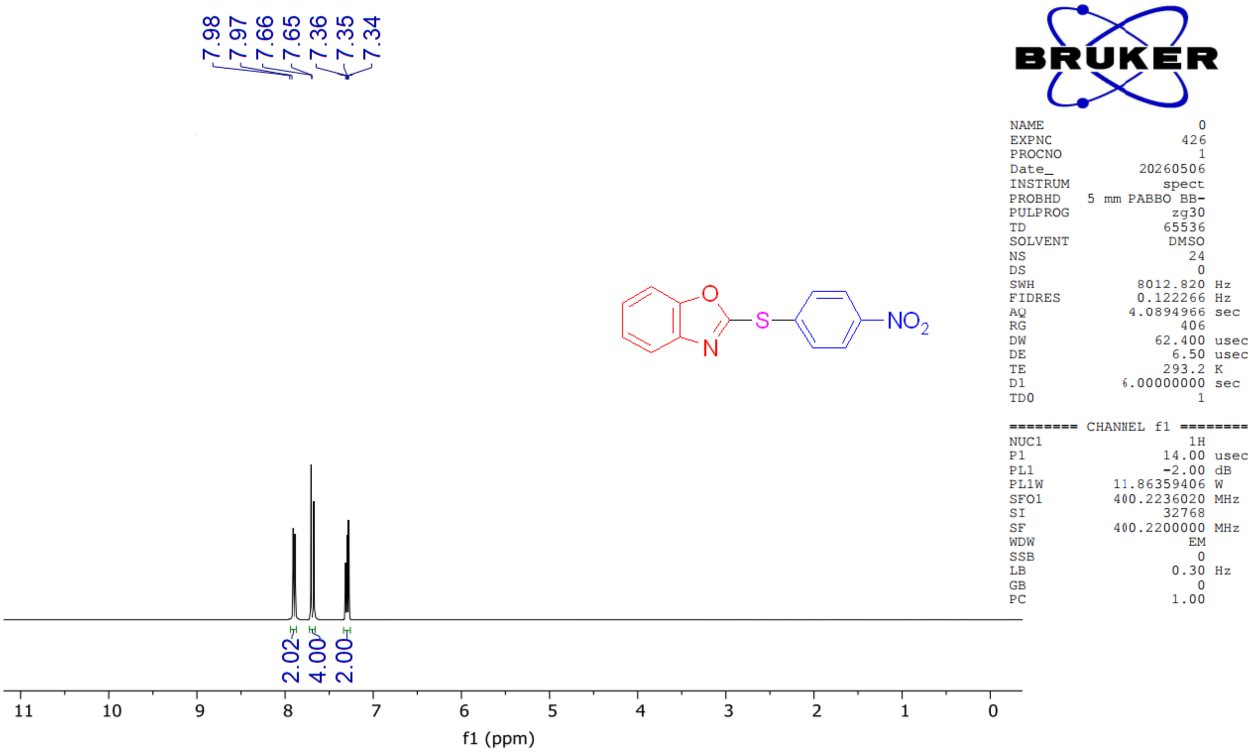
7.98
7.97
7.66
7.65
7.36
7.35
7.34
O
N
S
NO2
BRUKER
NAME 0
EXPNO 426
PROCNO 1
Date_ 20260506
INSTRUM spect
PROBHD 5 mm PABBO BB-
PULPROG zg30
TD 65536
SOLVENT DMSO
NS 24
DS 0
SWH 8012.820 Hz
FIDRES 0.122266 Hz
AQ 4.0894966 sec
RG 406
DW 62.400 usec
DE 6.50 usec
TE 293.2 K
D1 6.00000000 sec
TD0 1
======== CHANNEL f1 ========
NUC1 1H
P1 14.00 usec
PL1 -2.00 dB
PL1W 11.86359406 W
SFO1 400.2236020 MHz
SI 32768
SF 400.2200000 MHz
WDW EM
SSB 0
LB 0.30 Hz
GB 0
PC 1.00
2.02
4.00
2.00
11 10 9 8 7 6 5 4 3 2 1 0
f1 (ppm)

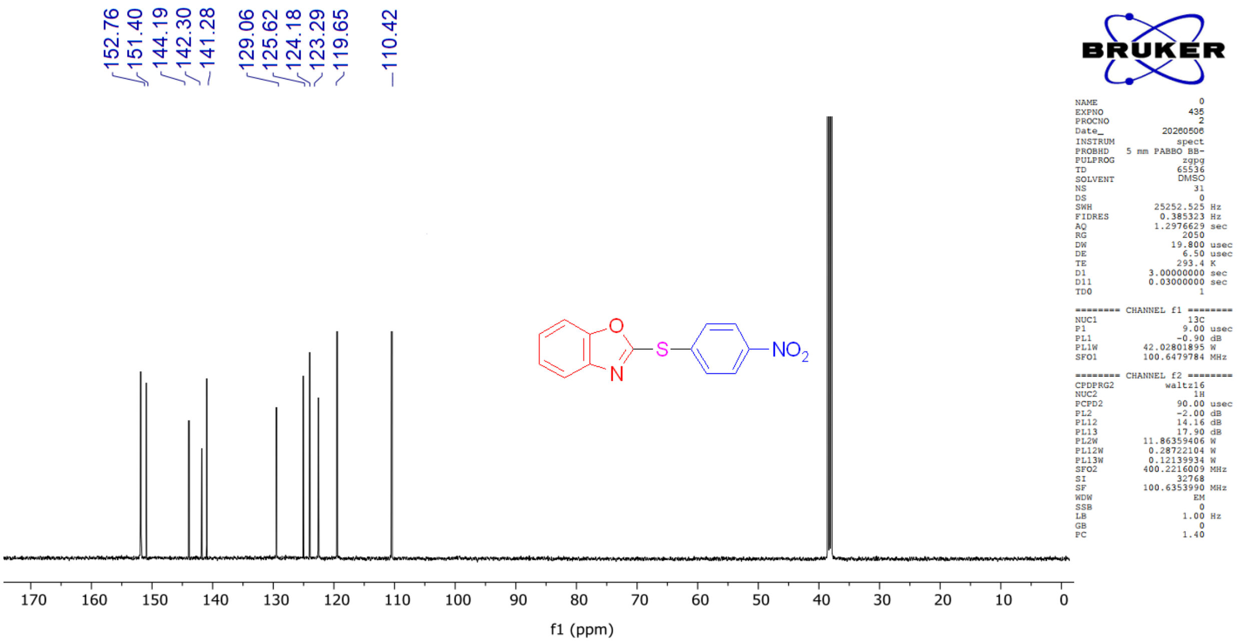
152.76
151.40
144.19
142.30
141.28
129.06
125.62
124.18
123.29
119.65
110.42
O
N
S
NO2
BRUKER
NAME 0
EXPNO 435
PROCNO 2
Date_ 20260506
INSTRUM spect
PROBHD 5 mm PABBO BB-
PULPROG zgpg
TD 65536
SOLVENT DMSO
NS 31
DS 0
SWH 25252.525 Hz
FIDRES 0.385323 Hz
AQ 1.2976629 sec
RG 2050
DW 19.800 usec
DE 6.50 usec
TE 293.4 K
D1 3.00000000 sec
D11 0.03000000 sec
TD0 1
======== CHANNEL f1 ========
NUC1 13C
P1 9.00 usec
PL1 -0.90 dB
PL1W 42.02801895 W
SFO1 100.6479784 MHz
======== CHANNEL f2 ========
CPDPRG2 waltz16
NUC2 1H
PCPD2 90.00 usec
PL2 -2.00 dB
PL12 14.16 dB
PL13 17.90 dB
PL2W 11.86359406 W
PL12W 0.28722104 W
PL13W 0.12139934 W
SFO2 400.2216009 MHz
SI 32768
SF 100.6353990 MHz
WDW EM
SSB 0
LB 1.00 Hz
GB 0
PC 1.40
170 160 150 140 130 120 110 100 90 80 70 60 50 40 30 20 10 0
f1 (ppm)

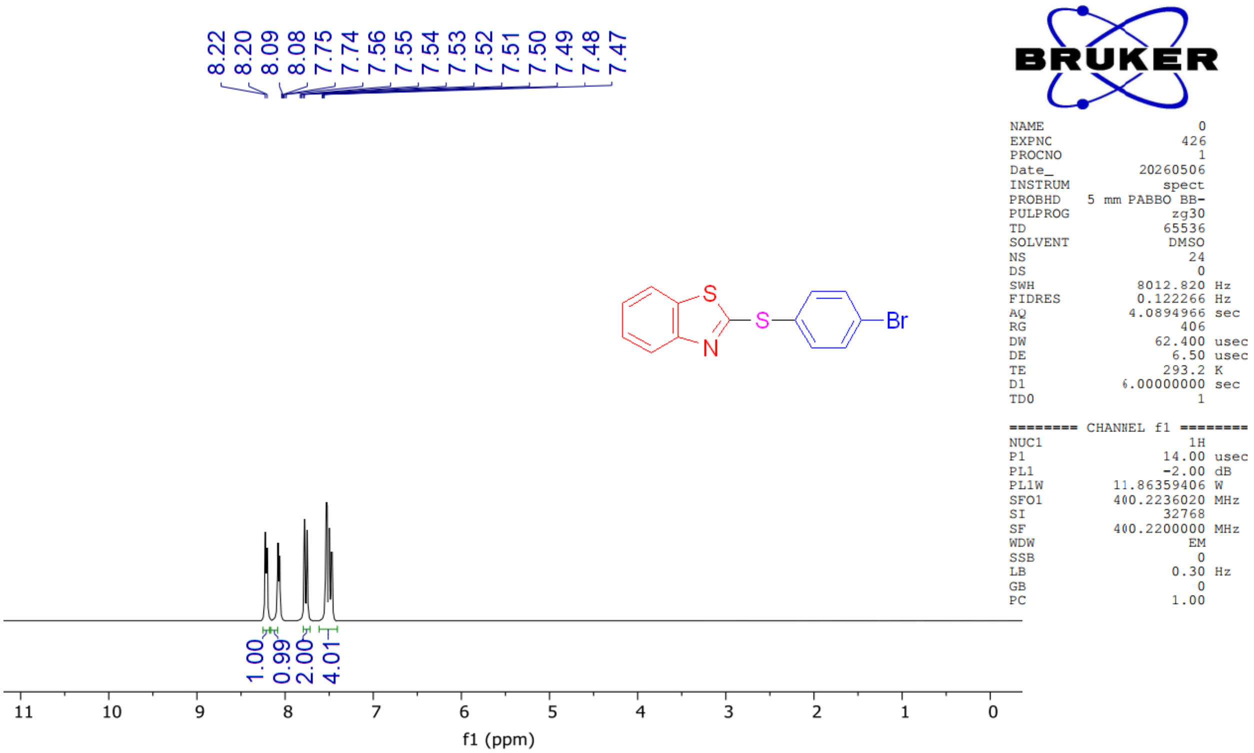
8.22
8.20
8.09
8.08
7.75
7.74
7.56
7.55
7.54
7.53
7.52
7.51
7.50
7.49
7.48
7.47
S
N
S
Br
BRUKER
NAME 0
EXPNO 426
PROCNO 1
Date_ 20260506
INSTRUM spect
PROBHD 5 mm PABBO BB-
PULPROG zg30
TD 65536
SOLVENT DMSO
NS 24
DS 0
SWH 8012.820 Hz
FIDRES 0.122266 Hz
AQ 4.0894966 sec
RG 406
DW 62.400 usec
DE 6.50 usec
TE 293.2 K
D1 6.00000000 sec
TD0 1
======== CHANNEL f1 ========
NUC1 1H
P1 14.00 usec
PL1 -2.00 dB
PL1W 11.86359406 W
SFO1 400.2236020 MHz
SI 32768
SF 400.2200000 MHz
WDW EM
SSB 0
LB 0.30 Hz
GB 0
PC 1.00
1.00
0.99
2.00
4.01
11 10 9 8 7 6 5 4 3 2 1 0
f1 (ppm)

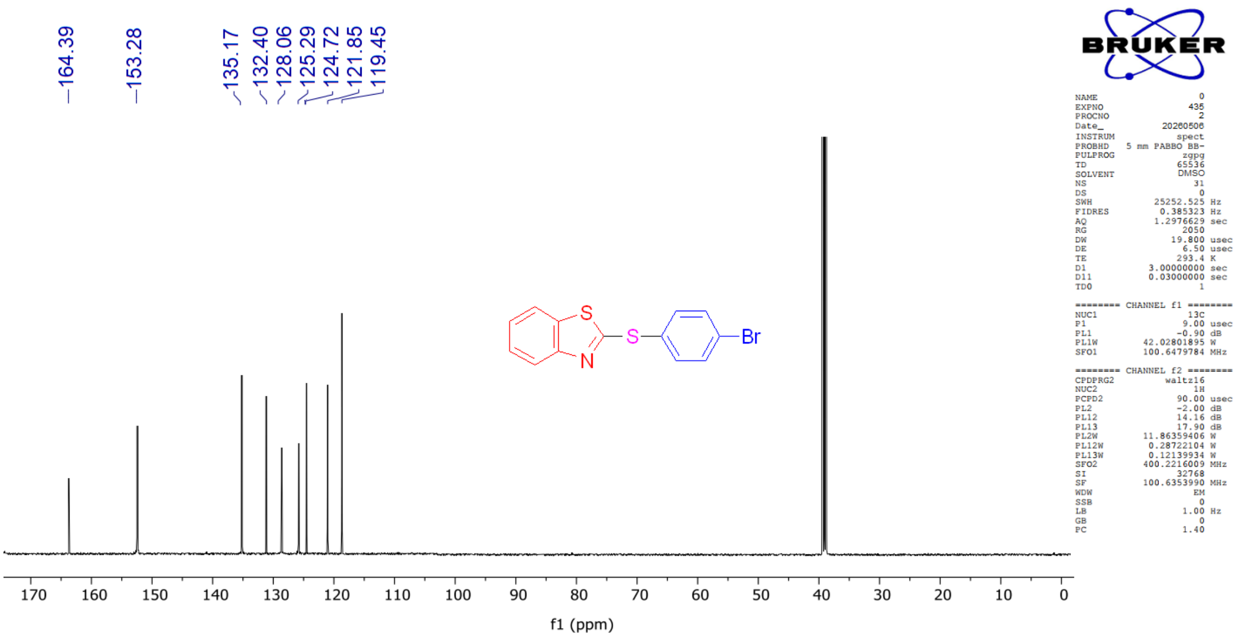

164.39
153.28
135.17
132.40
128.06
125.29
124.72
121.85
119.45
S
N
S
Br
BRUKER
NAME 0
EXPNO 435
PROCNO 2
Date_ 20260506
INSTRUM spect
PROBHD 5 mm PABBO BB-
PULPROG zgpg
TD 65536
SOLVENT DMSO
NS 31
DS 0
SWH 25252.525 Hz
FIDRES 0.385323 Hz
AQ 1.2976629 sec
RG 2050
DW 19.800 usec
DE 6.50 usec
TE 293.4 K
D1 3.00000000 sec
D11 0.03000000 sec
TD0 1
======== CHANNEL f1 ========
NUC1 13C
P1 9.00 usec
PL1 -0.90 dB
PL1W 42.02801895 W
SFO1 100.6479784 MHz
======== CHANNEL f2 ========
CPDPRG2 waltz16
NUC2 1H
PCPD2 90.00 usec
PL2 -2.00 dB
PL12 14.16 dB
PL13 17.90 dB
PL2W 11.86359406 W
PL12W 0.28722104 W
PL13W 0.12139934 W
SFO2 400.2216009 MHz
SI 32768
SF 100.6353990 MHz
WDW EM
SSB 0
LB 1.00 Hz
GB 0
PC 1.40
170 160 150 140 130 120 110 100 90 80 70 60 50 40 30 20 10 0
f1 (ppm)

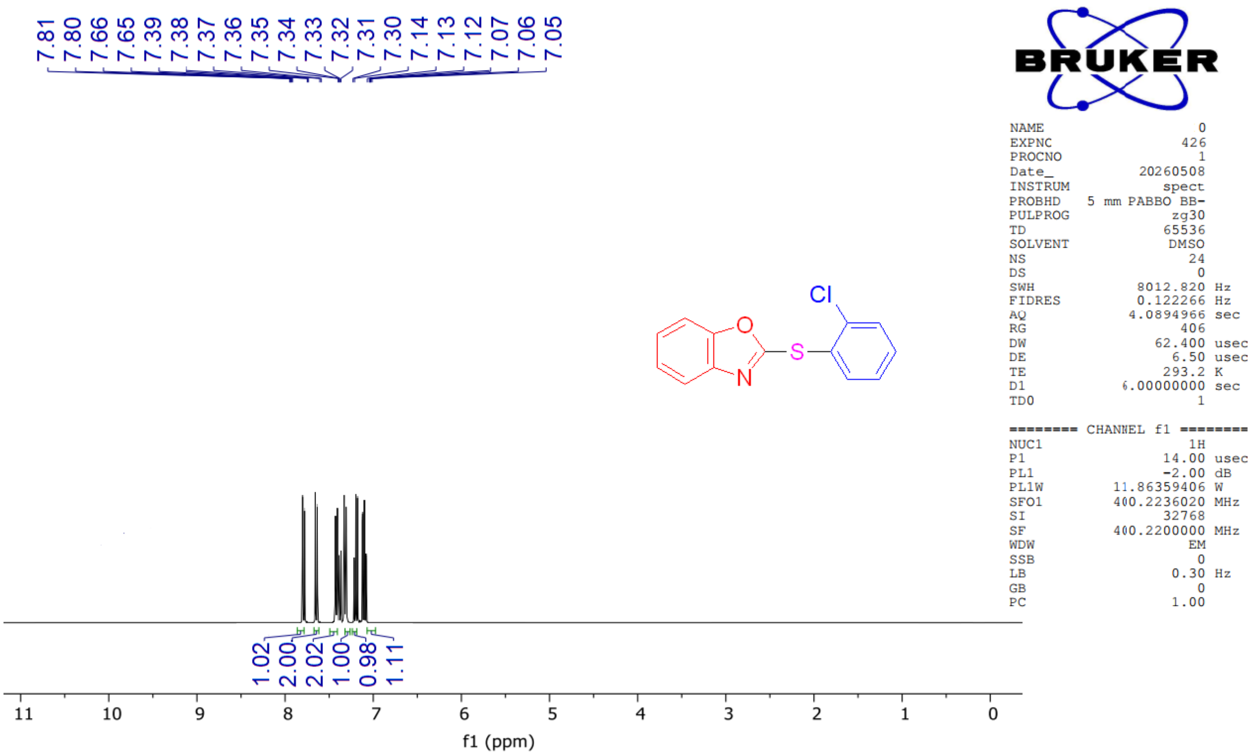

7.81
7.80
7.66
7.65
7.39
7.38
7.37
7.36
7.35
7.34
7.33
7.32
7.31
7.30
7.14
7.13
7.12
7.07
7.06
7.05
Cl
O
N
S
BRUKER
NAME 0
EXPNC 426
PROCNO 1
Date_ 20260508
INSTRUM spect
PROBHD 5 mm PABBO BB-
PULPROG zg30
TD 65536
SOLVENT DMSO
NS 24
DS 0
SWH 8012.820 Hz
FIDRES 0.122266 Hz
AQ 4.0894966 sec
RG 406
DW 62.400 usec
DE 6.50 usec
TE 293.2 K
D1 6.00000000 sec
TD0 1
======== CHANNEL f1 ========
NUC1 1H
P1 14.00 usec
PL1 -2.00 dB
PL1W 11.86359406 W
SFO1 400.2236020 MHz
SI 32768
SF 400.2200000 MHz
WDW EM
SSB 0
LB 0.30 Hz
GB 0
PC 1.00
1.02
2.00
2.02
1.00
0.98
1.11
11 10 9 8 7 6 5 4 3 2 1 0
f1 (ppm)

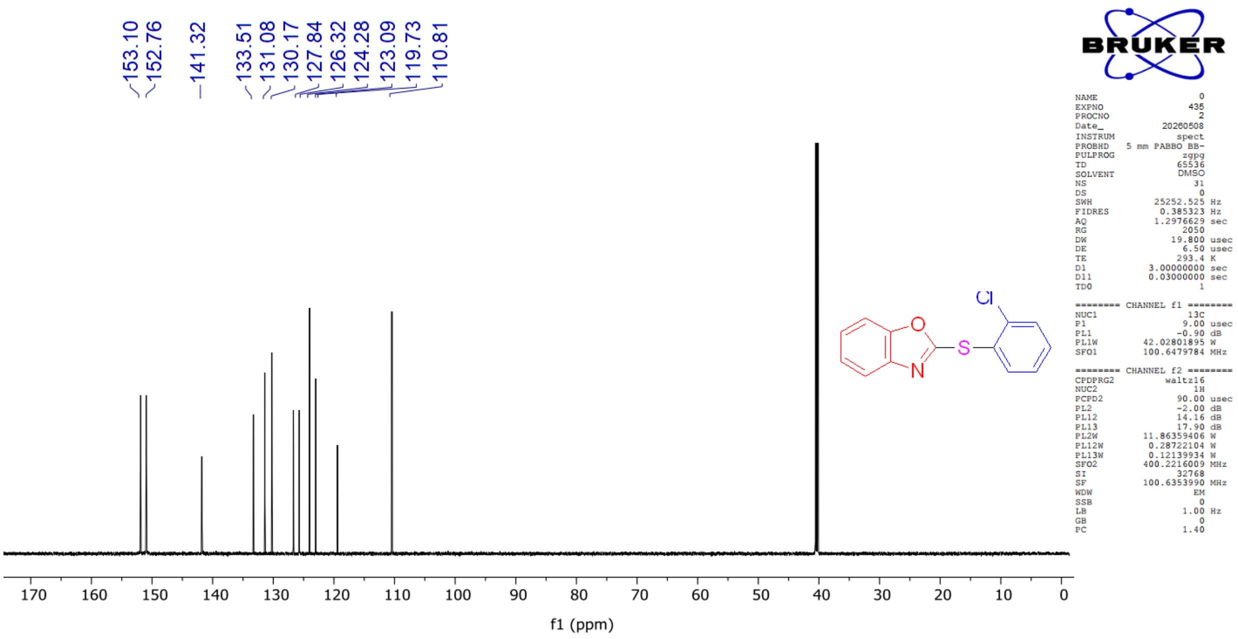
153.10
152.76
141.32
133.51
131.08
130.17
127.84
126.32
124.28
123.09
119.73
110.81
O
N
S
Cl
170 160 150 140 130 120 110 100 90 80 70 60 50 40 30 20 10 0
f1 (ppm)
NAME 0
EXPNO 435
PROCNO 2
Date_ 20260508
INSTRUM spect
PROBHD 5 mm PABBO BB-
PULPROG zgpg
TD 65536
SOLVENT DMSO
NS 31
DS 0
SWH 25252.525 Hz
FIDRES 0.385323 Hz
AQ 1.2976629 sec
RG 2050
DW 19.800 usec
DE 6.50 usec
TE 293.4 K
D1 3.00000000 sec
D11 0.03000000 sec
TD0 1
======== CHANNEL f1 ========
NUC1 13C
P1 9.00 usec
PL1 -0.90 dB
PL1W 42.02801895 W
SFO1 100.6479784 MHz
======== CHANNEL f2 ========
CPDPRG2 waltz16
NUC2 1H
PCPD2 90.00 usec
PL2 -2.00 dB
PL12 14.16 dB
PL13 17.90 dB
PL2W 11.86359406 W
PL12W 0.28722104 W
PL13W 0.12139934 W
SFO2 400.2216009 MHz
SI 32768
SF 100.6353990 MHz
WDW EM
SSB 0
LB 1.00 Hz
GB 0
PC 1.40

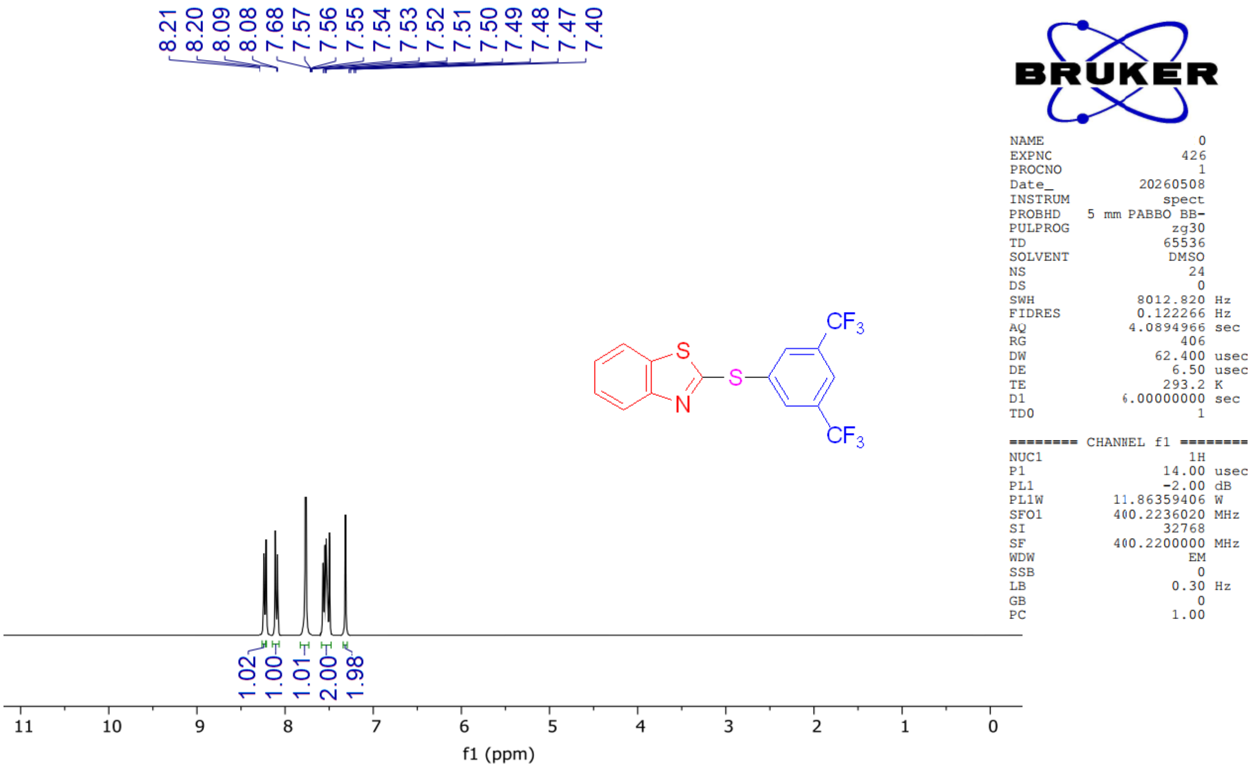
8.21
8.20
8.09
8.08
7.68
7.57
7.56
7.55
7.54
7.53
7.52
7.51
7.50
7.49
7.48
7.47
7.40
S
N
S
CF_3
CF_3
1.02
1.00
1.01
2.00
1.98
11 10 9 8 7 6 5 4 3 2 1 0
f1 (ppm)
NAME 0
EXPNO 426
PROCNO 1
Date_ 20260508
INSTRUM spect
PROBHD 5 mm PABBO BB-
PULPROG zg30
TD 65536
SOLVENT DMSO
NS 24
DS 0
SWH 8012.820 Hz
FIDRES 0.122266 Hz
AQ 4.0894966 sec
RG 406
DW 62.400 usec
DE 6.50 usec
TE 293.2 K
D1 6.00000000 sec
TD0 1
======== CHANNEL f1 ========
NUC1 1H
P1 14.00 usec
PL1 -2.00 dB
PL1W 11.86359406 W
SFO1 400.2236020 MHz
SI 32768
SF 400.2200000 MHz
WDW EM
SSB 0
LB 0.30 Hz
GB 0
PC 1.00

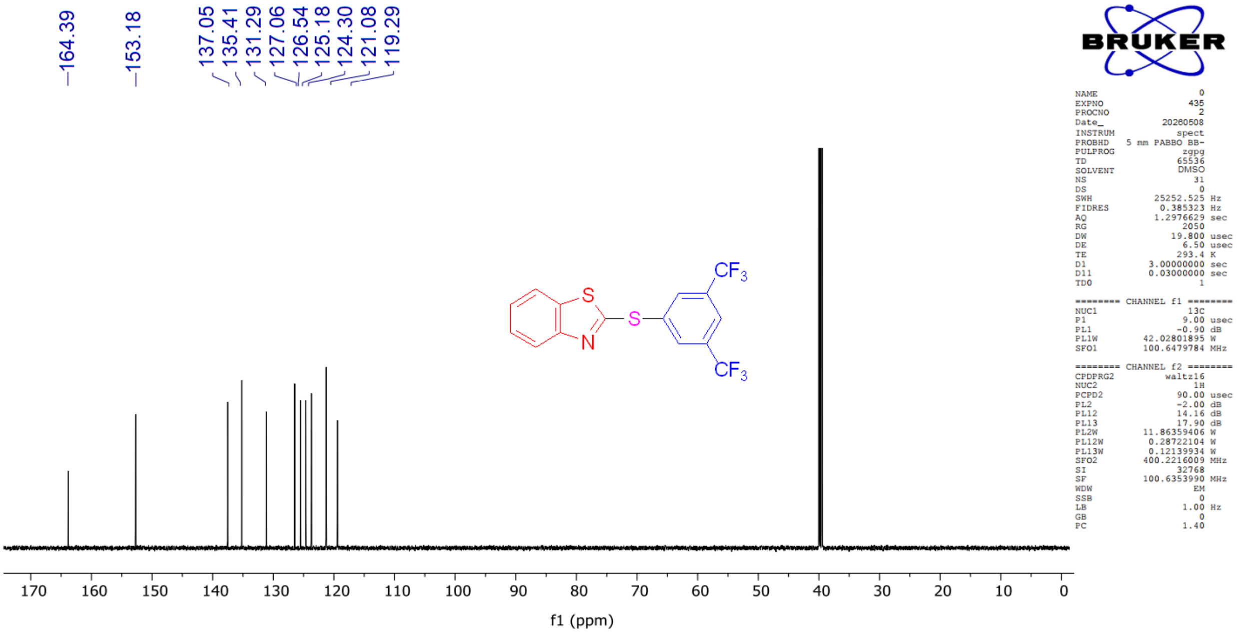
164.39
153.18
137.05
135.41
131.29
127.06
126.54
125.18
124.30
121.08
119.29
CF3
S
N
S
CF3
NAME 0
EXPNO 435
PROCNO 2
Date_ 20260508
INSTRUM spect
PROBHD 5 mm PABBO BB-
PULPROG zgpg
TD 65536
SOLVENT DMSO
NS 31
DS 0
SWH 25252.525 Hz
FIDRES 0.385323 Hz
AQ 1.2976629 sec
RG 2050
DW 19.800 usec
DE 6.50 usec
TE 293.4 K
D1 3.00000000 sec
D11 0.03000000 sec
TD0 1
======== CHANNEL f1 ========
NUC1 13C
P1 9.00 usec
PL1 -0.90 dB
PL1W 42.02801895 W
SFO1 100.6479784 MHz
======== CHANNEL f2 ========
CPDPRG2 waltz16
NUC2 1H
PCPD2 90.00 usec
PL2 -2.00 dB
PL12 14.16 dB
PL13 17.90 dB
PL2W 11.86359406 W
PL12W 0.28722104 W
PL13W 0.12139934 W
SFO2 400.2216009 MHz
SI 32768
SF 100.6353990 MHz
WDW EM
SSB 0
LB 1.00 Hz
GB 0
PC 1.40
170 160 150 140 130 120 110 100 90 80 70 60 50 40 30 20 10 0
f1 (ppm)

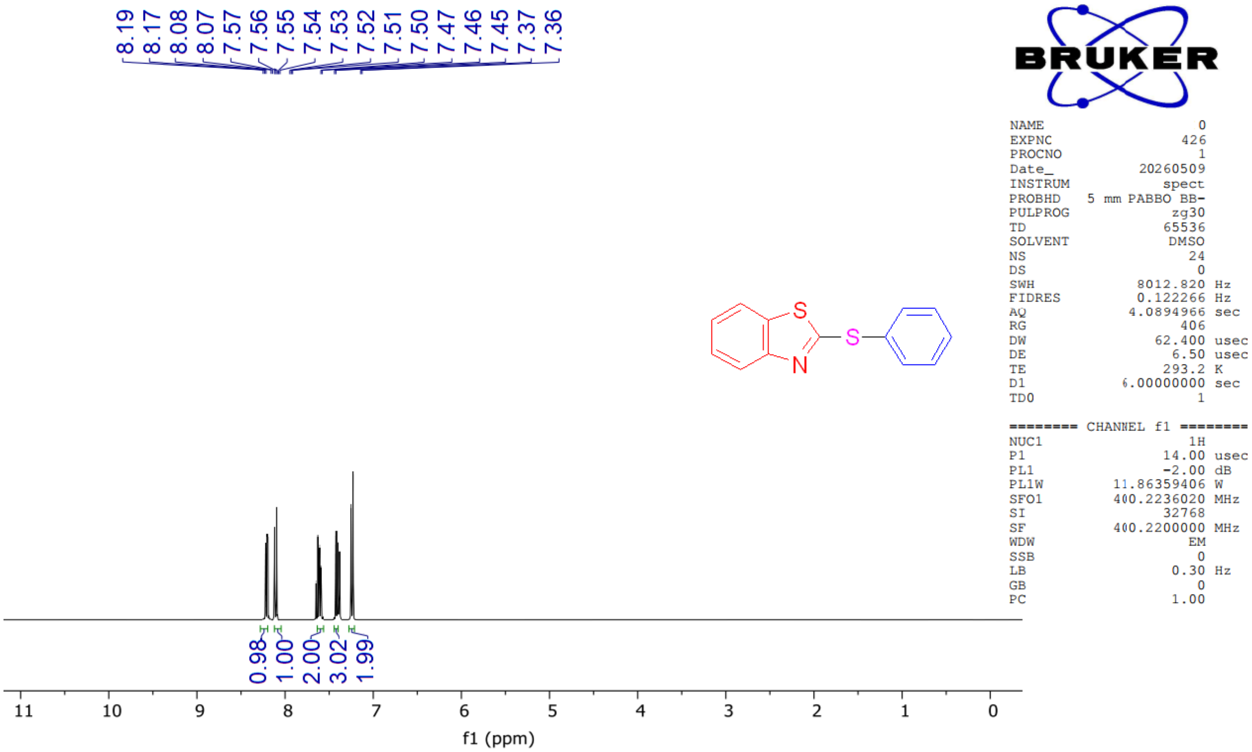
8.19
8.17
8.08
8.07
7.57
7.56
7.55
7.54
7.53
7.52
7.51
7.50
7.47
7.46
7.45
7.37
7.36
S
N
S
NAME 0
EXPNC 426
PROCNO 1
Date_ 20260509
INSTRUM spect
PROBHD 5 mm PABBO BB-
PULPROG zg30
TD 65536
SOLVENT DMSO
NS 24
DS 0
SWH 8012.820 Hz
FIDRES 0.122266 Hz
AQ 4.0894966 sec
RG 406
DW 62.400 usec
DE 6.50 usec
TE 293.2 K
D1 6.00000000 sec
TD0 1
======== CHANNEL f1 ========
NUC1 1H
P1 14.00 usec
PL1 -2.00 dB
PL1W 11.86359406 W
SFO1 400.2236020 MHz
SI 32768
SF 400.2200000 MHz
WDW EM
SSB 0
LB 0.30 Hz
GB 0
PC 1.00
0.98
1.00
2.00
3.02
1.99
11 10 9 8 7 6 5 4 3 2 1 0
f1 (ppm)

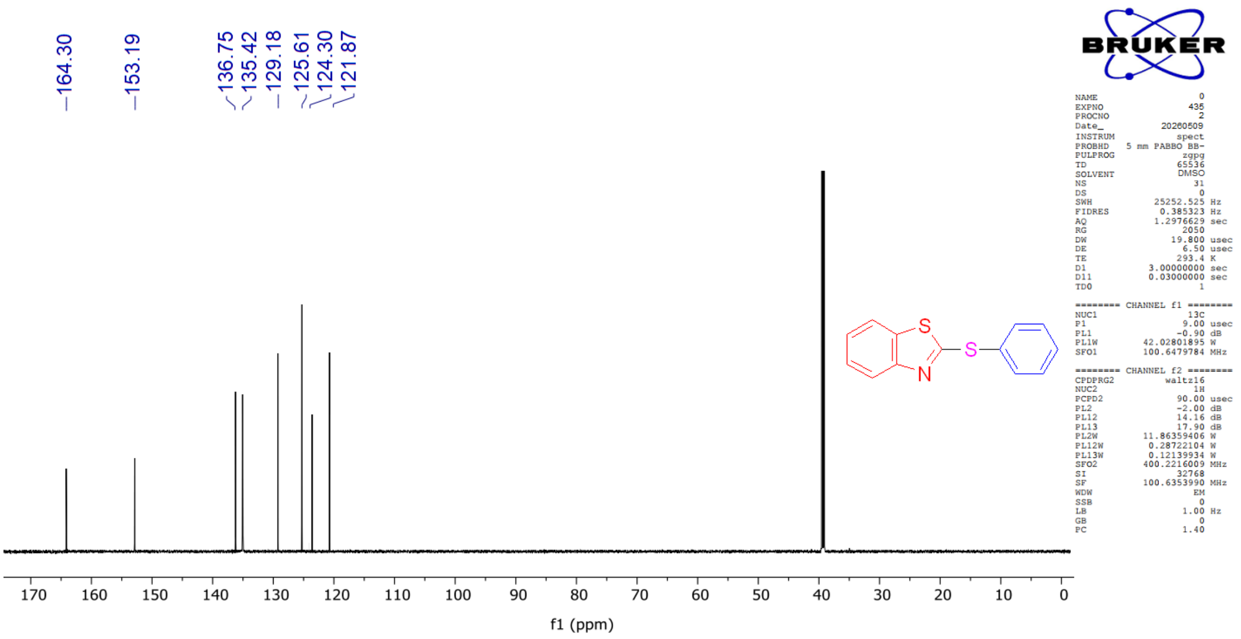
164.30
153.19
136.75
135.42
129.18
125.61
124.30
121.87
S
N
S
BRUKER
NAME 0
EXPNO 435
PROCNO 2
Date_ 20260509
INSTRUM spect
PROBHD 5 mm PABBO BB-
PULPROG zgpg
TD 65536
SOLVENT DMSO
NS 31
DS 0
SWH 25252.525 Hz
FIDRES 0.385323 Hz
AQ 1.2976629 sec
RG 2050
DW 19.800 usec
DE 6.50 usec
TE 293.4 K
D1 3.00000000 sec
D11 0.03000000 sec
TD0 1
======== CHANNEL f1 ========
NUC1 13C
P1 9.00 usec
PL1 -0.90 dB
PL1W 42.02801895 W
SFO1 100.6479784 MHz
======== CHANNEL f2 ========
CPDPRG2 waltz16
NUC2 1H
PCPD2 90.00 usec
PL2 -2.00 dB
PL12 14.16 dB
PL13 17.90 dB
PL2W 11.86359406 W
PL12W 0.28722104 W
PL13W 0.12139934 W
SFO2 400.2216009 MHz
SI 32768
SF 100.6353990 MHz
WDW EM
SSB 0
LB 1.00 Hz
GB 0
PC 1.40
170 160 150 140 130 120 110 100 90 80 70 60 50 40 30 20 10 0
f1 (ppm)

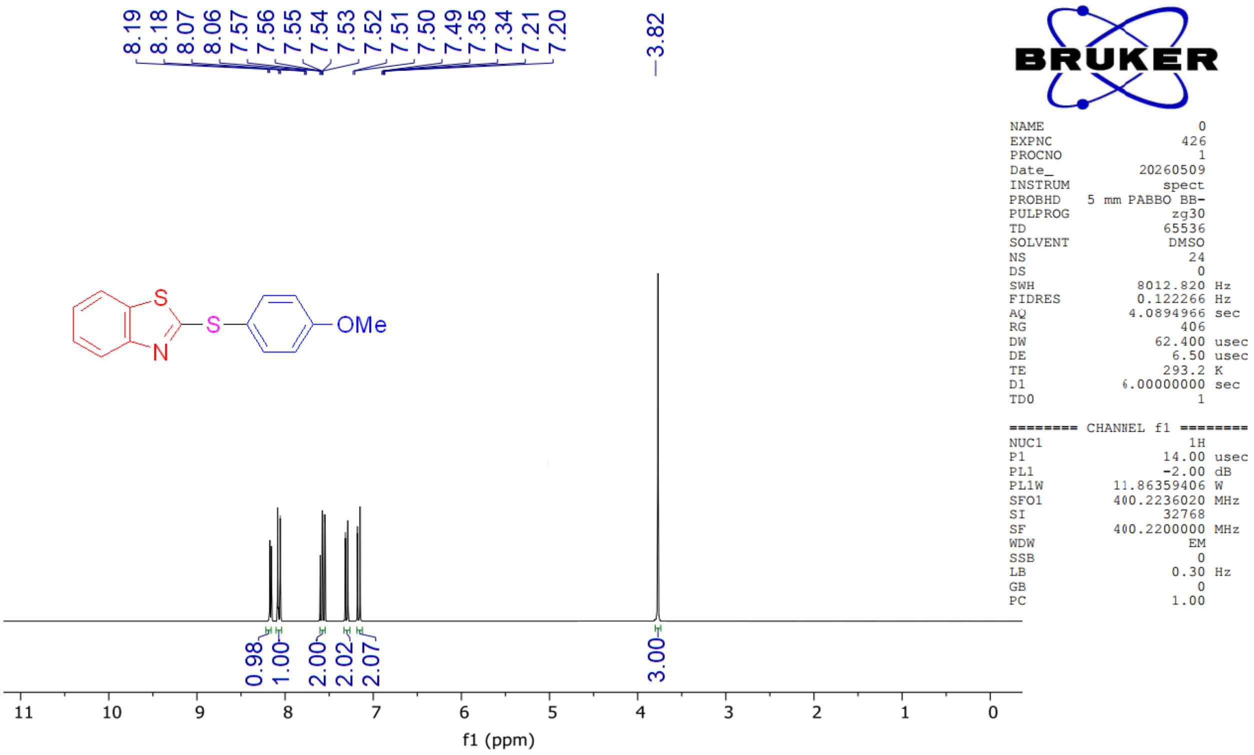
8.19
8.18
8.07
8.06
7.57
7.56
7.55
7.54
7.53
7.52
7.51
7.50
7.49
7.35
7.34
7.21
7.20
3.82
S
N
S
OMe
BRUKER
NAME 0
EXPNO 426
PROCNO 1
Date_ 20260509
INSTRUM spect
PROBHD 5 mm PABBO BB-
PULPROG zg30
TD 65536
SOLVENT DMSO
NS 24
DS 0
SWH 8012.820 Hz
FIDRES 0.122266 Hz
AQ 4.0894966 sec
RG 406
DW 62.400 usec
DE 6.50 usec
TE 293.2 K
D1 6.00000000 sec
TD0 1
======== CHANNEL f1 ========
NUC1 1H
P1 14.00 usec
PL1 -2.00 dB
PL1W 11.86359406 W
SFO1 400.2236020 MHz
SI 32768
SF 400.2200000 MHz
WDW EM
SSB 0
LB 0.30 Hz
GB 0
PC 1.00
0.98
1.00
2.00
2.02
2.07
3.00
11 10 9 8 7 6 5 4 3 2 1 0
f1 (ppm)

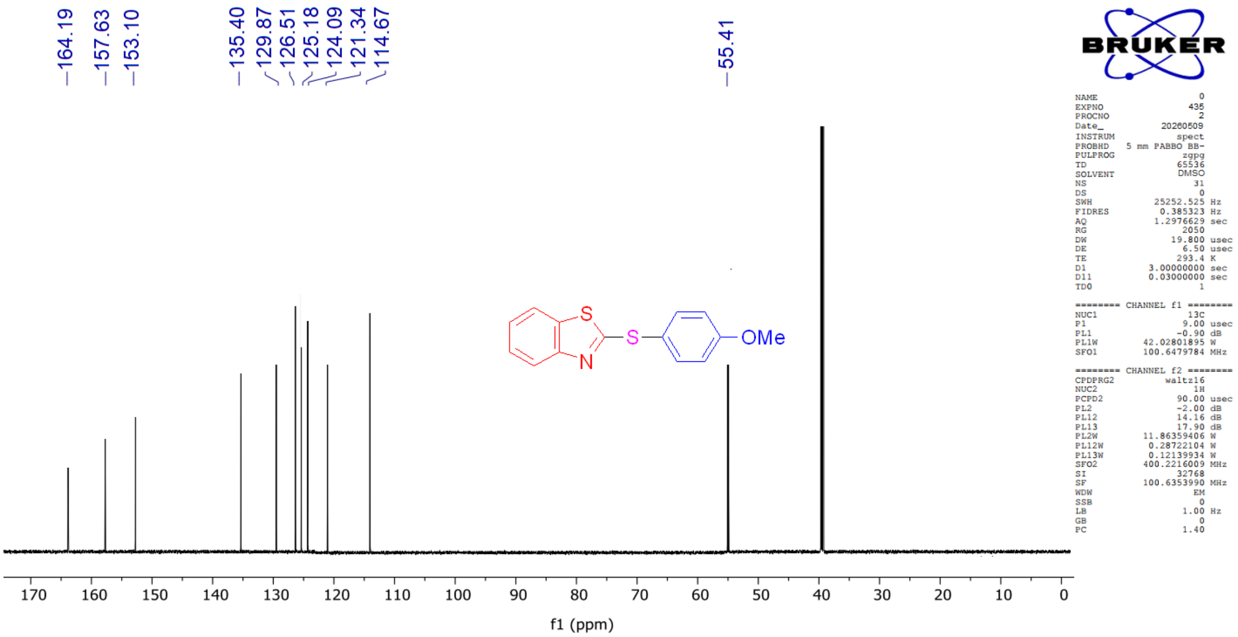

164.19
157.63
153.10
135.40
129.87
126.51
125.18
124.09
121.34
114.67
55.41
BRUKER
S
N
S
OMe
170 160 150 140 130 120 110 100 90 80 70 60 50 40 30 20 10 0
f1 (ppm)

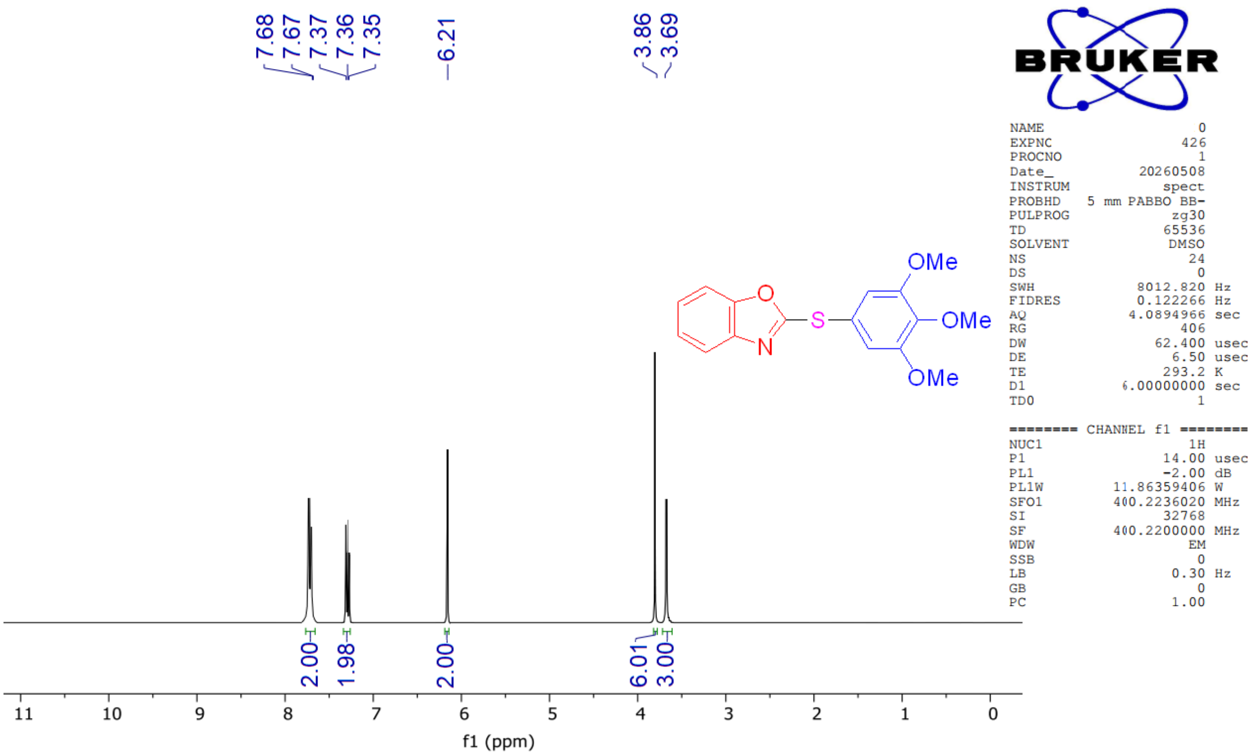

7.68
7.67
7.37
7.36
7.35
6.21
3.86
3.69
BRUKER
NAME 0
EXPNO 426
PROCNO 1
Date_ 20260508
INSTRUM spect
PROBHD 5 mm PABBO BB-
PULPROG zg30
TD 65536
SOLVENT DMSO
NS 24
DS 0
SWH 8012.820 Hz
FIDRES 0.122266 Hz
AQ 4.0894966 sec
RG 406
DW 62.400 usec
DE 6.50 usec
TE 293.2 K
D1 6.00000000 sec
TD0 1
======== CHANNEL f1 ========
NUC1 1H
P1 14.00 usec
PL1 -2.00 dB
PL1W 11.86359406 W
SFO1 400.2236020 MHz
SI 32768
SF 400.2200000 MHz
WDW EM
SSB 0
LB 0.30 Hz
GB 0
PC 1.00
OMe
OMe
OMe
O
N
S
2.00
1.98
2.00
6.01
3.00
11 10 9 8 7 6 5 4 3 2 1 0
f1 (ppm)

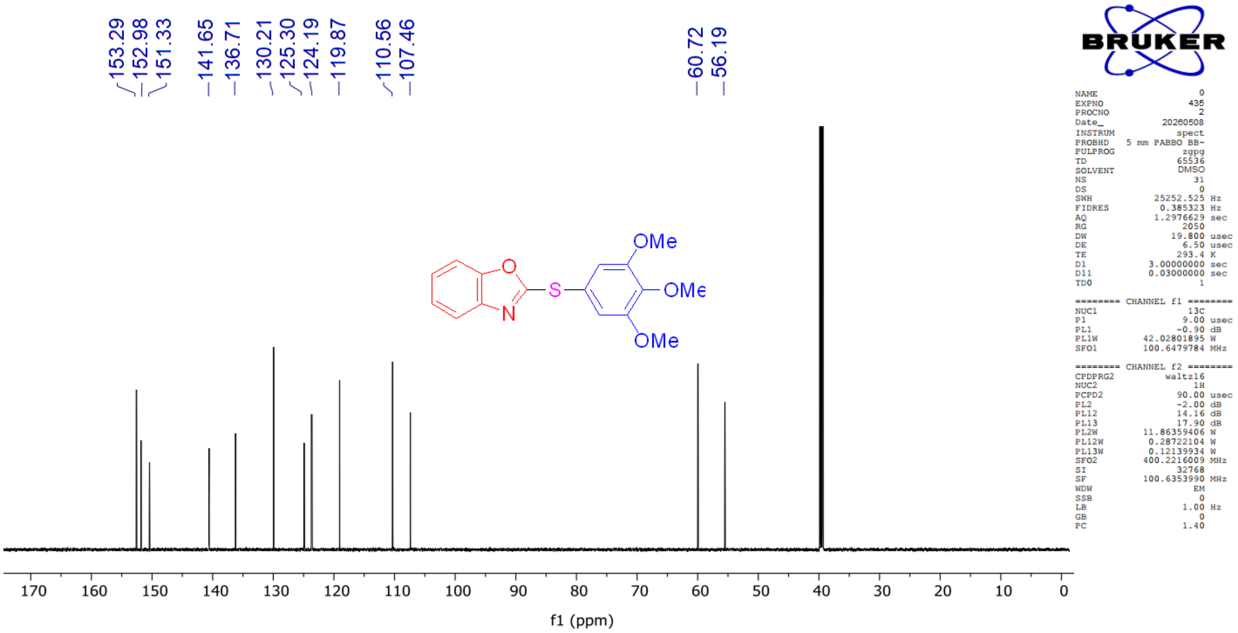

153.29
152.98
151.33
141.65
136.71
130.21
125.30
124.19
119.87
110.56
107.46
60.72
56.19
OMe
OMe
OMe
O
N
S
BRUKER
NAME 0
EXPNO 435
PROCNO 2
Date_ 20260508
INSTRUM spect
PROBHD 5 mm PABBO BB-
PULPROG zgpg
TD 65536
SOLVENT DMSO
NS 31
DS 0
SWH 25252.525 Hz
FIDRES 0.385323 Hz
AQ 1.2976629 sec
RG 2050
DW 19.800 usec
DE 6.50 usec
TE 293.4 K
D1 3.00000000 sec
D11 0.03000000 sec
TD0 1
======== CHANNEL f1 ========
NUC1 13C
P1 9.00 usec
PL1 -0.90 dB
PL1W 42.02801895 W
SFO1 100.6479784 MHz
======== CHANNEL f2 ========
CPDPRG2 waltz16
NUC2 1H
PCPD2 90.00 usec
PL2 -2.00 dB
PL12 14.16 dB
PL13 17.90 dB
PL2W 11.86359406 W
PL12W 0.28722104 W
PL13W 0.12139934 W
SFO2 400.2216009 MHz
SI 32768
SF 100.6353990 MHz
WDW EM
SSB 0
LB 1.00 Hz
GB 0
PC 1.40
170 160 150 140 130 120 110 100 90 80 70 60 50 40 30 20 10 0
f1 (ppm)

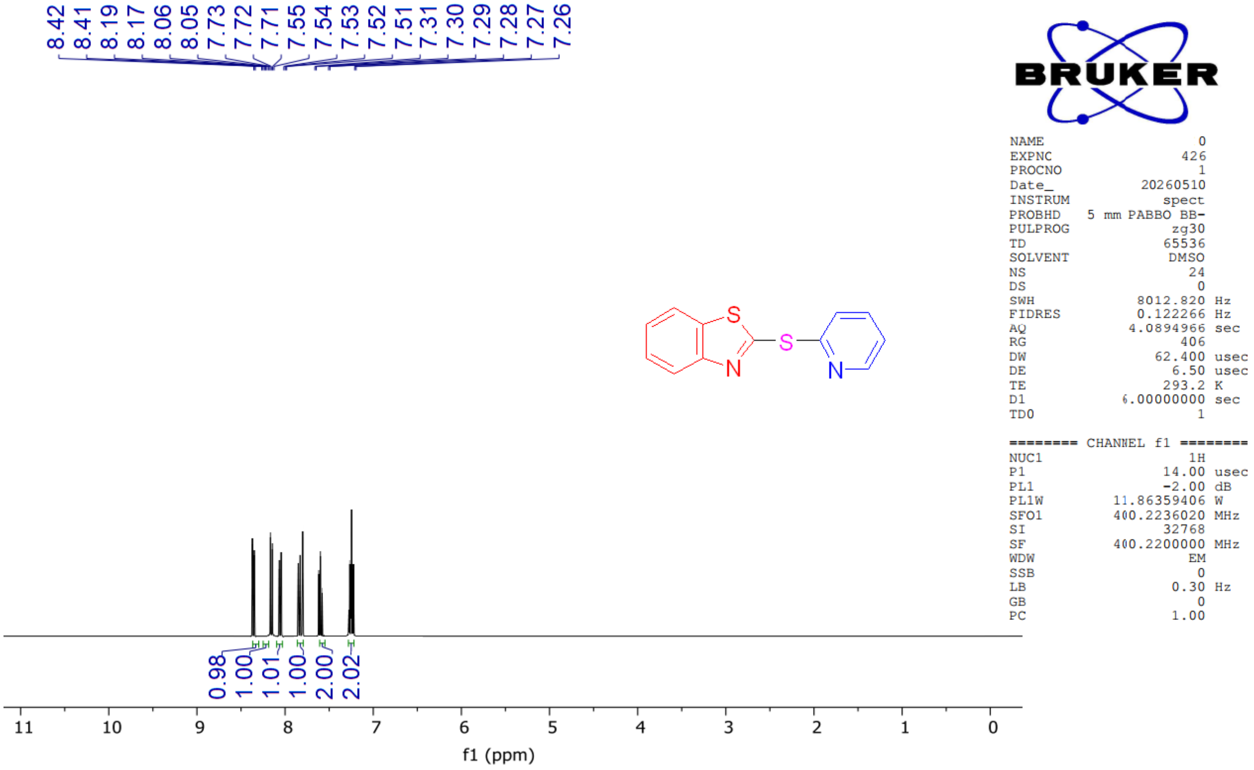

8.42
8.41
8.19
8.17
8.06
8.05
7.73
7.72
7.71
7.55
7.54
7.53
7.52
7.51
7.31
7.30
7.29
7.28
7.27
7.26
S
N
S
N
BRUKER
NAME 0
EXPNO 426
PROCNO 1
Date_ 20260510
INSTRUM spect
PROBHD 5 mm PABBO BB-
PULPROG zg30
TD 65536
SOLVENT DMSO
NS 24
DS 0
SWH 8012.820 Hz
FIDRES 0.122266 Hz
AQ 4.0894966 sec
RG 406
DW 62.400 usec
DE 6.50 usec
TE 293.2 K
D1 6.00000000 sec
TD0 1
======== CHANNEL f1 ========
NUC1 1H
P1 14.00 usec
PL1 -2.00 dB
PL1W 11.86359406 W
SFO1 400.2236020 MHz
SI 32768
SF 400.2200000 MHz
WDW EM
SSB 0
LB 0.30 Hz
GB 0
PC 1.00
0.98
1.00
1.01
1.00
2.00
2.02
11 10 9 8 7 6 5 4 3 2 1 0
f1 (ppm)

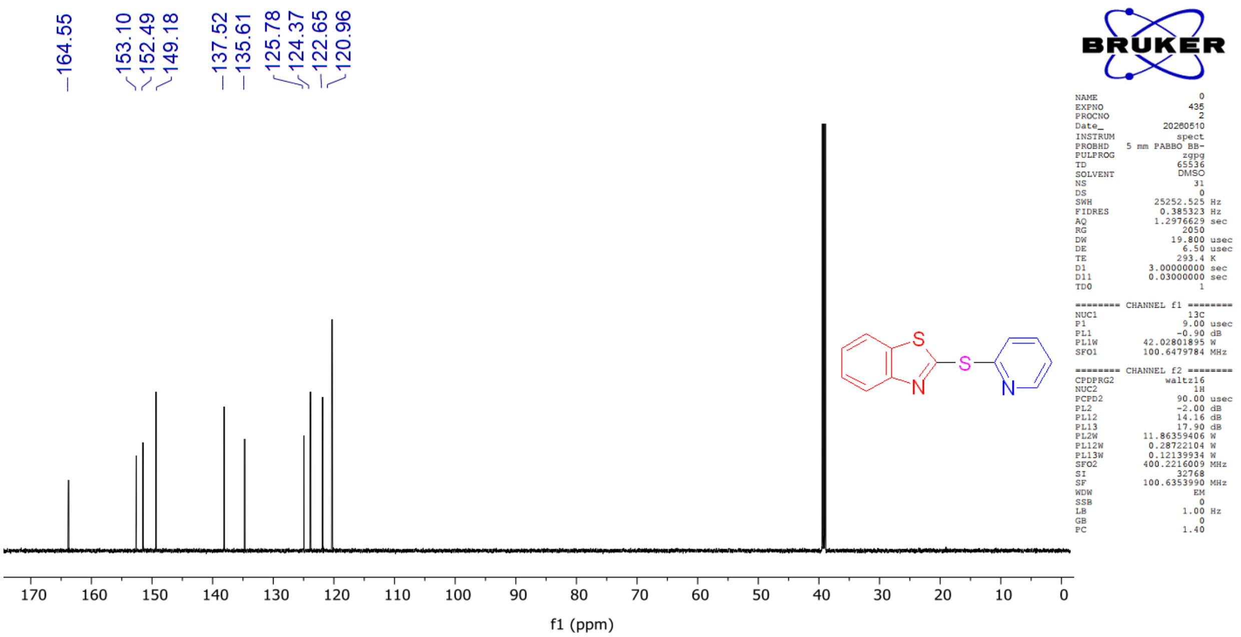
164.55
153.10
152.49
149.18
137.52
135.61
125.78
124.37
122.65
120.96
BRUKER
NAME 0
EXPNO 435
PROCNO 2
Date_ 20260510
INSTRUM spect
PROBHD 5 mm PABBO BB-
PULPROG zgpg
TD 65536
SOLVENT DMSO
NS 31
DS 0
SWH 25252.525 Hz
FIDRES 0.385323 Hz
AQ 1.2976629 sec
RG 2050
DW 19.800 usec
DE 6.50 usec
TE 293.4 K
D1 3.00000000 sec
D11 0.03000000 sec
TD0 1
======== CHANNEL f1 ========
NUC1 13C
P1 9.00 usec
PL1 -0.90 dB
PL1W 42.02801895 W
SFO1 100.6479784 MHz
======== CHANNEL f2 ========
CPDPRG2 waltz16
NUC2 1H
PCPD2 90.00 usec
PL2 -2.00 dB
PL12 14.16 dB
PL13 17.90 dB
PL2W 11.86359406 W
PL12W 0.28722104 W
PL13W 0.12139934 W
SFO2 400.2216009 MHz
SI 32768
SF 100.6353990 MHz
WDW EM
SSB 0
LB 1.00 Hz
GB 0
PC 1.40
S
N
S
N
170 160 150 140 130 120 110 100 90 80 70 60 50 40 30 20 10 0
f1 (ppm)

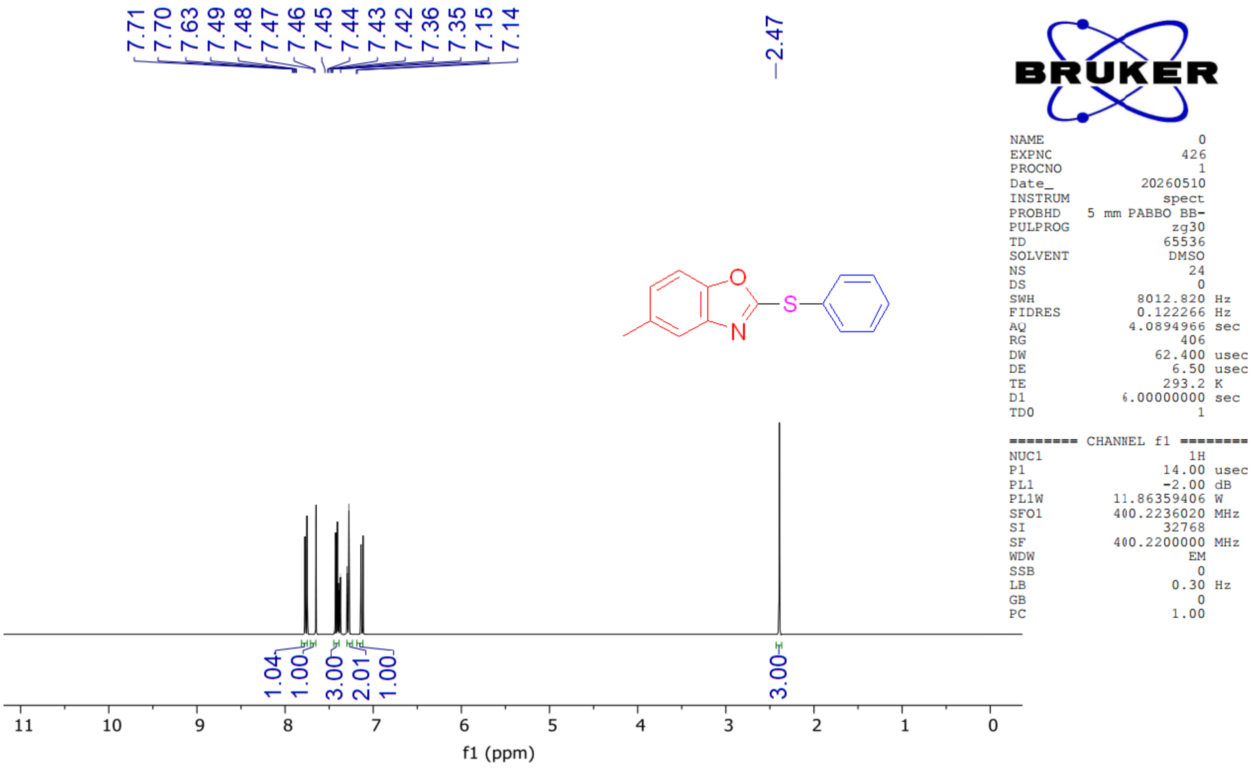
7.71
7.70
7.63
7.49
7.48
7.47
7.46
7.45
7.44
7.43
7.42
7.36
7.35
7.15
7.14
2.47
BRUKER
NAME 0
EXPNO 426
PROCNO 1
Date_ 20260510
INSTRUM spect
PROBHD 5 mm PABBO BB-
PULPROG zg30
TD 65536
SOLVENT DMSO
NS 24
DS 0
SWH 8012.820 Hz
FIDRES 0.122266 Hz
AQ 4.0894966 sec
RG 406
DW 62.400 usec
DE 6.50 usec
TE 293.2 K
D1 6.00000000 sec
TD0 1
======== CHANNEL f1 ========
NUC1 1H
P1 14.00 usec
PL1 -2.00 dB
PL1W 11.86359406 W
SFO1 400.2236020 MHz
SI 32768
SF 400.2200000 MHz
WDW EM
SSB 0
LB 0.30 Hz
GB 0
PC 1.00
O
N
S
1.04
1.00
3.00
2.01
1.00
3.00
11 10 9 8 7 6 5 4 3 2 1 0
f1 (ppm)

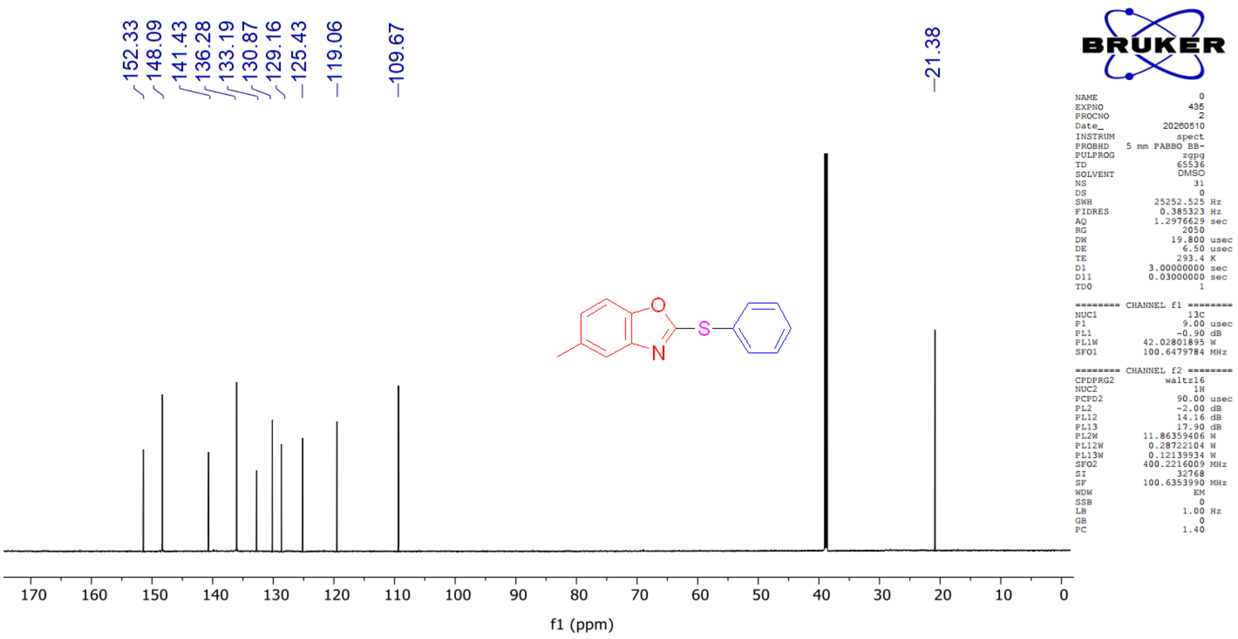
152.33
148.09
141.43
136.28
133.19
130.87
129.16
125.43
119.06
109.67
21.38
O
N
S
BRUKER
NAME 0
EXPNO 435
PROCNO 2
Date_ 20260510
INSTRUM spect
PROBHD 5 mm PABBO BB-
PULPROG zgpg
TD 65536
SOLVENT DMSO
NS 31
DS 0
SWH 25252.525 Hz
FIDRES 0.385323 Hz
AQ 1.2976629 sec
RG 2050
DW 19.800 usec
DE 6.50 usec
TE 293.4 K
D1 3.00000000 sec
D11 0.03000000 sec
TD0 1
======== CHANNEL f1 ========
NUC1 13C
P1 9.00 usec
PL1 -0.90 dB
PL1W 42.02801895 W
SFO1 100.6479784 MHz
======== CHANNEL f2 ========
CPDPRG2 waltz16
NUC2 1H
PCPD2 90.00 usec
PL2 -2.00 dB
PL12 14.16 dB
PL13 17.90 dB
PL2W 11.86359406 W
PL12W 0.28722104 W
PL13W 0.12139934 W
SFO2 400.2216009 MHz
SI 32768
SF 100.6353990 MHz
WDW EM
SSB 0
LB 1.00 Hz
GB 0
PC 1.40
170 160 150 140 130 120 110 100 90 80 70 60 50 40 30 20 10 0
f1 (ppm)

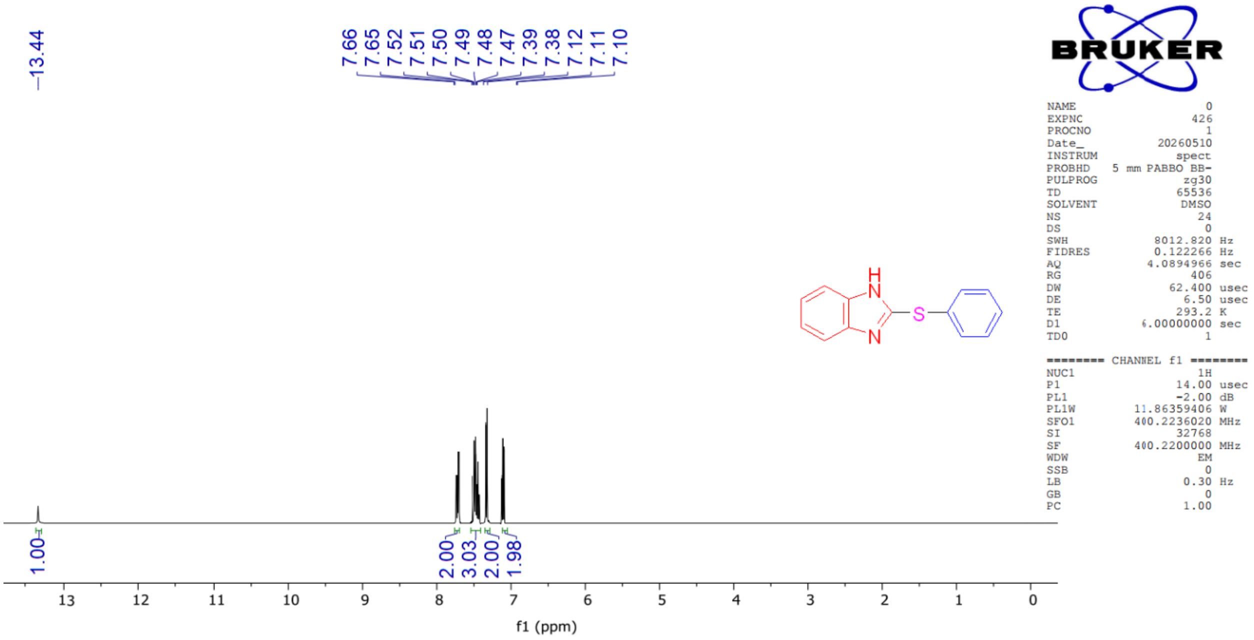
13.44
7.66
7.65
7.52
7.51
7.50
7.49
7.48
7.47
7.39
7.38
7.12
7.11
7.10
H
N
N
S
BRUKER
NAME 0
EXPNO 426
PROCNO 1
Date_ 20260510
INSTRUM spect
PROBHD 5 mm PABBO BB-
PULPROG zg30
TD 65536
SOLVENT DMSO
NS 24
DS 0
SWH 8012.820 Hz
FIDRES 0.122266 Hz
AQ 4.0894966 sec
RG 406
DW 62.400 usec
DE 6.50 usec
TE 293.2 K
D1 6.00000000 sec
TD0 1
======== CHANNEL f1 ========
NUC1 1H
P1 14.00 usec
PL1 -2.00 dB
PL1W 11.86359406 W
SFO1 400.2236020 MHz
SI 32768
SF 400.2200000 MHz
WDW EM
SSB 0
LB 0.30 Hz
GB 0
PC 1.00
1.00
2.00
3.03
2.00
1.98
13 12 11 10 9 8 7 6 5 4 3 2 1 0
f1 (ppm)

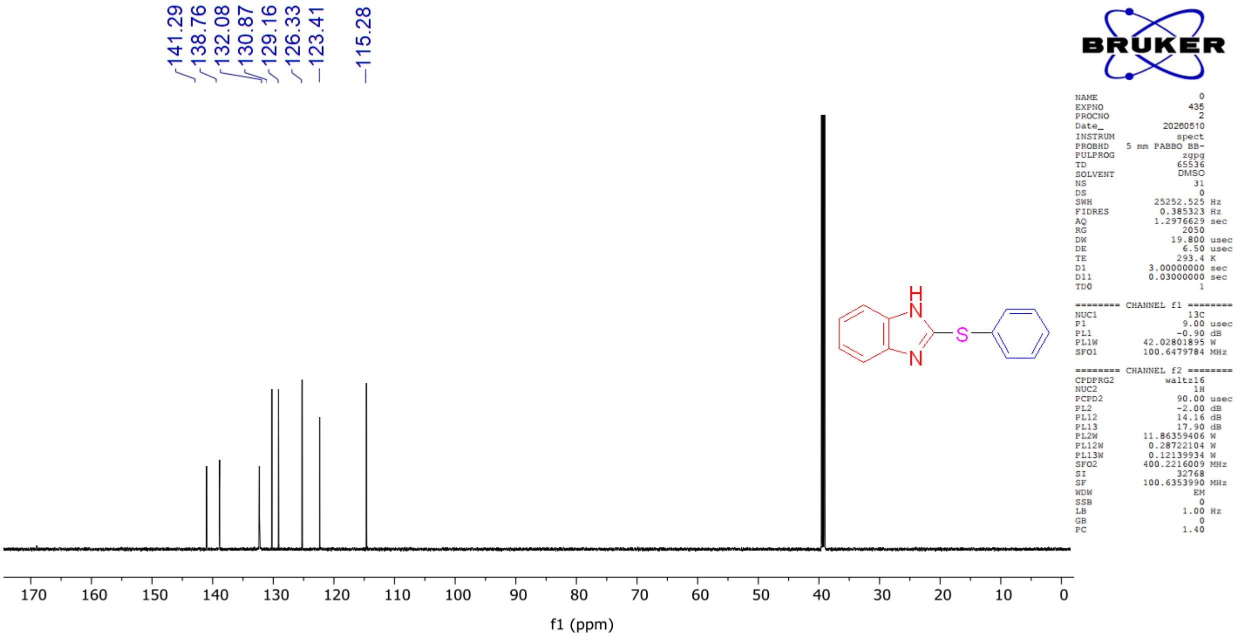
141.29
138.76
132.08
130.87
129.16
126.33
123.41
115.28
BRUKER
NAME 0
EXPNO 435
PROCNO 2
Date_ 20260510
INSTRUM spect
PROBHD 5 mm PABBO BB-
PULPROG zgpg
TD 65536
SOLVENT DMSO
NS 31
DS 0
SWH 25252.525 Hz
FIDRES 0.385323 Hz
AQ 1.2976629 sec
RG 2050
DW 19.800 usec
DE 6.50 usec
TE 293.4 K
D1 3.00000000 sec
D11 0.03000000 sec
TD0 1
======== CHANNEL f1 ========
NUC1 13C
P1 9.00 usec
PL1 -0.90 dB
PL1W 42.02801895 W
SFO1 100.6479784 MHz
======== CHANNEL f2 ========
CPDPRG2 waltz16
NUC2 1H
PCPD2 90.00 usec
PL2 -2.00 dB
PL12 14.16 dB
PL13 17.90 dB
PL2W 11.86359406 W
PL12W 0.28722104 W
PL13W 0.12139934 W
SFO2 400.2216009 MHz
SI 32768
SF 100.6353990 MHz
WDW EM
SSB 0
LB 1.00 Hz
GB 0
PC 1.40
H
N
S
N
170 160 150 140 130 120 110 100 90 80 70 60 50 40 30 20 10 0
f1 (ppm)

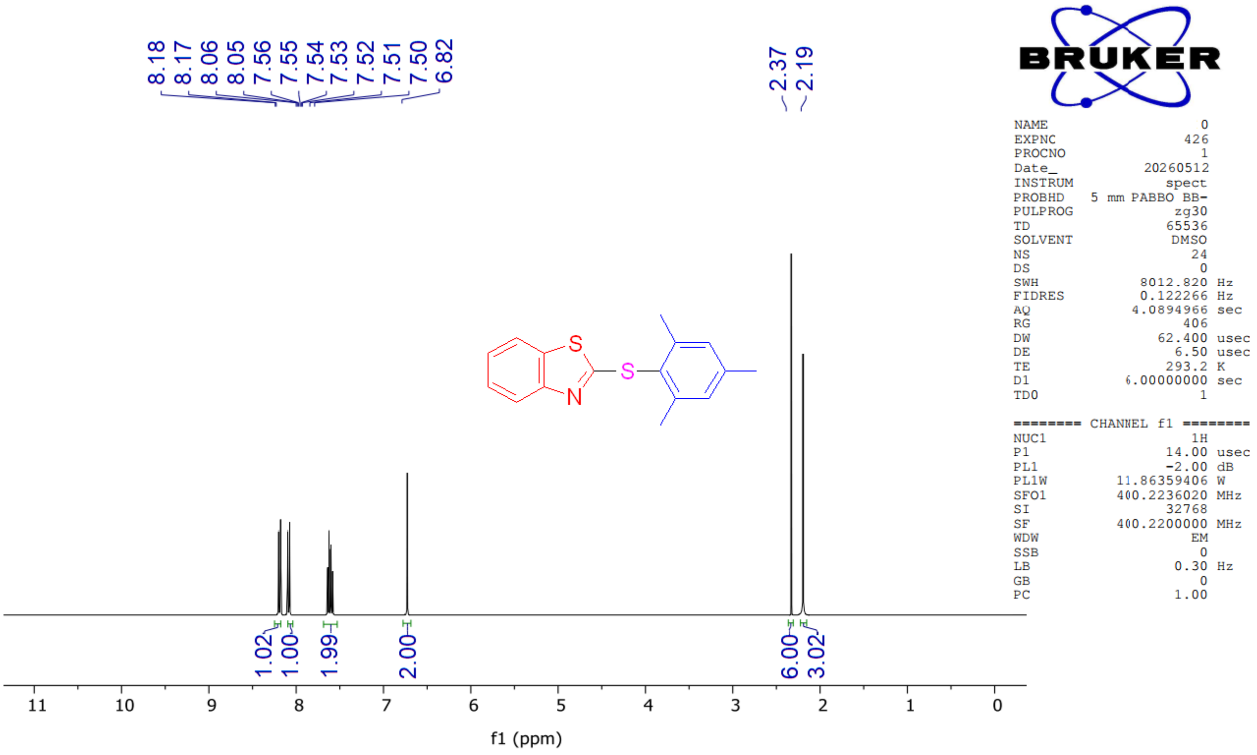
8.18
8.17
8.06
8.05
7.56
7.55
7.54
7.53
7.52
7.51
7.50
6.82
2.37
2.19
BRUKER
NAME 0
EXPNC 426
PROCNO 1
Date_ 20260512
INSTRUM spect
PROBHD 5 mm PABBO BB-
PULPROG zg30
TD 65536
SOLVENT DMSO
NS 24
DS 0
SWH 8012.820 Hz
FIDRES 0.122266 Hz
AQ 4.0894966 sec
RG 406
DW 62.400 usec
DE 6.50 usec
TE 293.2 K
D1 6.00000000 sec
TD0 1
======== CHANNEL f1 ========
NUC1 1H
P1 14.00 usec
PL1 -2.00 dB
PL1W 11.86359406 W
SFO1 400.2236020 MHz
SI 32768
SF 400.2200000 MHz
WDW EM
SSB 0
LB 0.30 Hz
GB 0
PC 1.00
S
N
S
1.02
1.00
1.99
2.00
6.00
3.02
11 10 9 8 7 6 5 4 3 2 1 0
f1 (ppm)

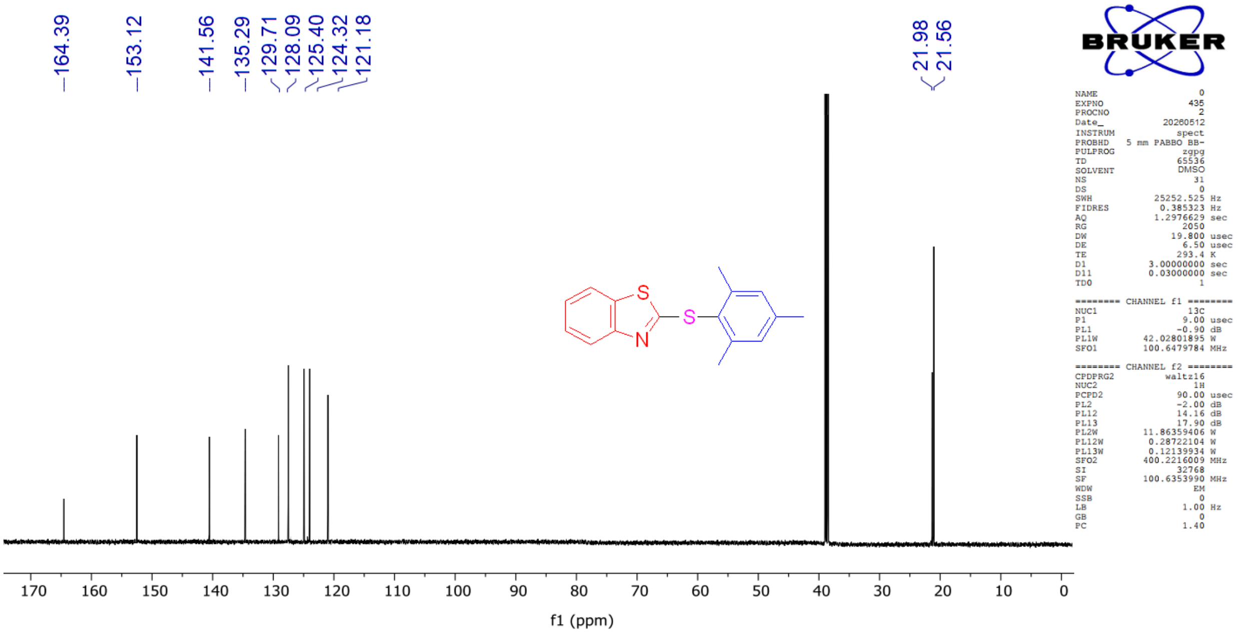
164.39
153.12
141.56
135.29
129.71
128.09
125.40
124.32
121.18
21.98
21.56
BRUKER
NAME 0
EXPNO 435
PROCNO 2
Date_ 20260512
INSTRUM spect
PROBHD 5 mm PABBO BB-
PULPROG zgpg
TD 65536
SOLVENT DMSO
NS 31
DS 0
SWH 25252.525 Hz
FIDRES 0.385323 Hz
AQ 1.2976629 sec
RG 2050
DW 19.800 usec
DE 6.50 usec
TE 293.4 K
D1 3.00000000 sec
D11 0.03000000 sec
TD0 1
======= CHANNEL f1 =======
NUC1 13C
P1 9.00 usec
PL1 -0.90 dB
PL1W 42.02801895 W
SFO1 100.6479784 MHz
======= CHANNEL f2 =======
CPDPRG2 waltz16
NUC2 1H
PCPD2 90.00 usec
PL2 -2.00 dB
PL12 14.16 dB
PL13 17.90 dB
PL2W 11.86359406 W
PL12W 0.28722104 W
PL13W 0.12139934 W
SFO2 400.2216009 MHz
SI 32768
SF 100.6353990 MHz
WDW EM
SSB 0
LB 1.00 Hz
GB 0
PC 1.40
S
N
S
170 160 150 140 130 120 110 100 90 80 70 60 50 40 30 20 10 0
f1 (ppm)

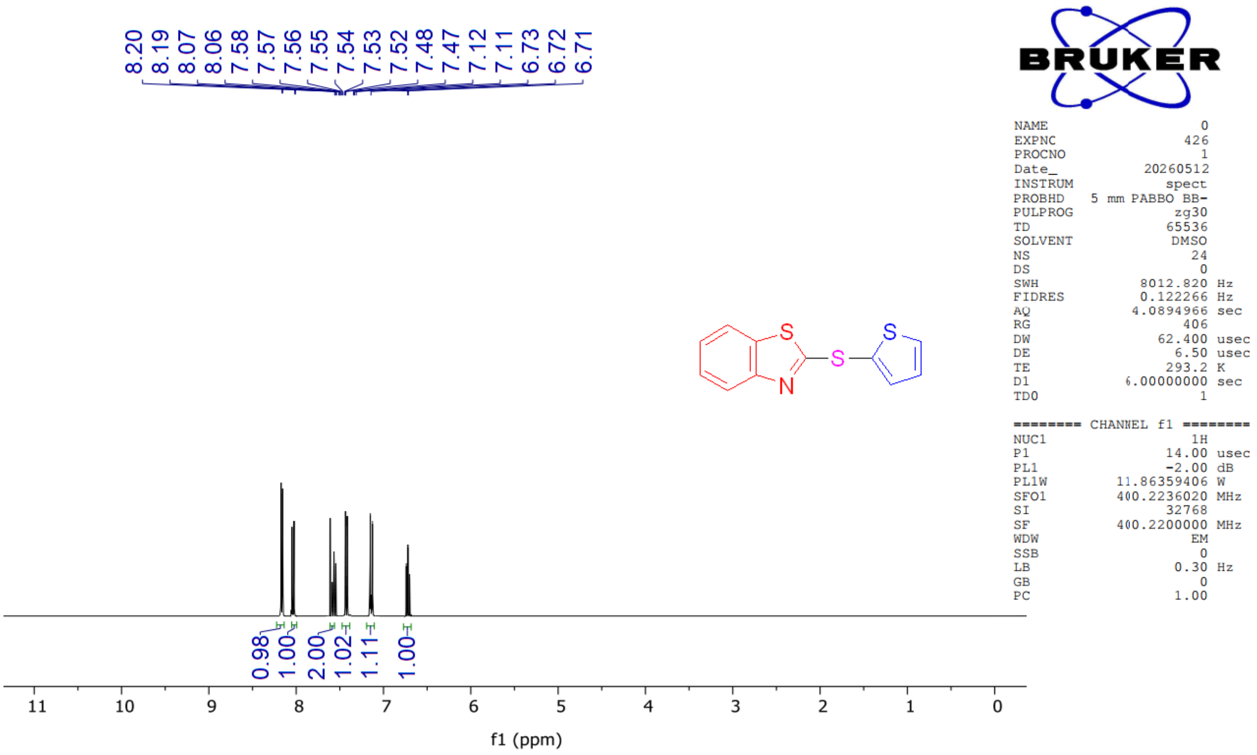
8.20
8.19
8.07
8.06
7.58
7.57
7.56
7.55
7.54
7.53
7.52
7.48
7.47
7.12
7.11
6.73
6.72
6.71
BRUKER
NAME 0
EXPNC 426
PROCNO 1
Date_ 20260512
INSTRUM spect
PROBHD 5 mm PABBO BB-
PULPROG zg30
TD 65536
SOLVENT DMSO
NS 24
DS 0
SWH 8012.820 Hz
FIDRES 0.122266 Hz
AQ 4.0894966 sec
RG 406
DW 62.400 usec
DE 6.50 usec
TE 293.2 K
D1 6.00000000 sec
TD0 1
======== CHANNEL f1 ========
NUC1 1H
P1 14.00 usec
PL1 -2.00 dB
PL1W 11.86359406 W
SFO1 400.2236020 MHz
SI 32768
SF 400.2200000 MHz
WDW EM
SSB 0
LB 0.30 Hz
GB 0
PC 1.00
S
N
S
S
0.98
1.00
2.00
1.02
1.11
1.00
11 10 9 8 7 6 5 4 3 2 1 0
f1 (ppm)

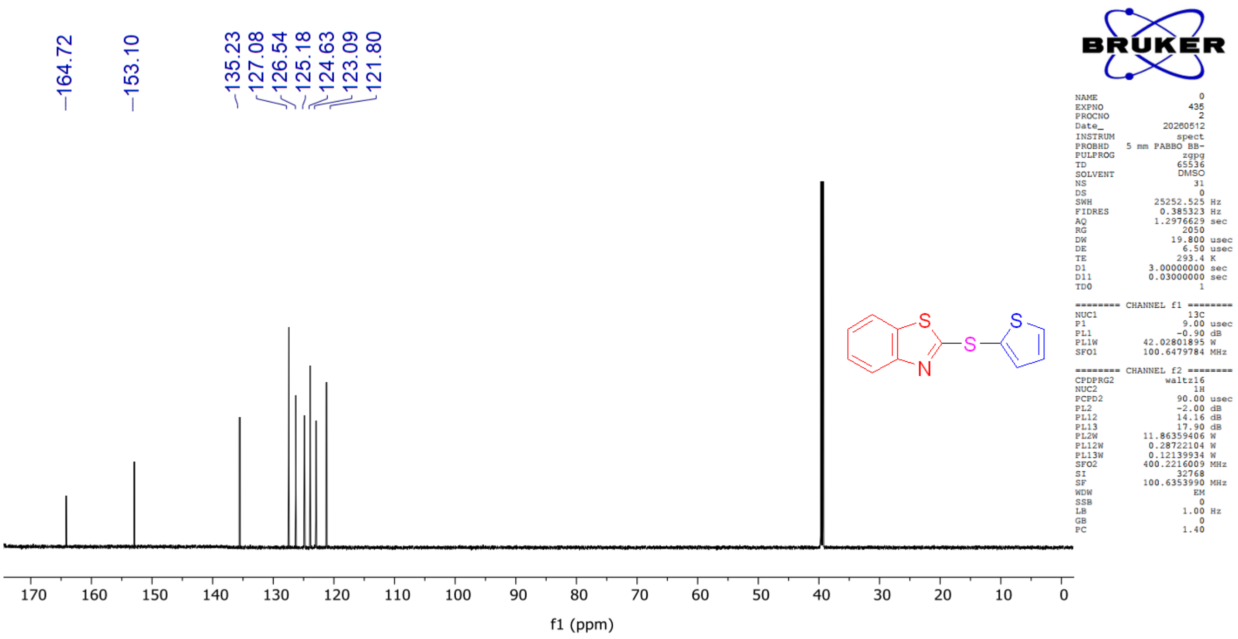

164.72
153.10
135.23
127.08
126.54
125.18
124.63
123.09
121.80
BRUKER
NAME 0
EXPNO 435
PROCNO 2
Date_ 20260512
INSTRUM spect
PROBHD 5 mm PABBO BB-
PULPROG zgpg
TD 65536
SOLVENT DMSO
NS 31
DS 0
SWH 25252.525 Hz
FIDRES 0.385323 Hz
AQ 1.2976629 sec
RG 2050
DW 19.800 usec
DE 6.50 usec
TE 293.4 K
D1 3.00000000 sec
D11 0.03000000 sec
TD0 1
======== CHANNEL f1 ========
NUC1 13C
P1 9.00 usec
PL1 -0.90 dB
PL1W 42.02801895 W
SFO1 100.6479784 MHz
======== CHANNEL f2 ========
CPDPRG2 waltz16
NUC2 1H
PCPD2 90.00 usec
PL2 -2.00 dB
PL12 14.16 dB
PL13 17.90 dB
PL2W 11.86359406 W
PL12W 0.28722104 W
PL13W 0.12139934 W
SFO2 400.2216009 MHz
SI 32768
SF 100.6353990 MHz
WDW EM
SSB 0
LB 1.00 Hz
GB 0
PC 1.40
S
N
S
S
170 160 150 140 130 120 110 100 90 80 70 60 50 40 30 20 10 0
f1 (ppm)

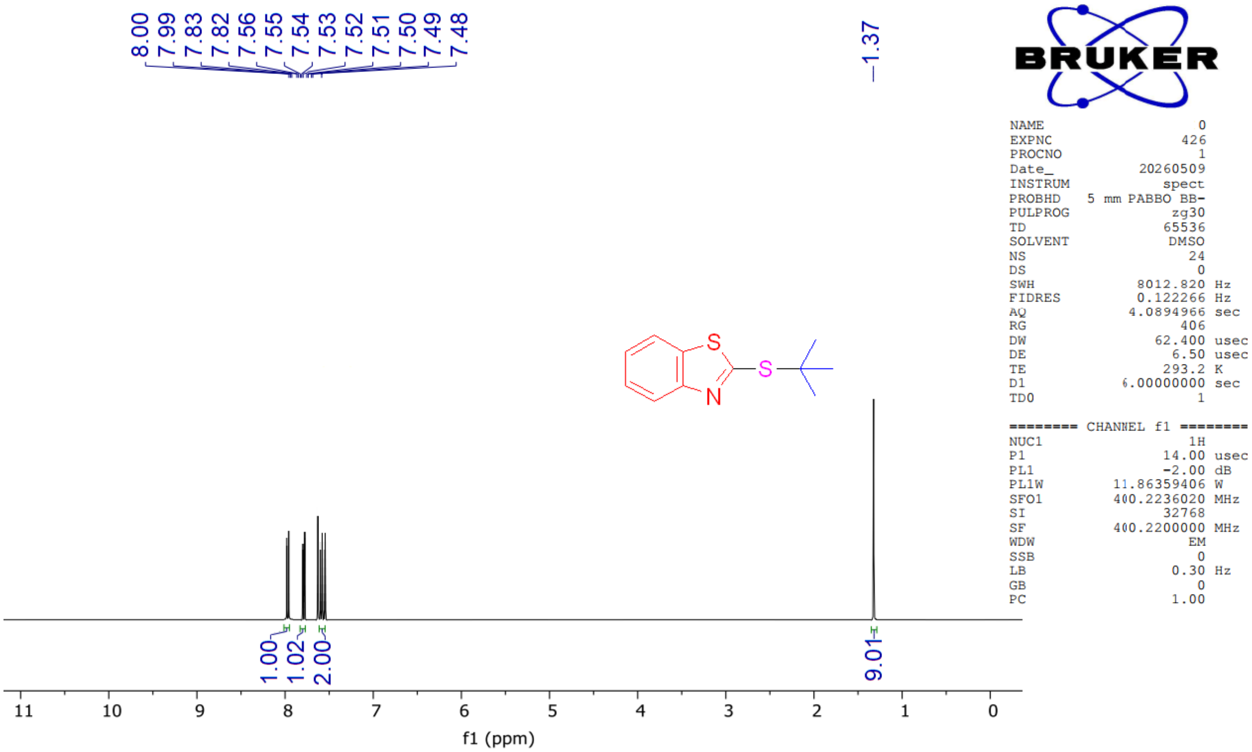

8.00
7.99
7.83
7.82
7.56
7.55
7.54
7.53
7.52
7.51
7.50
7.49
7.48
1.37
BRUKER
NAME 0
EXPNO 426
PROCNO 1
Date_ 20260509
INSTRUM spect
PROBHD 5 mm PABBO BB-
PULPROG zg30
TD 65536
SOLVENT DMSO
NS 24
DS 0
SWH 8012.820 Hz
FIDRES 0.122266 Hz
AQ 4.0894966 sec
RG 406
DW 62.400 usec
DE 6.50 usec
TE 293.2 K
D1 6.00000000 sec
TD0 1
======== CHANNEL f1 ========
NUC1 1H
P1 14.00 usec
PL1 -2.00 dB
PL1W 11.86359406 W
SFO1 400.2236020 MHz
SI 32768
SF 400.2200000 MHz
WDW EM
SSB 0
LB 0.30 Hz
GB 0
PC 1.00
S
N
S
1.00
1.02
2.00
9.01
11 10 9 8 7 6 5 4 3 2 1 0
f1 (ppm)

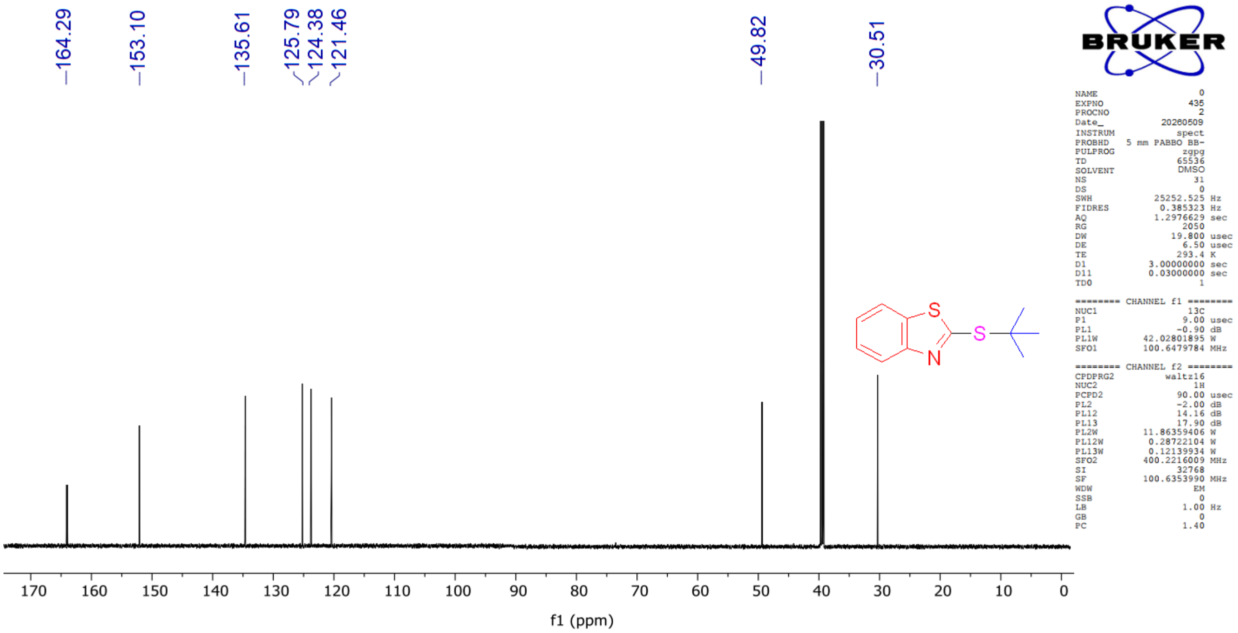
164.29
153.10
135.61
125.79
124.38
121.46
49.82
30.51
BRUKER
NAME 0
EXPNO 435
PROCNO 2
Date_ 20260509
INSTRUM spect
PROBHD 5 mm PABBO BB-
PULPROG zgpg
TD 65536
SOLVENT DMSO
NS 31
DS 0
SWH 25252.525 Hz
FIDRES 0.385323 Hz
AQ 1.2976629 sec
RG 2050
DW 19.800 usec
DE 6.50 usec
TE 293.4 K
D1 3.00000000 sec
D11 0.03000000 sec
TD0 1
======== CHANNEL f1 ========
NUC1 13C
P1 9.00 usec
PL1 -0.90 dB
PL1W 42.02801895 W
SFO1 100.6479784 MHz
======== CHANNEL f2 ========
CPDPRG2 waltz16
NUC2 1H
PCPD2 90.00 usec
PL2 -2.00 dB
PL12 14.16 dB
PL13 17.90 dB
PL2W 11.86359406 W
PL12W 0.28722104 W
PL13W 0.12139934 W
SFO2 400.2216009 MHz
SI 32768
SF 100.6353990 MHz
WDW EM
SSB 0
LB 1.00 Hz
GB 0
PC 1.40
S
N
S
170 160 150 140 130 120 110 100 90 80 70 60 50 40 30 20 10 0
f1 (ppm)

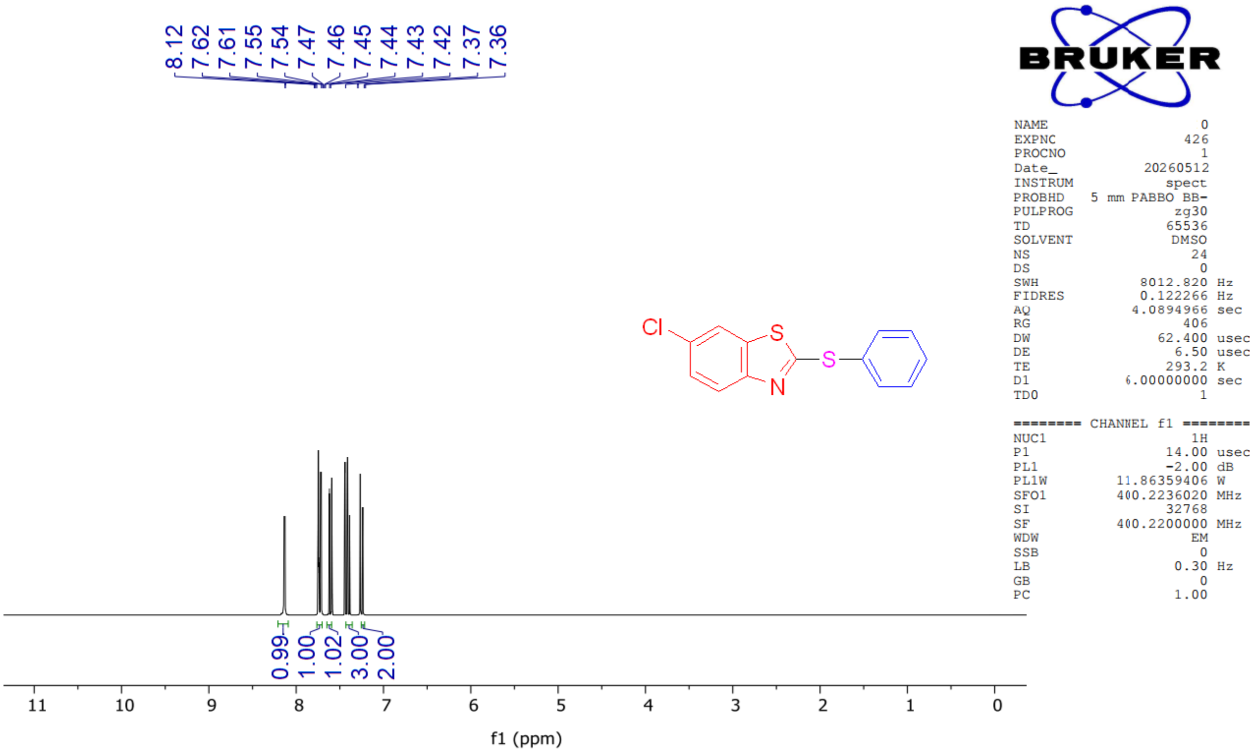
8.12
7.62
7.61
7.55
7.54
7.47
7.46
7.45
7.44
7.43
7.42
7.37
7.36
BRUKER
NAME 0
EXPNO 426
PROCNO 1
Date_ 20260512
INSTRUM spect
PROBHD 5 mm PABBO BB-
PULPROG zg30
TD 65536
SOLVENT DMSO
NS 24
DS 0
SWH 8012.820 Hz
FIDRES 0.122266 Hz
AQ 4.0894966 sec
RG 406
DW 62.400 usec
DE 6.50 usec
TE 293.2 K
D1 6.00000000 sec
TD0 1
======== CHANNEL f1 ========
NUC1 1H
P1 14.00 usec
PL1 -2.00 dB
PL1W 11.86359406 W
SFO1 400.2236020 MHz
SI 32768
SF 400.2200000 MHz
WDW EM
SSB 0
LB 0.30 Hz
GB 0
PC 1.00
Cl
S
N
S
0.99
1.00
1.02
3.00
2.00
11 10 9 8 7 6 5 4 3 2 1 0
f1 (ppm)

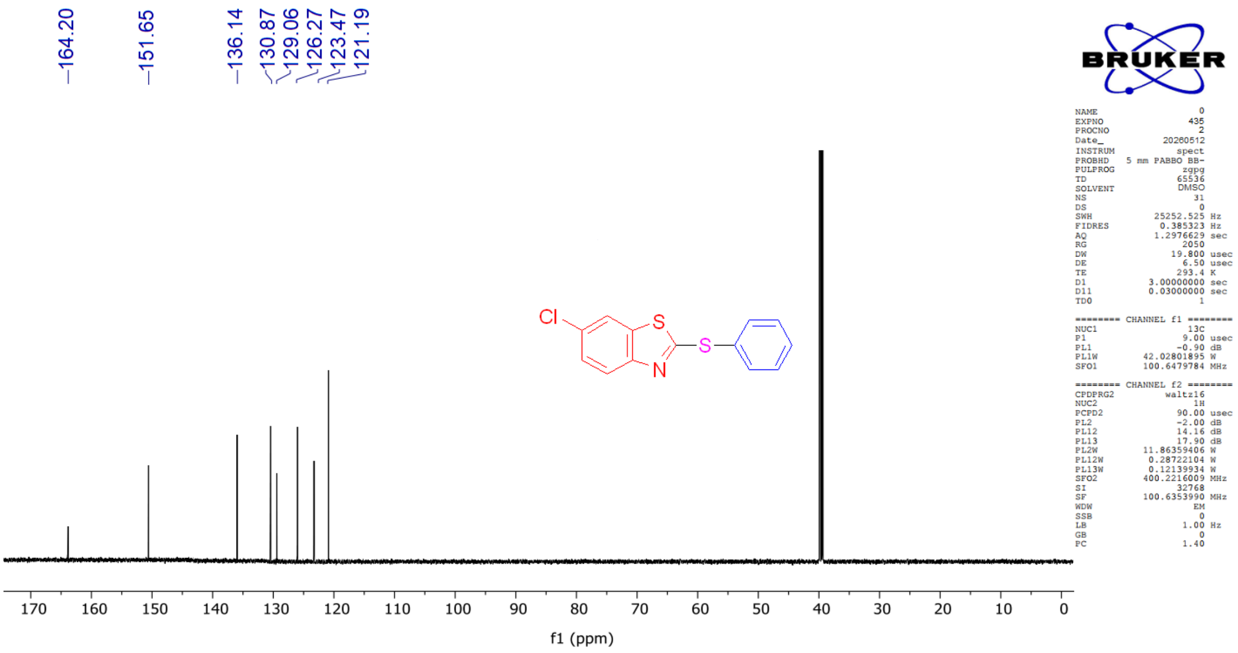
164.20
151.65
136.14
130.87
129.06
126.27
123.47
121.19
BRUKER
NAME 0
EXPNO 435
PROCNO 2
Date_ 20260512
INSTRUM spect
PROBHD 5 mm PABBO BB-
PULPROG zgpg
TD 65536
SOLVENT DMSO
NS 31
DS 0
SWH 25252.525 Hz
FIDRES 0.385323 Hz
AQ 1.2976629 sec
RG 2050
DW 19.800 usec
DE 6.50 usec
TE 293.4 K
D1 3.00000000 sec
D11 0.03000000 sec
TD0 1
======== CHANNEL f1 ========
NUC1 13C
P1 9.00 usec
PL1 -0.90 dB
PL1W 42.02801895 W
SFO1 100.6479784 MHz
======== CHANNEL f2 ========
CPDPRG2 waltz16
NUC2 1H
PCPD2 90.00 usec
PL2 -2.00 dB
PL12 14.16 dB
PL13 17.90 dB
PL2W 11.86359406 W
PL12W 0.28722104 W
PL13W 0.12139934 W
SFO2 400.2216009 MHz
SI 32768
SF 100.6353990 MHz
WDW EM
SSB 0
LB 1.00 Hz
GB 0
PC 1.40
Cl
S
N
S
170 160 150 140 130 120 110 100 90 80 70 60 50 40 30 20 10 0
f1 (ppm)

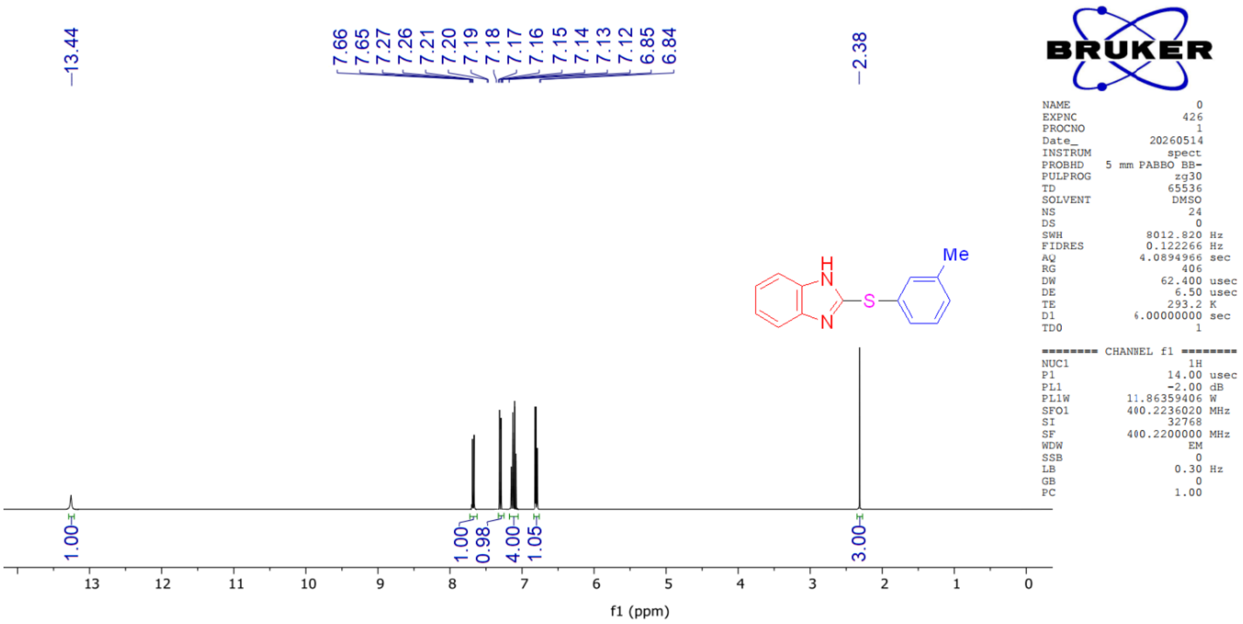
13.44
7.66
7.65
7.27
7.26
7.21
7.20
7.19
7.18
7.17
7.16
7.15
7.14
7.13
7.12
6.85
6.84
2.38
BRUKER
NAME 0
EXPNO 426
PROCNO 1
Date_ 20260514
INSTRUM spect
PROBHD 5 mm PABBO BB-
PULPROG zg30
TD 65536
SOLVENT DMSO
NS 24
DS 0
SWH 8012.820 Hz
FIDRES 0.122266 Hz
AQ 4.0894966 sec
RG 406
DW 62.400 usec
DE 6.50 usec
TE 293.2 K
D1 6.00000000 sec
TD0 1
======== CHANNEL f1 ========
NUC1 1H
P1 14.00 usec
PL1 -2.00 dB
PL1W 11.86359406 W
SFO1 400.2236020 MHz
SI 32768
SF 400.2200000 MHz
WDW EM
SSB 0
LB 0.30 Hz
GB 0
PC 1.00
H
N
N
S
Me
1.00
1.00
0.98
4.00
1.05
3.00
13 12 11 10 9 8 7 6 5 4 3 2 1 0
f1 (ppm)

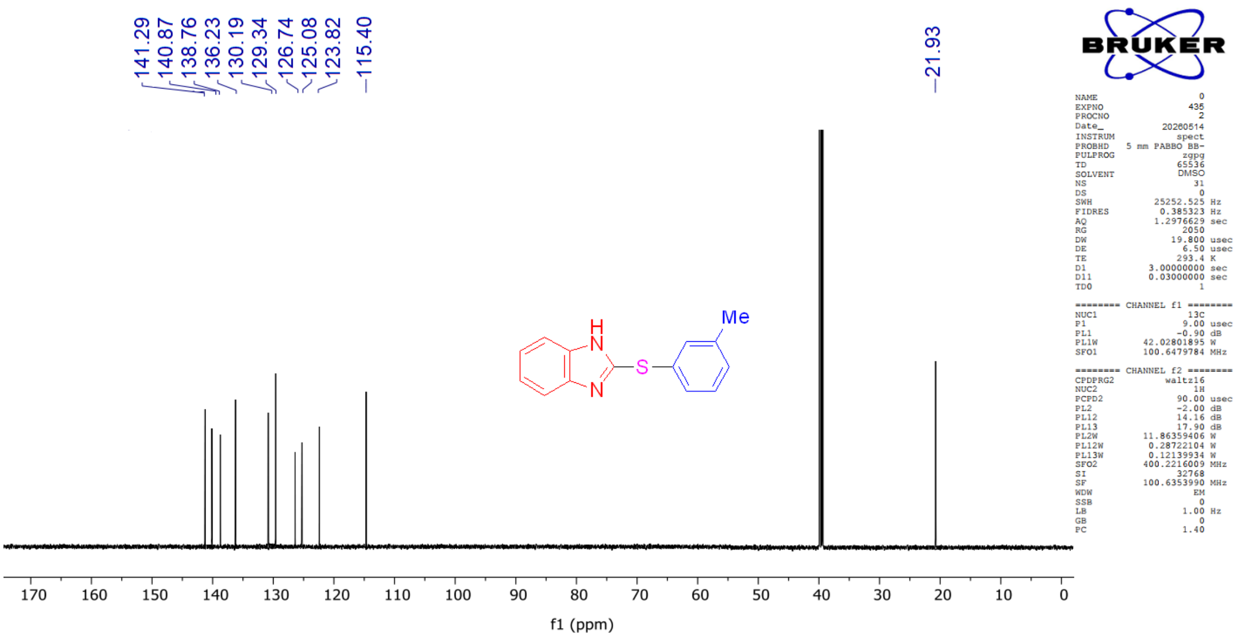
141.29
140.87
138.76
136.23
130.19
129.34
126.74
125.08
123.82
115.40
21.93
BRUKER
NAME 0
EXPNO 435
PROCNO 2
Date_ 20260514
INSTRUM spect
PROBHD 5 mm PABBO BB-
PULPROG zgpg
TD 65536
SOLVENT DMSO
NS 31
DS 0
SWH 25252.525 Hz
FIDRES 0.385323 Hz
AQ 1.2976629 sec
RG 2050
DW 19.800 usec
DE 6.50 usec
TE 293.4 K
D1 3.00000000 sec
D11 0.03000000 sec
TD0 1
======== CHANNEL f1 ========
NUC1 13C
P1 9.00 usec
PL1 -0.90 dB
PL1W 42.02801895 W
SFO1 100.6479784 MHz
======== CHANNEL f2 ========
CPDPRG2 waltz16
NUC2 1H
PCPD2 90.00 usec
PL2 -2.00 dB
PL12 14.16 dB
PL13 17.90 dB
PL2W 11.86359406 W
PL12W 0.28722104 W
PL13W 0.12139934 W
SFO2 400.2216009 MHz
SI 32768
SF 100.6353990 MHz
WDW EM
SSB 0
LB 1.00 Hz
GB 0
PC 1.40
H
N
N
S
Me
170 160 150 140 130 120 110 100 90 80 70 60 50 40 30 20 10 0
f1 (ppm)

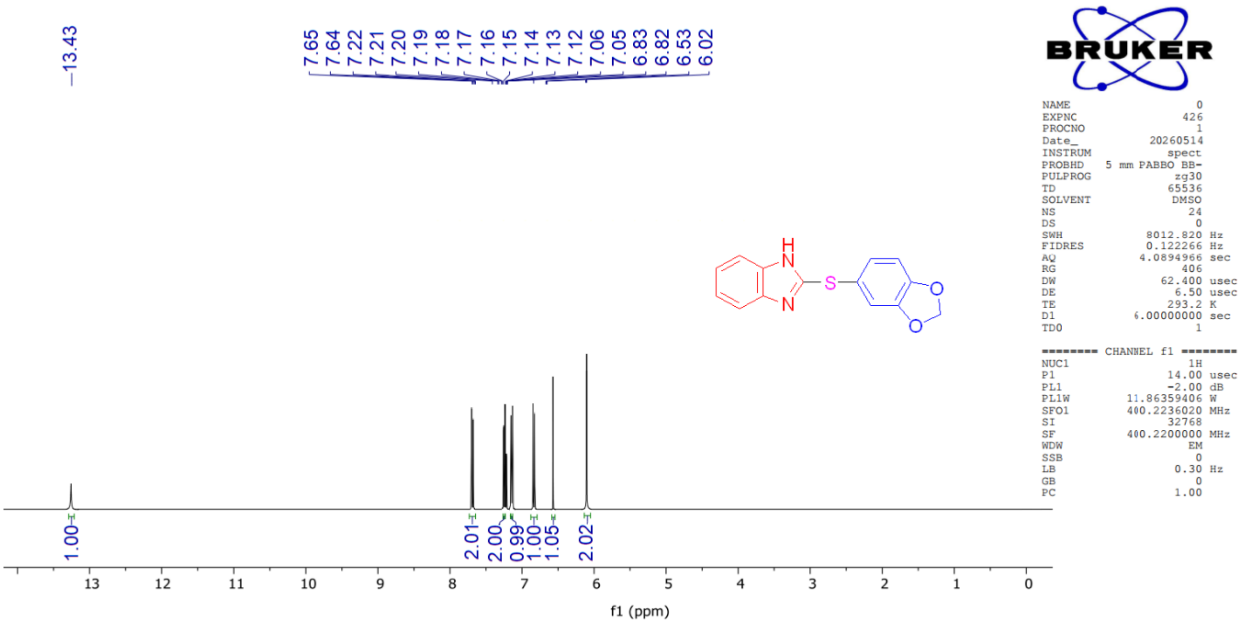
13.43
7.65
7.64
7.22
7.21
7.20
7.19
7.18
7.17
7.16
7.15
7.14
7.13
7.12
7.06
7.05
6.83
6.82
6.53
6.02
BRUKER
NAME 0
EXPNO 426
PROCNO 1
Date_ 20260514
INSTRUM spect
PROBHD 5 mm PABBO BB-
PULPROG zg30
TD 65536
SOLVENT DMSO
NS 24
DS 0
SWH 8012.820 Hz
FIDRES 0.122266 Hz
AQ 4.0894966 sec
RG 406
DW 62.400 usec
DE 6.50 usec
TE 293.2 K
D1 6.00000000 sec
TD0 1
======== CHANNEL f1 ========
NUC1 1H
P1 14.00 usec
PL1 -2.00 dB
PL1W 11.86359406 W
SFO1 400.2236020 MHz
SI 32768
SF 400.2200000 MHz
WDW EM
SSB 0
LB 0.30 Hz
GB 0
PC 1.00
H
N
N
S
O
O
1.00
2.01
2.00
0.99
1.00
1.05
2.02
13 12 11 10 9 8 7 6 5 4 3 2 1 0
f1 (ppm)

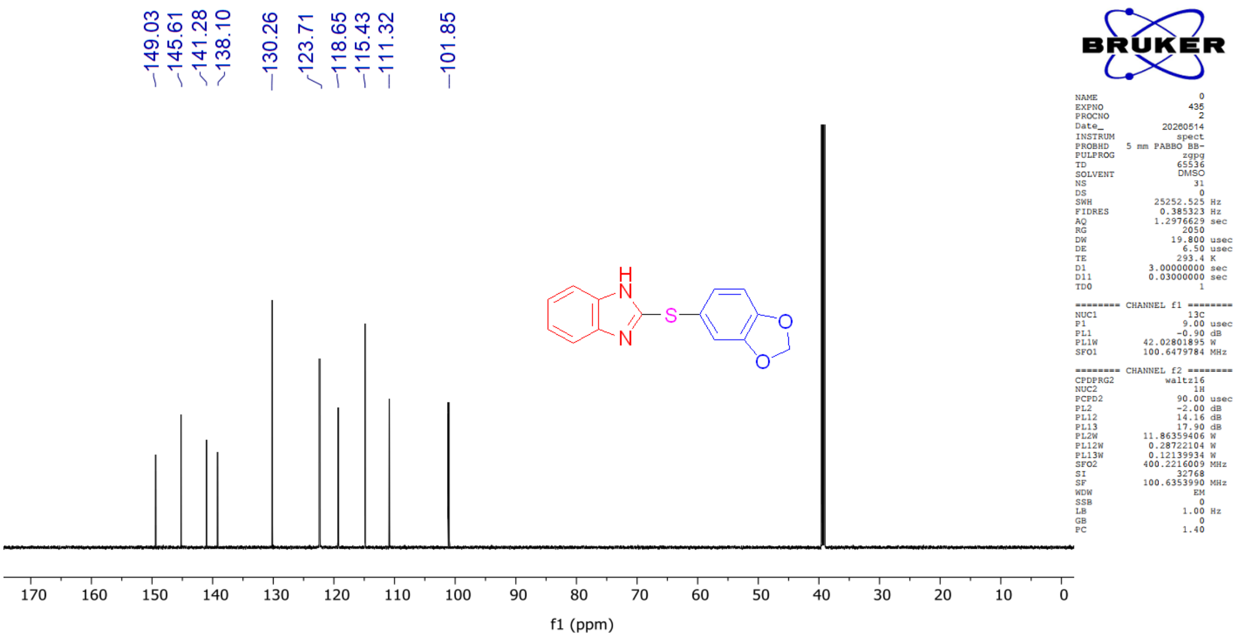
149.03
145.61
141.28
138.10
130.26
123.71
118.65
115.43
111.32
101.85
H
N
N
S
O
O
BRUKER
NAME 0
EXPNO 435
PROCNO 2
Date_ 20260514
INSTRUM spect
PROBHD 5 mm PABBO BB-
PULPROG zgpg
TD 65536
SOLVENT DMSO
NS 31
DS 0
SWH 25252.525 Hz
FIDRES 0.385323 Hz
AQ 1.2976629 sec
RG 2050
DW 19.800 usec
DE 6.50 usec
TE 293.4 K
D1 3.00000000 sec
D11 0.03000000 sec
TD0 1
======== CHANNEL f1 ========
NUC1 13C
P1 9.00 usec
PL1 -0.90 dB
PL1W 42.02801895 W
SFO1 100.6479784 MHz
======== CHANNEL f2 ========
CPDPRG2 waltz16
NUC2 1H
PCPD2 90.00 usec
PL2 -2.00 dB
PL12 14.16 dB
PL13 17.90 dB
PL2W 11.86359406 W
PL12W 0.28722104 W
PL13W 0.12139934 W
SFO2 400.2216009 MHz
SI 32768
SF 100.6353990 MHz
WDW EM
SSB 0
LB 1.00 Hz
GB 0
PC 1.40
170 160 150 140 130 120 110 100 90 80 70 60 50 40 30 20 10 0
f1 (ppm)

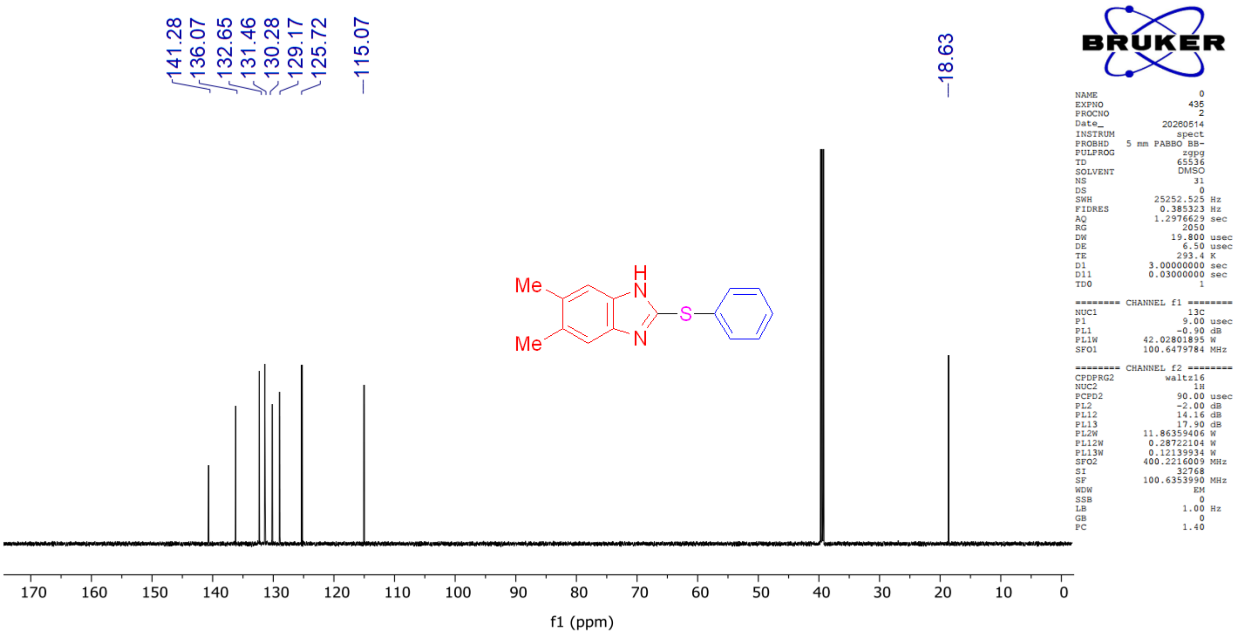
141.28
136.07
132.65
131.46
130.28
129.17
125.72
115.07
18.63
Me
Me
H
N
N
S
BRUKER
NAME 0
EXPNO 435
PROCNO 2
Date_ 20260514
INSTRUM spect
PROBHD 5 mm PABBO BB-
PULPROG zgpg
TD 65536
SOLVENT DMSO
NS 31
DS 0
SWH 25252.525 Hz
FIDRES 0.385323 Hz
AQ 1.2976629 sec
RG 2050
DW 19.800 usec
DE 6.50 usec
TE 293.4 K
D1 3.00000000 sec
D11 0.03000000 sec
TD0 1
======== CHANNEL f1 ========
NUC1 13C
P1 9.00 usec
PL1 -0.90 dB
PL1W 42.02801895 W
SFO1 100.6479784 MHz
======== CHANNEL f2 ========
CPDPRG2 waltz16
NUC2 1H
PCPD2 90.00 usec
PL2 -2.00 dB
PL12 14.16 dB
PL13 17.90 dB
PL2W 11.86359406 W
PL12W 0.28722104 W
PL13W 0.12139934 W
SFO2 400.2216009 MHz
SI 32768
SF 100.6353990 MHz
WDW EM
SSB 0
LB 1.00 Hz
GB 0
PC 1.40
170 160 150 140 130 120 110 100 90 80 70 60 50 40 30 20 10 0
f1 (ppm)

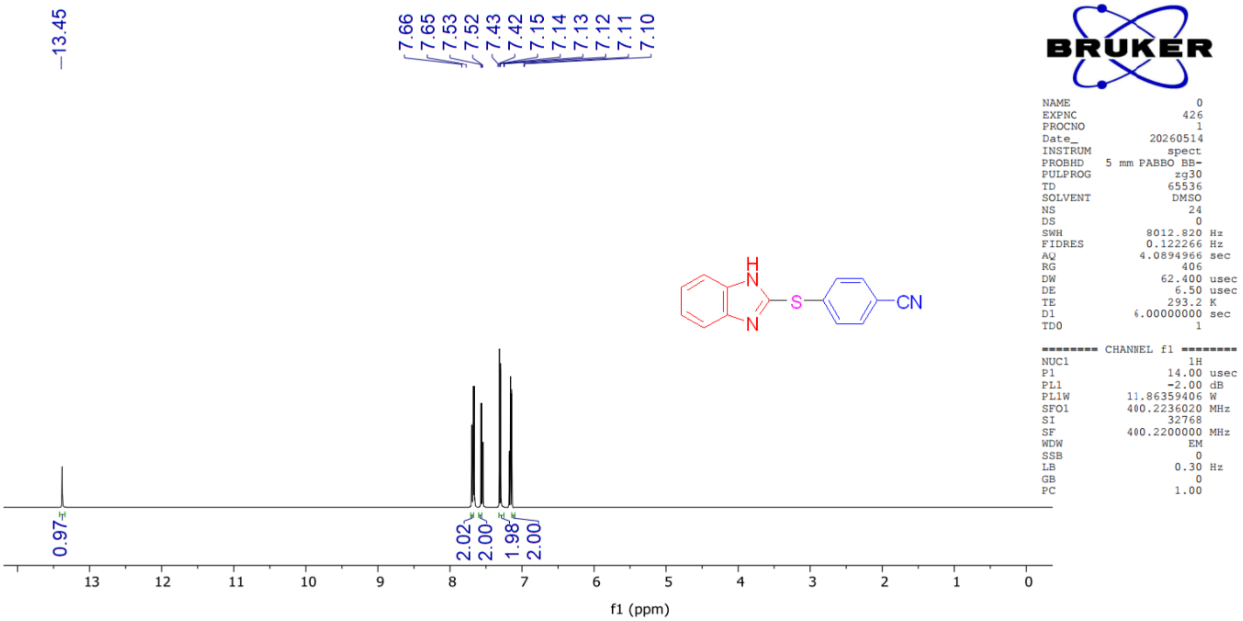

13.45
7.66
7.65
7.53
7.52
7.43
7.42
7.15
7.14
7.13
7.12
7.11
7.10
BRUKER
NAME 0
EXPNC 426
PROCNO 1
Date_ 20260514
INSTRUM spect
PROBHD 5 mm PABBO BB-
PULPROG zg30
TD 65536
SOLVENT DMSO
NS 24
DS 0
SWH 8012.820 Hz
FIDRES 0.122266 Hz
AQ 4.0894966 sec
RG 406
DW 62.400 usec
DE 6.50 usec
TE 293.2 K
D1 6.00000000 sec
TD0 1
======== CHANNEL f1 ========
NUC1 1H
P1 14.00 usec
PL1 -2.00 dB
PL1W 11.86359406 W
SFO1 400.2236020 MHz
SI 32768
SF 400.2200000 MHz
WDW EM
SSB 0
LB 0.30 Hz
GB 0
PC 1.00
H
N
N
S
CN
0.97
2.02
2.00
1.98
2.00
13
12
11
10
9
8
7
6
5
4
3
2
1
0
f1 (ppm)

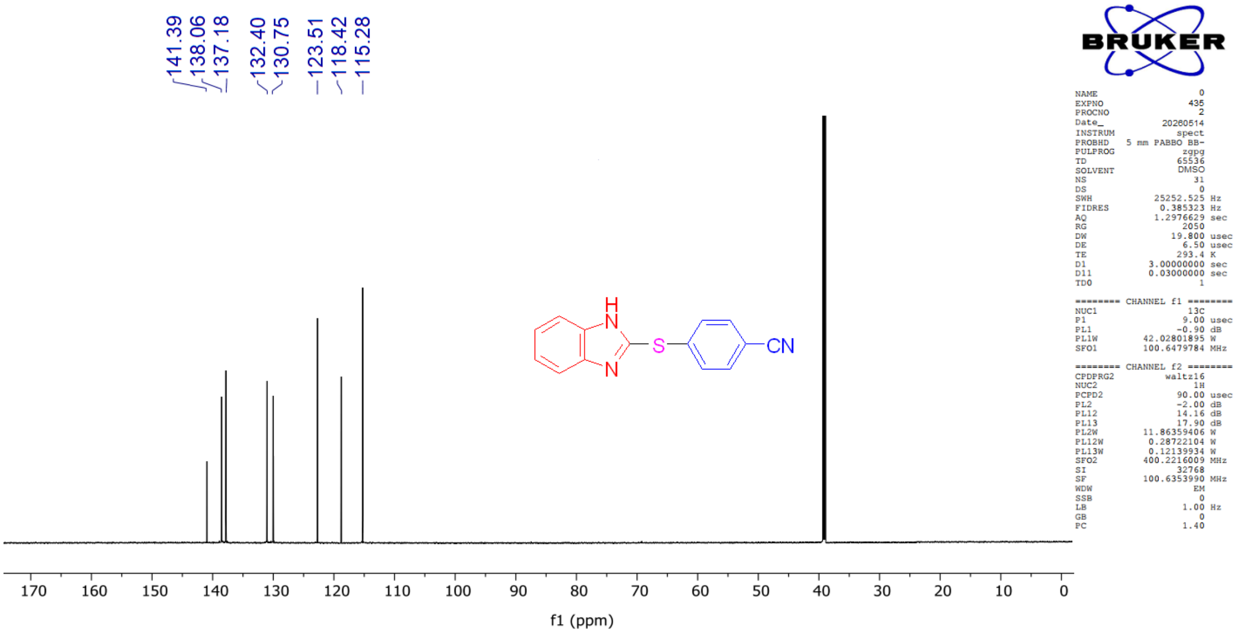

141.39
138.06
137.18
132.40
130.75
123.51
118.42
115.28
BRUKER
NAME 0
EXPNO 435
PROCNO 2
Date_ 20260514
INSTRUM spect
PROBHD 5 mm PABBO BB-
PULPROG zgpg
TD 65536
SOLVENT DMSO
NS 31
DS 0
SWH 25252.525 Hz
FIDRES 0.385323 Hz
AQ 1.2976629 sec
RG 2050
DW 19.800 usec
DE 6.50 usec
TE 293.4 K
D1 3.00000000 sec
D11 0.03000000 sec
TD0 1
======== CHANNEL f1 ========
NUC1 13C
P1 9.00 usec
PL1 -0.90 dB
PL1W 42.02801895 W
SFO1 100.6479784 MHz
======== CHANNEL f2 ========
CPDPRG2 waltz16
NUC2 1H
PCPD2 90.00 usec
PL2 -2.00 dB
PL12 14.16 dB
PL13 17.90 dB
PL2W 11.86359406 W
PL12W 0.28722104 W
PL13W 0.12139934 W
SFO2 400.2216009 MHz
SI 32768
SF 100.6353990 MHz
WDW EM
SSB 0
LB 1.00 Hz
GB 0
PC 1.40
H
N
N
S
CN
170
160
150
140
130
120
110
100
90
80
70
60
50
40
30
20
10
0
f1 (ppm)
